# Granulocyte colony-stimulating factor alters the systemic metabolomic profile in healthy donors

**DOI:** 10.1007/s11306-016-1139-x

**Published:** 2016-11-28

**Authors:** Kimberley Joanne Hatfield, Guro Kristin Melve, Øystein Bruserud

**Affiliations:** 1 Department of Clinical Science, University of Bergen, 5021 Bergen, Norway; 2 Department of Immunology and Transfusion Medicine, Haukeland University Hospital, Bergen, Norway; 3 Section for Hematology, Department of Medicine, Haukeland University Hospital, Bergen, Norway

**Keywords:** Allogeneic stem cell transplantation, Biochemical, Granulocyte colony-stimulating factor, Metabolomics, Stem cell donor

## Abstract

**Introduction:**

Peripheral blood stem cells mobilized by granulocyte colony-stimulating factor (G-CSF) from healthy donors are commonly used for allogeneic stem cell transplantation. The effect of G-CSF administration on global serum metabolite profiles has not been investigated before.

**Objectives:**

This study aims to examine the systemic metabolomic profiles prior to and following administration of G-CSF in healthy adults.

**Methods:**

Blood samples were collected from 15 healthy stem cell donors prior to and after administration of G-CSF 10 µg/kg/day for 4 days. Using a non-targeted metabolomics approach, metabolite levels in serum were determined using ultrahigh performance liquid chromatography-tandem mass spectrometry and gas chromatography/mass spectrometry.

**Results:**

Comparison of the metabolite profiles of donors before and after G-CSF treatment revealed 239 metabolites that were significantly altered. The major changes of the metabolite profiles following G-CSF administration included alteration of several fatty acids, including increased levels of several medium and long-chain fatty acids, as well as polyunsaturated fatty acids; while there were lower levels of other lipid metabolites such as phospholipids, lysolipids, sphingolipids. Furthermore, there were significantly lower levels of several amino acids and/or their metabolites, including several amino acids with known immunoregulatory functions (methionine, tryptophan, valine). Lastly, the levels of several nucleotides and nucleotide metabolites (guanosine, adenosine, inosine) were also decreased after G-CSF administration, while methylated products were increased. Some of these altered products/metabolites may potentially have angioregulatory effects whereas others may suggest altered intracellular epigenetic regulation.

**Conclusion:**

Our results show that G-CSF treatment alters biochemical serum profiles, in particular amino acid, lipid and nucleotide metabolism. Additional studies are needed to further evaluate the relevance of these changes in healthy donors.

**Electronic supplementary material:**

The online version of this article (doi:10.1007/s11306-016-1139-x) contains supplementary material, which is available to authorized users.

## Introduction

Peripheral blood stem cell (PBSC) grafts are commonly used for allogeneic hematopoietic stem cell transplantation for a wide range of hematologic malignancies. These grafts are generally prepared by administration of granulocyte colony-stimulating factor (G-CSF) to healthy donors which mobilizes hematopoietic stem cells (HSCs) into the blood before cells are harvested by leukapheresis. The clinical advantage of using PBSCs compared to bone marrow grafts includes accelerated neutrophil engraftment, which increases the likelihood to engraft, and in addition PBSC allografts contain a much larger number of T-cells compared to bone marrow grafts, which has been correlated with better outcome (Malard et al. 2016; Pabst et al. 2007; Rezvani et al. 2006).

The HSC mobilizing agent G-CSF is a glycoprotein with multiple functions, including effects on the production, migration, differentiation and proliferation of neutrophils, as well as affecting adaptive immune responses (Bendall and Bradstock 2014; Panopoulos and Watowich 2008). G-CSF may have both direct and indirect effects on immune cells, including monocytes, granulocytes, T-cells and dendritic cells, and can also alter the expression of various soluble factors, including cytokines, metalloproteinases and adhesion molecules which may themselves contribute to effects induced by G-CSF (Rutella et al. 2005). Recently, various metabolites and metabolic pathways have been found to be involved in cell signaling, also among immune cells; e.g. both amino acids as well as their metabolites can bind to specific receptors on immunocompetent cells and thereby induce activation and/or differentiation of these cells (Buck et al. 2015). Furthermore, certain metabolites have a key role in fundamental metabolic pathways, such as glycolysis or lipid metabolism, and their availability may thus affect immune cell functions. As reviewed by Buck et al. (2015), cellular metabolism is important in the regulation of immunocompetent cell growth, e.g. differentiation and activation of T-cells. Thus, the availability and uptake of metabolites may potentially affect immune cell fate. In this aspect, metabolomics has emerged as a powerful tool to identify and characterize the low molecular mass composition of biological samples. In this exploratory study, we therefore used non-targeted metabolomics to investigate the early effects of in vivo G-CSF treatment on the global serum metabolite profile of healthy stem cell donors.

## Materials and methods

### Stem cell donors and mobilization

The study was approved by the local ethics committee (REK Vest, 2011/996 and 2011/1237) and all samples were collected after written informed consent. Blood samples were collected from 15 consecutive healthy HLA-matched related allogeneic stem cell donors (10 males and 5 females), with a mean age of 47 years (range 25–64 years) (Table 1). Donors received the human non-glycosylated G-CSF analog Filgrastim (r-metHuG-CSF, Neupogen, Amgen) or Tevagrastim (biosimilar Filgrastim) in a dose of 5.4 μg/kg body weight (range 4.1–6.7 μg/kg) twice daily subcutaneously for four days to induce stem cell mobilization. Our hospital is responsible for all allogeneic stem cell transplantations in a defined geographic area, and this study included a consecutive group of donors younger than 65 years of age and achieving pre-harvest CD34^+^ cell counts above 15 × 10^3^/L. Thus, our donors should be regarded as representative of healthy adult stem cell donors because they are unselected (i.e. consecutive), mobilize sufficient stem cells for preparation of allografts and their age is also representative for donors used in routine clinical practice.

**Table 1:** Characteristics of allogeneic stem cell donors

ID	Age (years)	Gender	BMI (kg/m^2^)	G-CSF dosage (µg/kg)	G-CSF (pg/mL) at clinical examination^a^	G-CSF (pg/mL) before apheresis^a^	CD34^+^ cell count (10^3^/L) pre-harvest^b^	CD34^+^ stem cell yield (× 10^6^/kg)^b^
1	60	F	30	6.1	<20	>15,000	40.2	5.5
2	25	M	24	6.1	<20	13,514	44.4	8.8
3	45	M	25	5.8	242	>15,000	30.4	4.3
4	51	M	47	4.9	37	>15,000	58.8	3.9
5	39	M	30	5.3	72	13,404	108.8	22.4
6	64	M	36	4.1	<20	9495	26.7	3.9
7	54	F	23	5.7	41	>15,000	34.1	5.2
8	25	M	26	5.7	111	>15,000	147.8	15.1
9	46	F	25	4.6	49	3939	57.6	7.2
10	62	M	27	5.6	24	>15,000	97.0	7.2
11	51	M	26	5.1	53	>15,000	17.4	3.1
12	40	F	34	5.3	39	>15,000	66.7	6.8
13	39	M	25	5.5	22	>15,000	111.1	7.8
14	45	F	26	6.7	82	>15,000	55.1	5.6
15	58	M	29	5.1	80	6776	44.7	4.9
Mean	47		29	5.4	61	>13,000	62.7	8.7
Range	25–64	10M/5F	23–47	4.1–6.7	<20–242	3939– > 15,000	17.4–147.8	3.1–22.4

*M* male; *F* female; *BMI* body mass index

^a^G-CSF plasma levels were measured in donor samples collected at clinical examination and after four days of treatment with G-CSF before apheresis

^b^CD34^+^ cell counts were done immediately before stem cell harvest and the CD34^+^ stem cell yield estimated per kg donor/weight

### Processing of blood samples

Venous blood samples were collected into Vacuette Z Serum Clot Activator tubes with Gel Separator (Greiner Bio-One GmbH, Kremsmünster, Austria) from donors at two time points, (i) prior to administration of G-CSF and (ii) following G-CSF administration just before apheresis on day 4. All samples were collected at 9 am and were allowed to coagulate for 30 min at room temperature in upright position before being centrifuged at 1310×*g* for 10 min at room temperature. The serum supernatants were immediately apportioned into 0.5 mL aliquots in plastic cryotubes (Nunc™, Roskilde, Denmark) and stored frozen at −80°C until analysed.

### Analysis of G-CSF levels

Levels of human G-CSF were measured using a Luminex assay (R&D Systems, Bio-techne, Abingdon, UK), and the minimal detectable level was 20 pg/mL.

### Analysis of human serum metabolites

All mass spectrometry data were collected at Metabolon Inc (Durham, NC). Each serum sample was accessioned into the Metabolon LIMS system and was assigned a unique identifier by this system which was used to track all sample handling and results. All samples were prepared using the automated MicroLab STAR^®^ system (Hamilton Company, Bonaduz, Switzerland). Briefly, samples were extracted using Metabolon`s standard solvent extraction method (Evans et al. 2014). A recovery standard was added prior to the first step in the extraction process for quality control purposes. To remove protein, dissociate small molecules bound to protein or trapped in the precipitated protein matrix, and to recover chemically diverse metabolites, proteins were precipitated with methanol under vigorous shaking for 2 min followed by centrifugation. The resulting extract was divided into five fractions: (i) one for analysis by ultrahigh performance liquid chromatography–tandem mass spectrometry (UPLC–MS/MS) with positive ion mode electrospray ionization, (ii) one for analysis by UPLC–MS/MS with negative ion mode electrospray ionization, (iii) one for LC polar platform, (iv) one for analysis by gas chromatography/mass spectrometry (GC–MS) and (v) one sample was reserved for backup. Samples were placed briefly on a Zymark TurboVap^®^ (McKinley Scientific, Sparta, NJ, USA) to remove the organic solvent. Then samples were either stored overnight under nitrogen for LC or dried under vacuum overnight for GC, before preparation for analysis. Experimental samples were randomized across the platform and run with appropriate quality control samples spaced evenly among the injections. Compounds were identified by comparison to library entries based upon retention time/index, mass to charge ratio (m/z) and chromatographic data (also MS/MS spectral data), and peaks were quantified using area-under-the curve.

### Statistical analyses

Two types of statistical analysis were performed: (1) significance tests (t-tests) and (2) classification analysis. Random Forest analysis is a supervised classification technique that provides an unbiased estimate of how well individuals can be classified into each group in a new data set. Statistical analyses were performed with the program R (http://cran.r-project.org/).

## Results

### G-CSF treatment alters the global metabolomic profile of healthy individuals

Metabolites were analysed in all serum samples collected from the healthy donors (i) prior to G-CSF administration and (ii) on day 4 after G-CSF administration. In total, 641 metabolites were identified (for a complete list see Supplementary Table 1), where levels of 239 metabolites were significantly changed (p ≤ 0.05); 149 metabolites had increased levels (62%) and 90 metabolites had decreased levels (38%) after G-CSF administration (Table 2). These significantly altered metabolites belong mainly to amino acid and lipid classes, while metabolites associated with the categories nucleotides, carbohydrates, energy metabolism, cofactors/vitamins and xenobiotics are also present. Furthermore, 39 of these metabolites have a p value < 0.0001 and are involved in amino acid (19/39), nucleotide (10/39) or lipid (5/39) metabolism (Table 2). Among the significantly altered levels of metabolites (n = 239, p ≤ 0.05) we would expect to see approximately 12 metabolites that meet our level of significance criteria by random chance, however, our data have a low false discovery rate (FDR) of less than 5% for all metabolites except one metabolite with 5.1%, indicating a high level of confidence in the results (Supplementary Table 2). All significantly altered metabolites (p ≤ 0.05) and their group mean ratios (before vs. after G-CSF treatment) are shown in Supplementary Table 2.

**Table 2:** Metabolite classes significantly altered after G-CSF treatment

Metabolite classes/pathways	Total number of metabolites identified	Number of significantly altered metabolites		
		P < 0.05	P < 0.001	P < 0.0001
Amino acids	161	75	30	19
Peptide	28	16	1	1
Carbohydrates	25	8	5	2
Energy metabolism	9	2	−	−
Lipids	250	78	13	5
Nucleotides	34	21	11	10
Cofactors-vitamins	22	10	4	−
Xenobiotics	112	29	4	2
Total number of metabolites	641	239	68	39

**Fig. 1:**
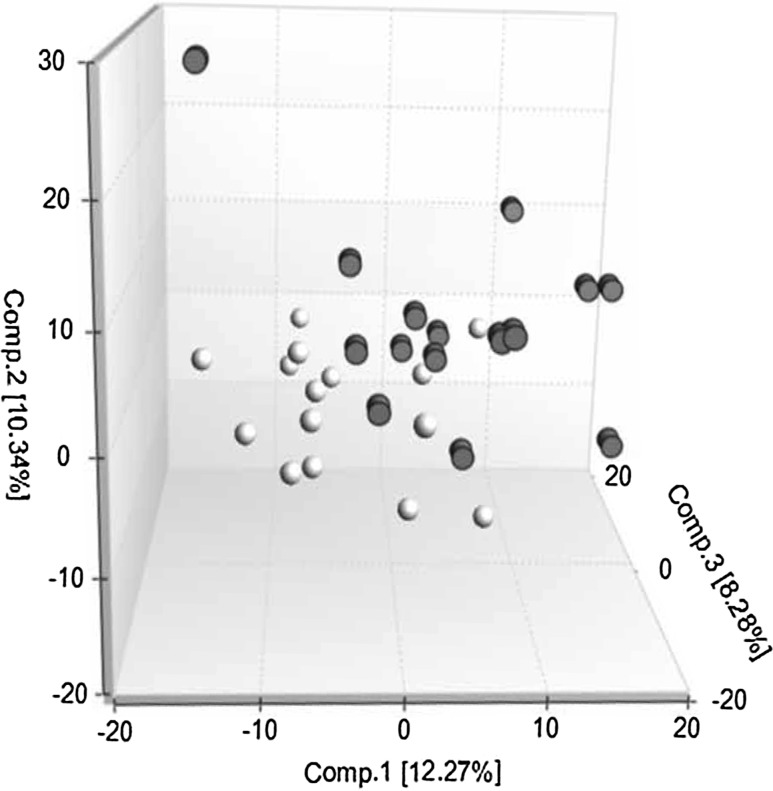
Principal component analysis (PCA) scores plot based on the serum metabolome of healthy stem cell donors before and after G-CSF treatment. An overlap was seen between groups (n = 15 in each group, *open circles* before G-CSF treatment, *filled circles* after G-CSF treatment), but groups were generally distinguishable from each other

We performed a principal component analysis (PCA) which showed that the samples before and after G-CSF administration were generally distinguishable from each other (Fig. 1); one G-CSF treated sample seemed to deviate from the other samples but no outlier samples were identified. This exceptional stem cell donor had the highest total leukocyte and platelet counts in peripheral blood, the lowest Hb level after G-CSF administration and the lowest G-CSF plasma level before apheresis, but did not otherwise differ from the others, and samples from this donor were included in all our analyses. Taken together, these results demonstrate that four days of G-CSF administration alters the systemic metabolomic profile of healthy individuals; and despite some heterogeneity between donors, an altered amino acid, lipid and nucleotide metabolism seems to be a common characteristic.

### Alteration of single metabolites by G-CSF treatment

**Fig. 2:**
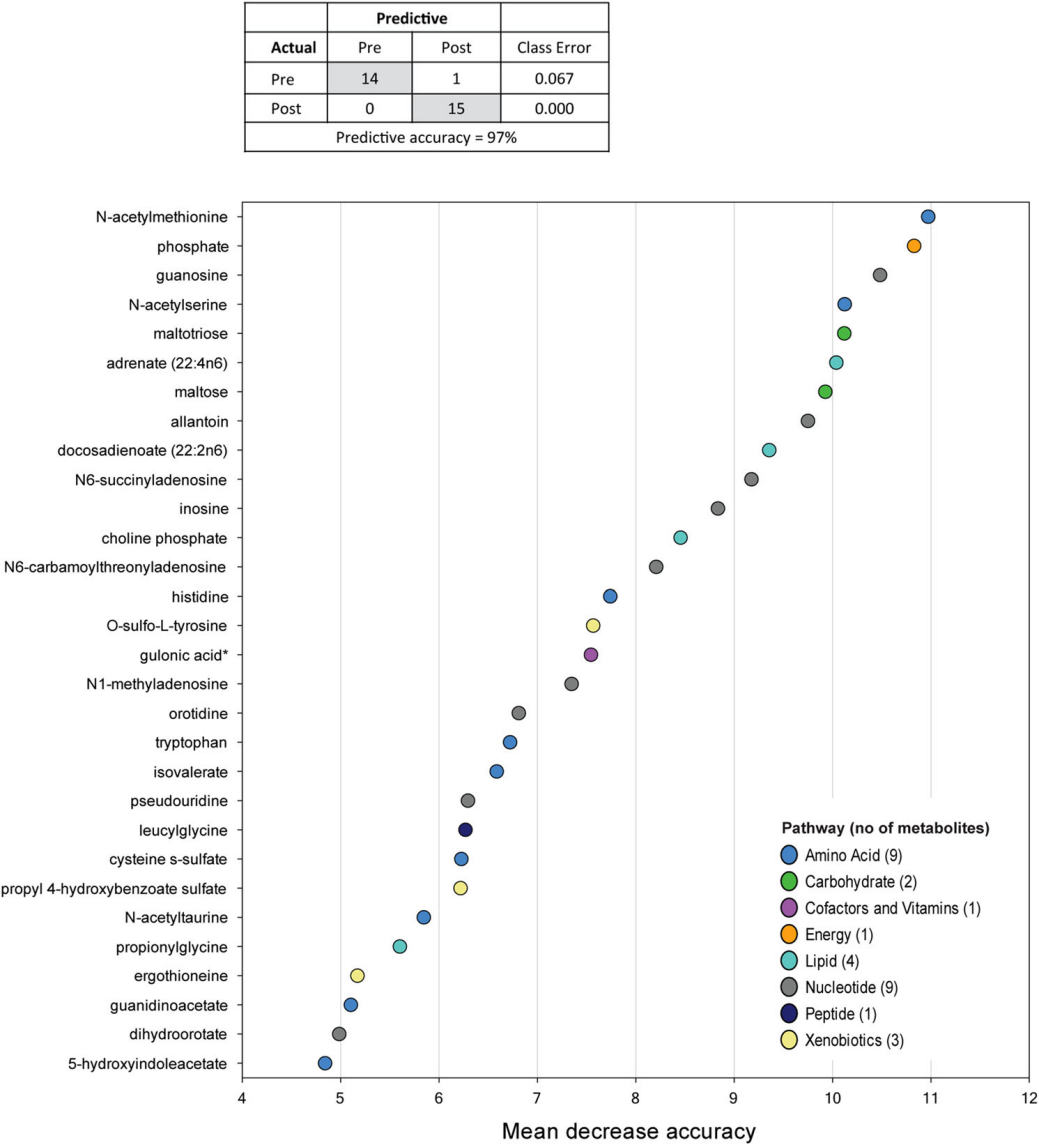
Random forest analysis of the metabolic profiles in samples taken before and after administration of G-CSF in healthy stem cell donors. Random forest analysis could distinguish between the metabolic profiles of the two groups with a predictive accuracy of 97%. The variable importance plot shows the variable on the y-axis, and their importance for separation of the two groups on the x-axis. The top-ranked 30 metabolites are thus ordered top-to-bottom as most- to least-important based on their importance. The *inset table* shows the main signaling pathways where each metabolite belongs, reflected by the different colors in the plot. *Asterisk* indicates that the biochemical name is identified but has not been confirmed based on a standard

Random forest classification was used for further statistical analyses. Even though there was an overlap between samples collected before and during G-CSF therapy in the PCA plot (Fig. 1), the random forest classification demonstrated that G-CSF treated versus untreated samples could be distinguished with 97% predictive accuracy based on their overall metabolite profiles (Fig. 2). The 30 top-ranking metabolites that contributed most to separation of the samples are shown where the metabolites are ranked according to the mean decrease accuracy (%) (Fig. 2). These metabolites are involved in several pathways, with the majority of metabolites belonging to amino acid metabolism (9 metabolites) and nucleotide metabolism (9 metabolites), but lipid (4 metabolites), xenobiotics (3 metabolites), carbohydrate (2 metabolites), cofactors/vitamin (1 metabolite) and peptides (1 metabolite) were also included among the 30 top-ranking metabolites in this analysis.

### Alteration of lipid, amino acid and nucleotide metabolism by G-CSF treatment

**Fig. 3:**
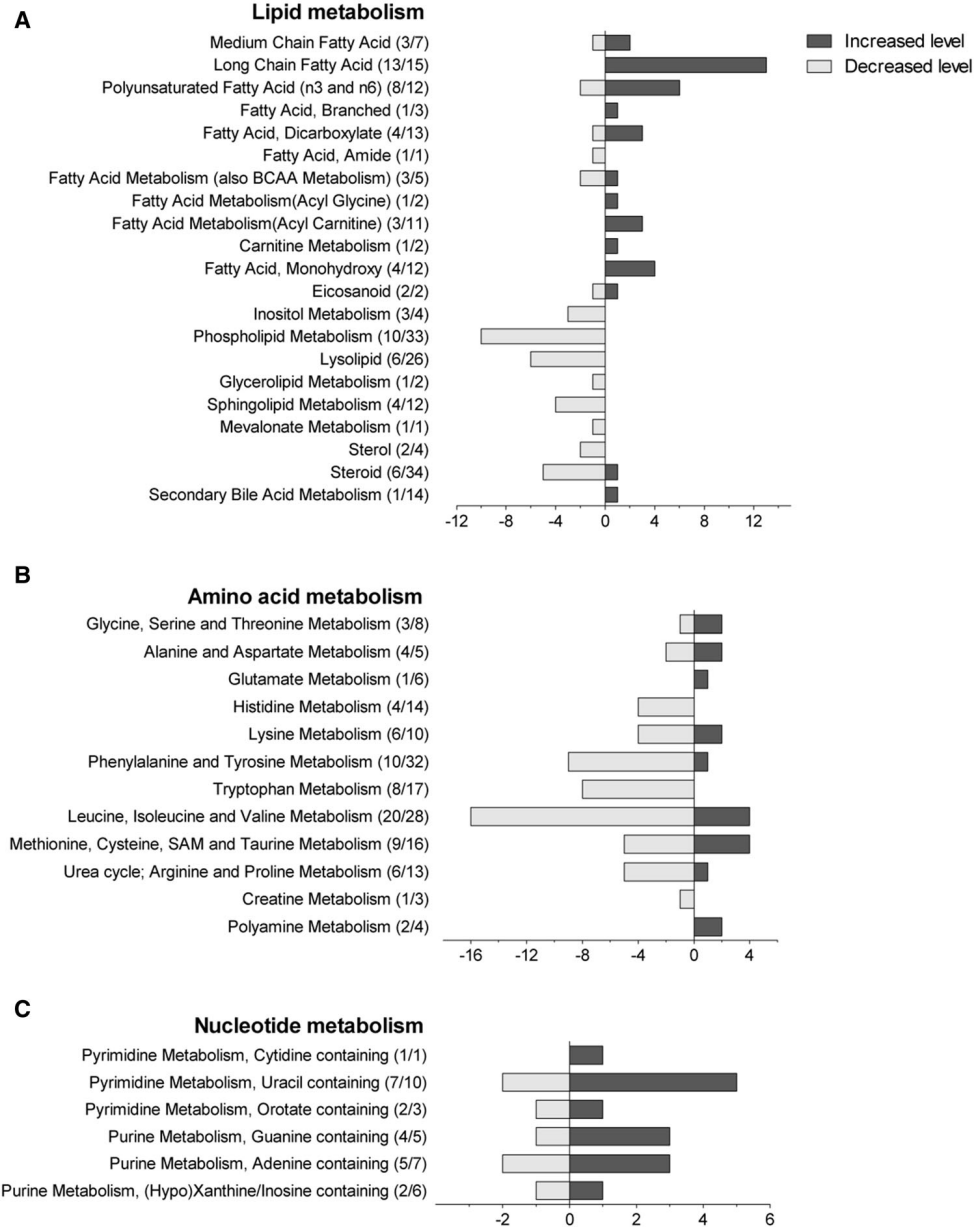
Metabolite pathways altered after G-CSF administration among the classes lipids, amino acids and nucleotides. The *bar charts* show the different amounts of metabolites belonging to their different pathways within the three main classes: **a** lipid metabolism, **b** amino acid metabolism and **c** nucleotide metabolism, where metabolite levels were either increased (*dark grey* colored) or decreased (*light grey*)
- *Amino acid metabolism* was also altered; within the post-G-CSF samples there was a reduced level of dipeptides and amino acids including the aromatic amino acids tryptophan, phenylalanine and tyrosine and their metabolites, as well as branched-chain amino acids valine, isoleucine and leucine (Fig. 3b). The essential amino acid tryptophan can be metabolized by several pathways to give rise to serotonin or kynurenine, and levels of these degradation products as well as indoleacetate and 3-indoxyl sulfate were all significantly lower after G-CSF therapy.
- *Nucleic acid metabolism* Our results show a significantly lower level of purine nucleosides, including guanosine, adenosine and inosine in the post-G-CSF samples, indicating altered nucleotide metabolism (Fig. 3c). Furthermore, several methylated products were increased.

Treatment with G-CSF resulted in altered systemic (i.e. serum) levels of a wide range of metabolites reflecting an alteration of different metabolic pathways during this treatment in healthy individuals:

- *Lipid metabolism* Among the main metabolites that distinguished between pre-and post-G-CSF administration groups, there was a consistent alteration of the amount of fatty acids indicating altered lipid metabolism (i.e. a change in fatty acid synthesis, lipid hydrolysis or mitochondrial β-oxidation) (Fig. 3a). The post-G-CSF group had significantly elevated levels of several long-chain fatty acids (such as myristate, palmitate, margarate, and stearate), as well as polyunsaturated fatty acids (adrenate, linoleate, linolenate, dihomolinoleate, docosadienoate, docosapentaenoate). Finally, levels of carnitine-conjugated lipids were increased.

### G-CSF induced alteration of metabolic pathways

**Fig. 4:**
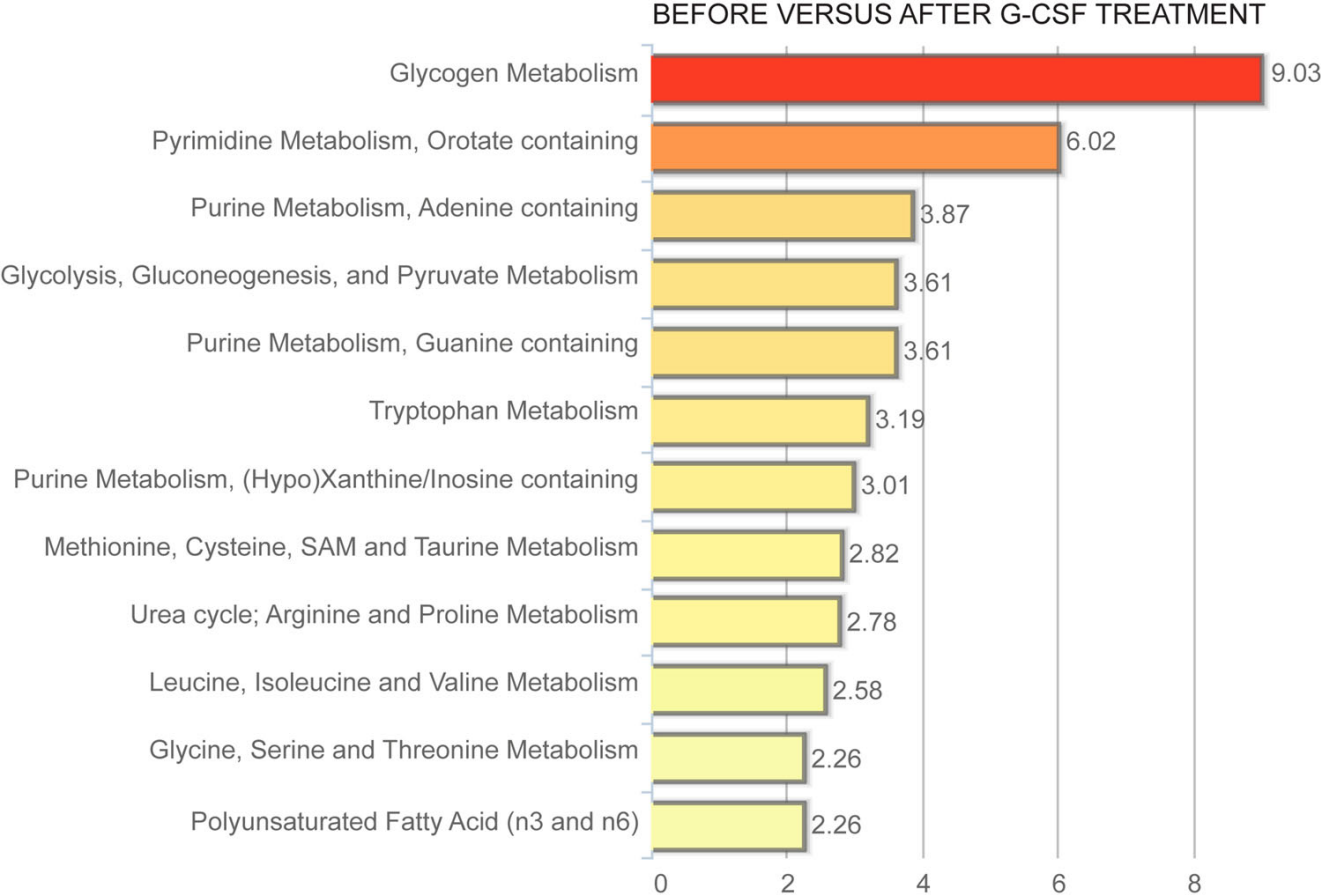
Metabolite pathway enrichment analysis to identify pathways enriched after G-CSF administration in healthy donors. A pathway enrichment analysis was done based on significantly altered metabolites with p < 0.001. Only signaling pathways with an enrichment value greater than two and at least two metabolites within each pathway are shown in the figure. The most significant p-values are seen in *red*, while the least significant are in *yellow*

We performed a metabolomic pathway enrichment analysis based on significantly altered metabolites (p < 0.001), to identify pathways that contribute to the major differences when comparing samples taken before and after G-CSF administration (Fig. 4). This analysis identified altered glycogen metabolism as a major effect of G-CSF treatment, and metabolites belonging to pathways involved in nucleotide metabolism and amino sugar/acid metabolism were also over-represented in samples after G-CSF treatment.

### The metabolic alterations induced during G-CSF therapy are not caused by acetaminophen

Musculoskeletal pain and flu-like symptoms are common during G-CSF therapy (Stroncek et al. 1996), and symptomatic relief can be achieved by acetaminophen (paracetamol). The detection of several metabolites of acetaminophen in the serum of stem cell donors indicates that paracetamol had been taken by some donors during G-CSF therapy. We therefore compared the metabolite profile before and after G-CSF therapy for donors with high and low/undetectable levels of metabolites involved in acetaminophen metabolism. When comparing the levels of the 30 top-ranked metabolites (Fig. 2), we did not find any significant differences between the donor samples with high and low/undetectable paracetamol metabolite (data not shown). Thus, it seems unlikely that the paracetamol intake has a major impact on the metabolic modulation during G-CSF therapy.

## Discussion

In this study, we investigated the early effects of G-CSF administration on the serum global metabolite profile of healthy stem cell donors that were younger than 65 years and had an adequate stem cell mobilization. G-CSF is mainly used as short-term therapy, generally requiring 4–6 days of treatment, for stem cell mobilization (Bendall and Bradstock 2014); however, it is also used as long-term treatment for patients with low-risk myelodysplastic syndrome (Jadersten et al. 2005) and especially for patients with chronic neutropenia (Dale 2016; Dale and Welte 2011; Donadieu et al. 2011; Zeidler et al. 2014). The suggested initial doses for congenital neutropenia are 3–5 µg/kg that are increased in steps of 5 µg/kg (Dale 2016; Donadieu et al. 2011), thus these G-CSF doses used in long-term therapy are also comparable to the doses used in our present study (5 µg/kg twice daily).

Our study included a relatively small number of samples, but our random forest classification analysis resulted in 97% predictive accuracy in differentiating the two groups, indicating that differences due to G-CSF administration were readily present. Several of the 30 top-ranking metabolites shown in Fig. 2 have been reported to be involved in biological processes such as regulation of immune responses, inflammation, vascular biology and epigenetic regulation (see Supplementary Table 3), though further studies will be needed to see if G-CSF has a long-term effect on these biological processes. In general, global metabolomics profiling revealed altered levels of lipids, amino acids, carbohydrates and nucleotides after administration with G-CSF.

One of the strongest changes in our dataset was the altered lipid metabolism, in particular the significantly higher levels of long-chain fatty acids as well as carnitine-conjugated lipids after G-CSF administration, indicating changes in fatty acid β-oxidation. Long-chain fatty acids are conjugated to carnitine to facilitate transport across the mitochondrial membrane, and the increased acyl carnitine levels may thus suggest increased β-oxidation. Moreover, glycerol, a marker of lipolysis, was significantly decreased in the G-CSF treated group. These alterations may be due to increased fatty acid β-oxidation or alternatively due to disturbance of fatty acid oxidation resulting in increased amounts of lipid precursors. In addition, we found a decline in the serum levels of sphingosine 1-phosphate (S1P) after G-CSF administration, which is in concordance with another study that measured S1P levels in donors undergoing G-CSF-induced mobilization (Juarez et al. 2012). Disruption of fatty acid signaling has been implicated in mobilization of stem cells, in particular S1P (Ratajczak et al. 2010). To summarize, G-CSF treatment alters fatty acid metabolism and decreases the systemic levels of fatty acid metabolites involved in hydrolysis (phospholipid metabolism and lysolipids) whereas long/medium-chain fatty acids are generally increased.

In this study we found lower levels of branched chain amino acids and aromatic amino acids following G-CSF treatment. Among the aromatic amino acids we found altered levels of tryptophan and its degradation products which are shown to be associated with inflammation. Kynurenine plays a role in modulation of inflammation and the ratio of kynurenin/tryptophan has been suggested to be an indicator for the activity of indolamine-2,3-dioxygenase (IDO) (Widner et al. 1997), which can affect T cell functions. Branched chain amino acids can be substrates for both energy production and protein synthesis. They can be metabolized to give rise to intermediates for several metabolic pathways including the TCA cycle or fatty acid synthesis. Less amounts of the branched amino acids isoleucine, valine and leucine may thus potentially have an impact on energy metabolism by reduction of available metabolites. However, in addition to the lower levels of these amino acids, we also observed a general decrease in dipeptide levels after G-CSF administration which is supportive of reduced proteolysis.

Previous studies have examined effects of in vivo administration of G-CSF on normal peripheral blood mononuclear cells (PBMCs) using whole genome expression profiling. Changes in global gene expression profiles were then described both at early time points after G-CSF administration (up to 5 days) (Hernandez et al. 2005) and after 2–10 months (Amariglio et al. 2007), and even a year after G-CSF administration in CD34^+^ progenitor cells (Baez et al. 2014). These studies have revealed that there seems to be both early responses to G-CSF, transient changes that are normalized over time and more long-lasting changes. In the recent study by Bàez et al., G-CSF-mobilized hematopoietic progenitors had a difference in the expression of six microRNAs and even one year after G-CSF administration over 2424 genes maintained their altered expression (Baez et al. 2014). Among the differentially expressed genes were genes involved in cellular growth, cell death and survival, protein synthesis, gene expression and nucleic acid metabolism. In another study of twenty stem cell donors, changes of DNA methyltransferase activity in peripheral blood cells were found after G-CSF administration, though these changes returned to baseline within a week after apheresis (Leitner et al. 2014). Thus, several studies of donor cells suggest epigenetic changes induced after G-CSF administration in healthy donors, though none of these studies have investigated the global metabolite profile of healthy donors after G-CSF treatment. Accordingly, in our study of the systemic metabolite profile, we observed altered nucleic acid metabolism by G-CSF therapy, including lower levels of the purine nucleosides. Furthermore, several methylated products were increased which could suggest a difference in methylation potential through treatment with G-CSF. These results should be interpreted with great care, but could suggest that G-CSF has the potential to influence epigenetics.

We have not performed any functional assays to evaluate the potential association between metabolites altered by G-CSF and immunomodulatory effects; however, G-CSF has been shown to induce several cellular and immunological changes in donor cells (Anderlini et al. 1996; Shaw et al. 2015), and several of the altered metabolites found in our study have known immunoregulatory and/or angioregulatory effects, or can be markers of altered regulation of epigenetic/gene expression that may contribute to the previously described long-lasting effects after G-CSF therapy (Baez et al. 2014). Altered metabolite levels may reflect the direct effects of G-CSF on different immune cell types, affecting cell proliferation, differentiation and function, but also indirect effects through e.g. upregulation of cytokine production may subsequently affect cells and lead to altered metabolite levels. G-CSF binds to the single high-affinity 140 kDa G-CSF receptor (G-CSFR), which is expressed on myeloid progenitor cells, mature granulocytes and monocytes, lymphocytes and endothelial cells (Demetri and Griffin 1991; Franzke et al. 2003) and can activate multiple signaling pathways including JAK-STAT and ERK/MAPK pathways. However, the expression of this receptor does not seem to be required for progenitor mobilization induced by G-CSF (Liu et al. 2000), and G-CSF-mediated effects may also occur independent of the G-CSF receptor. Further studies are needed to explore the mechanism inducing the metabolite changes after G-CSF treatment and their potential immunomodulatory effects and/or effects on other cell functions.

A possible explanation for our findings could be that they are secondary to an increased proliferation of immature hematopoietic cells to replace the cells that are lost from hematopoietic niches to the circulation. However, the CD34^+^ cell number and the total peripheral blood leukocyte number is controlled daily during stem cell mobilization, and the duration of this altered compartmentalization of hematopoietic cells is therefore relatively short (<24 h). Furthermore, the increased levels of immature hematopoietic cells in the peripheral blood probably represent a minor part of the overall number of nucleated bone marrow cells, and one should also emphasize that the cells have not yet been harvested at the time of sampling for metabolite analysis. However, we cannot exclude the possibility that the metabolic changes are secondary to an increased proliferation of bone marrow cells, but if so this is in our opinion most likely caused by direct G-CSF stimulated proliferation during the whole treatment period rather than being a compensatory mechanism to altered compartmentalization during the last hours before stem cell harvesting.

Several metabolites classified as xenobiotics were altered after G-CSF therapy. Many stem cell donors experience side effects during treatment (Stroncek et al. 1996), and acetaminophen is then recommended for pain relief. Some altered metabolites belonging to the xenobiotic class are a direct result of acetaminophen usage, and in our opinion some of the other metabolite changes may be associated with treatment toxicity and/or altered gastrointestinal function.

## Concluding remarks

In totum, our results show that the level of several metabolites changed after G-CSF administration, primarily there were (i) increased levels of lipids indicating altered fatty acid metabolism, (ii) increased levels of methylated nucleosides, (iii) changes associated with energy metabolism, and (iv) altered levels of amino acids, including reduced peptide levels indicating decreased proteolysis and altered levels of acetylated peptides. Long-term follow up studies have concluded that the use of G-CSF to mobilize stem cells appears to be safe (Shaw et al. 2015). Our study shows distinct differences in the metabolite profiles between healthy donors before and after G-CSF administration; however this is only a snapshot of the metabolomic profile of donors at an early time point after G-CSF administration and further studies should examine the metabolite profiles over time, using a larger set of donors, to clarify whether G-CSF also has long-term effects on metabolite profiles and/or if patients on long-term G-CSF therapy should be monitored with regard to metabolic abnormalities.

## Electronic supplementary material

Below is the link to the electronic supplementary material.

Supplementary material 1 (PDF 766 kb) Supplementary material 2 (DOCX 19 kb)
