# Supplementary material for: Granulocyte colony-stimulating factor alters the systemic metabolomic profile in healthy donors
Source: Metabolomics. 2016 Nov 28;13(1):2. doi: 10.1007/s11306-016-1139-x (PMC5126202; doi:10.1007/s11306-016-1139-x)
Supplement: Supplementary file 1 — Supplementary material 1 (PDF 766 kb) [file 11306_2016_1139_MOESM1_ESM.pdf]

**Supplementary Table 1.** Complete list of all metabolites identified.

**Comparison mean values significantly different:**
- (red) $p \le 0.05$, fold of change ≥ 1.00
- (green) $p \le 0.05$, fold of change < 1.00

**Comparison mean value difference approaching significance:**
- (pink) $0.05 < p < 0.10$, fold of change ≥ 1.00
- (light green) $0.05 < p < 0.10$, fold of change < 1.00

| Pathway Sort Order | Super Pathway | Sub Pathway | Biochemical Name | Platform | Mass | Comp ID | KEGG | HMDB | PubChem | Fold of Change, Paired t-Test: Post G-CSF / Pre G-CSF | | Paired t-Test: Post G-CSF / Pre G-CSF | | Mean Values | |
|---|---|---|---|---|---|---|---|---|---|---|---|---|---|---|---|
| | | | | | | | | | | Mean Pairs Ratio | Group Mean Ratio | *p*-value | *q*-value | Pre G-CSF | Post G-CSF |
| 1 | Amino Acid | Glycine, Serine and Threonine Metabolism | glycine | LC/MS pos | 263.14662 | 58 | C00037 | HMDB00123 | 750 | 1.03 | 1.03 | 0.5926 | 0.2889 | 0.9931 | 1.0186 |
| 2 | | | N-acetylglycine | LC/MS neg | 116.03531 | 27710 | | HMDB00532 | 10972 | 1.06 | 1.08 | 0.4135 | 0.2277 | 1.1664 | 1.2564 |
| 5 | | | dimethylglycine | LC/MS pos | 104.07061 | 5086 | C01026 | HMDB00092 | 673 | 1.05 | 1.05 | 0.2836 | 0.1777 | 1.0523 | 1.1063 |
| 6 | | | betaine | LC/MS pos | 118.08626 | 3141 | C00719 | HMDB00043 | 247 | 0.95 | 0.96 | 0.1464 | 0.1118 | 1.1138 | 1.0653 |
| 9 | | | serine | LC/MS pos | 106.04987 | 1648 | C00065 | HMDB00187 | 5951 | 0.79 | 0.79 | 0.0000 | 0.0003 | 1.1173 | 0.8779 |
| 10 | | | N-acetylserine | LC/MS polar | 146.04588 | 37076 | | HMDB02931 | 65249 | 1.63 | 1.61 | 0.0000 | 0.0000 | 0.8101 | 1.3054 |
| 14 | | | threonine | LC/MS pos | 120.06552 | 1284 | C00188 | HMDB00167 | 6288 | 0.92 | 0.90 | 0.0826 | 0.0743 | 1.0456 | 0.9420 |
| 15 | | | N-acetylthreonine | LC/MS neg | 160.06153 | 33939 | C01118 | | 152204 | 1.26 | 1.23 | 0.0184 | 0.0256 | 0.9305 | 1.1405 |
| 25 | | Alanine and Aspartate Metabolism | alanine | LC/MS pos | 90.05496 | 1126 | C00041 | HMDB00161 | 5950 | 1.13 | 1.11 | 0.0097 | 0.0169 | 0.9654 | 1.0764 |
| 26 | | | N-acetylalanine | LC/MS neg | 130.05096 | 1585 | C02847 | HMDB00766 | 88064 | 1.29 | 1.28 | 0.0000 | 0.0001 | 0.8756 | 1.1198 |
| 28 | | | aspartate | LC/MS polar | 132.03023 | 443 | C00049 | HMDB00191 | 5960 | 0.64 | 0.60 | 0.0068 | 0.0132 | 1.3009 | 0.7755 |
| 30 | | | asparagine | LC/MS pos | 133.06077 | 512 | C00152 | HMDB00168 | 6267 | 0.94 | 0.93 | 0.0258 | 0.0310 | 1.0885 | 1.0128 |
| 32 | | | N-acetylaspartate (NAA) | LC/MS polar | 174.04079 | 22185 | C01042 | HMDB00812 | 65065 | 0.84 | 0.84 | 0.0667 | 0.0654 | 1.0369 | 0.8707 |
| 38 | | Glutamate Metabolism | glutamate | LC/MS pos | 148.06044 | 57 | C00025 | HMDB00148 | 611 | 0.91 | 0.82 | 0.1030 | 0.0870 | 1.1249 | 0.9274 |
| 39 | | | glutamine | LC/MS pos | 147.07642 | 53 | C00064 | HMDB00641 | 5961 | 1.07 | 1.06 | 0.0387 | 0.0423 | 0.9626 | 1.0249 |
| 40 | | | N-acetylglutamate | LC/MS polar | 188.05644 | 15720 | C00624 | HMDB01138 | 70914 | 0.91 | 0.85 | 0.1624 | 0.1207 | 1.0550 | 0.8985 |
| 41 | | | N-acetylglutamine | LC/MS pos | 189.08699 | 33943 | C02716 | HMDB06029 | 182230 | 1.07 | 1.02 | 0.8441 | 0.3561 | 1.0729 | 1.0923 |
| 42 | | | N-acetyl-aspartyl-glutamate (NAAG) | LC/MS pos | 305.09795 | 35665 | C12270 | HMDB01067 | 5255 | 1.11 | 1.06 | 0.5458 | 0.2767 | 0.9585 | 1.0134 |
| 49 | | | pyroglutamine* | LC/MS pos | 129.06586 | 46225 | | | 134508 | 1.01 | 1.02 | 0.9421 | 0.3821 | 1.6535 | 1.6785 |
| 53 | | Histidine Metabolism | histidine | LC/MS neg | 154.0622 | 59 | C00135 | HMDB00177 | 6274 | 0.84 | 0.82 | 0.0013 | 0.0042 | 1.0801 | 0.8873 |
| 54 | | | N-acetylhistidine | LC/MS neg | 196.07276 | 33946 | C02997 | HMDB32055 | 75619 | 0.78 | 0.81 | 0.0212 | 0.0283 | 1.0851 | 0.8799 |
| 55 | | | 1-methylhistidine | LC/MS pos | 170.09241 | 30460 | C01152 | HMDB00001 | 92105 | 1.21 | 1.08 | 0.4340 | 0.2346 | 1.0495 | 1.1377 |
| 56 | | | 3-methylhistidine | LC/MS neg | 168.07785 | 15677 | C01152 | HMDB00479 | 64969 | 1.58 | 0.82 | 0.8074 | 0.3470 | 1.2446 | 1.0239 |
| 57 | | | N-acetyl-3-methylhistidine* | LC/MS neg | 210.08841 | 43256 | | | 193270 | 2.45 | 1.49 | 0.6148 | 0.2952 | 0.8506 | 1.2711 |
| 58 | | | N-acetyl-1-methylhistidine* | LC/MS pos | 212.1028 | 43255 | | | | 1.02 | 1.03 | 0.6978 | 0.3210 | 1.2117 | 1.2509 |
| 59 | | | hydantoin-5-propionic acid | LC/MS polar | 171.04113 | 40473 | C05565 | HMDB01212 | 782 | 1.16 | 1.10 | 0.6077 | 0.2924 | 1.2455 | 1.3746 |
| 60 | | | trans-urocanate | LC/MS pos | 139.05021 | 607 | C00785 | HMDB00301 | 736715 | 1.58 | 1.40 | 0.9219 | 0.3770 | 1.1521 | 1.6166 |
| 61 | | | cis-urocanate | LC/MS pos | 139.05021 | 40410 | | | 1549103 | 2.21 | 2.26 | 0.7392 | 0.3303 | 1.1927 | 2.6965 |
| 63 | | | imidazole propionate | LC/MS pos | 141.06586 | 40730 | | HMDB02271 | 70630 | 0.87 | 0.90 | 0.0344 | 0.0388 | 1.4112 | 1.2745 |
| 64 | | | imidazole lactate | LC/MS pos | 157.06077 | 15716 | C05568 | HMDB02320 | 440129 | 1.06 | 1.00 | 0.7503 | 0.3314 | 0.9544 | 0.9559 |
| 70 | | | 1-methylimidazoleacetate | LC/MS pos | 141.06586 | 32350 | C05828 | HMDB02820 | 75810 | 1.95 | 1.06 | 0.2762 | 0.1754 | 0.9766 | 1.0325 |
| 71 | | | 4-imidazoleacetate | LC/MS polar | 125.03565 | 32349 | C02835 | HMDB02024 | 96215 | 0.67 | 0.67 | 0.0022 | 0.0062 | 1.0848 | 0.7269 |
| 72 | | | N-acetylhistamine | LC/MS pos | 154.09749 | 48679 | C05135 | HMDB13253 | 69602 | 0.97 | 0.52 | 0.2202 | 0.1488 | 1.3101 | 0.6866 |
| 73 | | Lysine Metabolism | lysine | LC/MS pos | 147.11281 | 1301 | C00047 | HMDB00182 | 5962 | 0.83 | 0.82 | 0.0002 | 0.0009 | 1.1419 | 0.9333 |
| 74 | | | N2-acetyllysine | LC/MS pos | 189.12337 | 36751 | C12989 | HMDB00446 | 92907 | 0.80 | 0.72 | 0.0133 | 0.0213 | 1.2041 | 0.8660 |
| 75 | | | N6-acetyllysine | LC/MS pos | 189.12337 | 36752 | C02727 | HMDB00206 | 92832 | 1.15 | 1.14 | 0.0030 | 0.0074 | 0.9215 | 1.0495 |
| 76 | | | N-6-trimethyllysine | LC/MS pos | 189.15976 | 1498 | C03793 | HMDB01325 | 440120 | 1.04 | 0.98 | 0.9011 | 0.3708 | 1.0179 | 0.9984 |
| 79 | | | 2-aminoadipate | LC/MS pos | 162.07609 | 6146 | C00956 | HMDB00510 | 469 | 0.83 | 0.75 | 0.0157 | 0.0234 | 1.3280 | 0.9993 |
| 82 | | | glutarate (pentanedioate) | LC/MS polar | 131.03498 | 396 | C00489 | HMDB00661 | 743 | 0.66 | 0.62 | 0.0023 | 0.0064 | 1.7006 | 1.0553 |
| 83 | | | glutarylcarnitine (C5) | LC/MS pos | 276.14417 | 44664 | | HMDB13130 | 71464488 | 1.01 | 1.00 | 0.9426 | 0.3821 | 1.0277 | 1.0318 |
| 84 | | | 3-methylglutarylcarnitine (1) | LC/MS pos | 290.15982 | 46547 | | HMDB00552 | 128145 | 2.23 | 2.19 | 0.0000 | 0.0000 | 0.9545 | 2.0915 |
| 85 | | | 3-methylglutarylcarnitine (2) | LC/MS pos | 290.15982 | 46548 | | HMDB00552 | 128145 | 1.03 | 0.87 | 0.6702 | 0.3123 | 1.3498 | 1.1801 |
| 90 | | | pipecolate | LC/MS pos | 130.08626 | 1444 | C00408 | HMDB00070 | 849 | 0.98 | 0.97 | 0.3525 | 0.2017 | 1.1748 | 1.1358 |
| 98 | | Phenylalanine and Tyrosine Metabolism | phenylalanine | LC/MS pos | 166.08626 | 64 | C00079 | HMDB00159 | 6140 | 0.88 | 0.87 | 0.0019 | 0.0057 | 1.0679 | 0.9320 |
| 99 | | | N-acetylphenylalanine | LC/MS neg | 206.08226 | 33950 | C03519 | HMDB00512 | 74839 | 0.97 | 0.96 | 0.3551 | 0.2028 | 1.1306 | 1.0799 |
| 102 | | | phenylpyruvate | LC/MS neg | 163.04007 | 566 | C00166 | HMDB00205 | 997 | 0.94 | 0.92 | 0.1332 | 0.1045 | 1.1377 | 1.0462 |
| 103 | | | phenyllactate (PLA) | LC/MS neg | 165.05571 | 22130 | C05607 | HMDB00779 | 3848 | 1.10 | 1.03 | 0.5923 | 0.2889 | 1.2251 | 1.2564 |
| 106 | | | phenylacetate | LC/MS neg | 135.04515 | 15958 | C07086 | HMDB00209 | 999 | 0.78 | 0.71 | 0.0179 | 0.0251 | 1.3119 | 0.9367 |
| 107 | | | 4-hydroxyphenylacetate | LC/MS neg | 151.04007 | 541 | C00642 | HMDB00020 | 127 | 0.91 | 0.84 | 0.1092 | 0.0910 | 1.3172 | 1.1110 |
| 110 | | | phenylacetylglutamine | LC/MS pos | 265.11829 | 35126 | C04148 | HMDB06344 | 92258 | 1.02 | 0.81 | 0.3858 | 0.2164 | 1.3564 | 1.0965 |
| 111 | | | tyrosine | LC/MS pos | 182.08117 | 1299 | C00082 | HMDB00158 | 6057 | 0.78 | 0.77 | 0.0000 | 0.0001 | 1.1540 | 0.8850 |
| 112 | | | N-acetyltyrosine | LC/MS neg | 222.07718 | 32390 | | HMDB00866 | 68310 | 0.72 | 0.73 | 0.0002 | 0.0013 | 1.1960 | 0.8752 |
| 120 | | | 4-hydroxyphenylpyruvate | LC/MS neg | 179.03498 | 1669 | C01179 | HMDB00707 | 979 | 0.99 | 0.93 | 0.4110 | 0.2270 | 0.9760 | 0.9054 |
| 121 | | | 3-(4-hydroxyphenyl)lactate | LC/MS neg | 181.05063 | 32197 | C03672 | HMDB00755 | 9378 | 1.00 | 0.96 | 0.6507 | 0.3072 | 1.1211 | 1.0818 |
| 126 | | | phenol sulfate | LC/MS neg | 172.9914 | 32553 | C02180 | HMDB60015 | 74426 | 0.98 | 0.92 | 0.3907 | 0.2186 | 2.1406 | 1.9601 |
| 128 | | | p-cresol sulfate | LC/MS neg | 187.00705 | 36103 | C01468 | HMDB11635 | 4615423 | 1.84 | 0.88 | 0.4789 | 0.2524 | 1.2436 | 1.0977 |
| 134 | | | o-cresol sulfate | LC/MS neg | 187.00705 | 36845 | | | 11615528 | 1.53 | 1.42 | 0.0629 | 0.0628 | 0.9383 | 1.3313 |
| 139 | | | vanillylmandelate (VMA) | LC/MS neg | 197.04555 | 1567 | C05584 | HMDB00291 | 1245 | 0.93 | 0.94 | 0.1767 | 0.1280 | 1.0534 | 0.9886 |
| 141 | | | 3-methoxytyrosine | LC/MS pos | 212.09174 | 12017 | | HMDB01434 | 1670 | 1.11 | 1.11 | 0.0027 | 0.0072 | 1.0033 | 1.1138 |
| 143 | | | 3-methoxytyramine sulfate | LC/MS neg | 246.04416 | 44618 | | | | 0.93 | 0.67 | 0.1658 | 0.1222 | 1.0819 | 0.7269 |
| 148 | | | homovanillate (HVA) | LC/MS neg | 181.05063 | 1101 | C05582 | HMDB00118 | 1738 | 0.76 | 0.77 | 0.0062 | 0.0125 | 1.1547 | 0.8859 |
| 163 | | | gentisate | LC/MS polar | 153.01933 | 18280 | C00628 | HMDB00152 | 3469 | 0.96 | 0.74 | 0.1138 | 0.0937 | 1.2615 | 0.9341 |
| 167 | | | 3-[3-(sulfooxy)phenyl]propanoic acid | LC/MS neg | 245.01253 | 45415 | | | 187488 | 1.47 | 0.49 | 0.1331 | 0.1045 | 1.8998 | 0.9335 |
| 171 | | | 3-(3-hydroxyphenyl)propionate | LC/MS neg | 165.05571 | 35635 | C11457 | HMDB00375 | 91 | 2.84 | 0.70 | 0.1546 | 0.1167 | 1.2430 | 0.8666 |
| 172 | | | 3-(4-hydroxyphenyl)propionate | LC/MS neg | 165.05571 | 39587 | C01744 | HMDB02199 | 10394 | 0.94 | 0.78 | 0.1292 | 0.1024 | 0.8551 | 0.6685 |
| 174 | | | 3-phenylpropionate (hydrocinnamate) | LC/MS neg | 149.0608 | 15749 | C05629 | HMDB00764 | 107 | 0.81 | 0.67 | 0.0326 | 0.0373 | 2.1967 | 1.4678 |
| 179 | | | thyroxine | LC/MS neg | 775.67944 | 2761 | C01829 | HMDB01918 | 5819 | 0.95 | 0.94 | 0.0296 | 0.0344 | 1.0667 | 1.0056 |
| 180 | | | 5-hydroxymethyl-2-furoic acid | LC/MS neg | 141.01933 | 42040 | C20448 | HMDB02432 | 80642 | 0.82 | 0.54 | 0.0227 | 0.0289 | 1.6842 | 0.9148 |
| 181 | | | 2-hydroxyphenylacetate | LC/MS neg | 151.04006 | 1432 | C05852 | HMDB00669 | 11970 | 0.94 | 0.86 | 0.2415 | 0.1590 | 1.2094 | 1.0369 |
| 183 | | | phenylacetylcarnitine | LC/MS pos | 280.15434 | 48425 | | | | 1.01 | 0.85 | 0.1935 | 0.1370 | 1.1813 | 1.0027 |
| 184 | | | dopamine sulfate (1) | LC/MS neg | 232.02848 | 48406 | | | | 1.06 | 0.75 | 0.5368 | 0.2733 | 1.6253 | 1.2190 |
| 185 | | | dopamine sulfate (2) | LC/MS neg | 232.02845 | 48407 | | | | 1.02 | 0.75 | 0.4566 | 0.2432 | 1.4719 | 1.1076 |
| 186 | | | p-cresol-glucuronide* | LC/MS neg | 283.08232 | 48841 | | HMDB11686 | 154035 | 3.75 | 0.99 | 0.2788 | 0.1758 | 1.1125 | 1.0986 |
| 187 | | | tyramine O-sulfate | LC/MS neg | 216.0336 | 48408 | | HMDB06409 | 153005 | 3.42 | 0.86 | 0.5074 | 0.2627 | 2.1093 | 1.8182 |
| 188 | | | N-formylphenylalanine | LC/MS neg | 192.06661 | 48433 | | | 759256 | 0.78 | 0.61 | 0.0178 | 0.0251 | 1.2909 | 0.7914 |
| 193 | | Tryptophan Metabolism | tryptophan | LC/MS pos | 205.09716 | 54 | C00078 | HMDB00929 | 6305 | 0.75 | 0.75 | 0.0000 | 0.0000 | 1.1807 | 0.8829 |
| 194 | | | N-acetyltryptophan | LC/MS neg | 245.09316 | 33959 | C03137 | HMDB13713 | 700653 | 0.75 | 0.73 | 0.0016 | 0.0050 | 1.2745 | 0.9310 |
| 197 | | | indolelactate | LC/MS pos | 206.08117 | 18349 | C02043 | HMDB00671 | 92904 | 0.94 | 0.92 | 0.1440 | 0.1107 | 1.2943 | 1.1847 |
| 198 | | | indoleacetate | LC/MS pos | 176.07061 | 27513 | C00954 | HMDB00197 | 802 | 0.86 | 0.82 | 0.0250 | 0.0301 | 1.4667 | 1.1978 |
| 200 | | | indolepropionate | LC/MS pos | 190.08626 | 32405 | | HMDB02302 | 3744 | 0.92 | 0.78 | 0.1525 | 0.1157 | 1.2646 | 0.9878 |
| 202 | | | 3-indoxyl sulfate | LC/MS neg | 212.0023 | 27672 | | HMDB00682 | 10258 | 0.91 | 0.90 | 0.0814 | 0.0737 | 1.1600 | 1.0452 |
| 203 | | | kynurenine | LC/MS pos | 209.09207 | 15140 | C00328 | HMDB00684 | 161166 | 0.84 | 0.83 | 0.0004 | 0.0020 | 1.1147 | 0.9269 |
| 204 | | | kynurenate | LC/MS neg | 188.03531 | 1417 | C01717 | HMDB00715 | 3845 | 0.77 | 0.77 | 0.0007 | 0.0027 | 1.2853 | 0.9840 |
| 211 | | | picolinate | LC/MS pos | 124.03931 | 1512 | C10164 | HMDB02243 | 1018 | 0.62 | 0.60 | 0.0000 | 0.0001 | 1.3651 | 0.8190 |
| 218 | | | 5-hydroxyindoleacetate | LC/MS pos | 190.05096 | 437 | C05635 | HMDB00763 | 1826 | 0.63 | 0.49 | 0.0033 | 0.0078 | 1.5830 | 0.7758 |
| 219 | | | serotonin | LC/MS pos | 177.10224 | 2342 | C00780 | HMDB00259 | 5202 | 0.76 | 0.69 | 0.0911 | 0.0796 | 1.3318 | 0.9224 |
| 224 | | | indolebutyrate | LC/MS pos | 204.10191 | 34402 | C11284 | HMDB02096 | 8617 | 1.07 | 1.08 | 0.5051 | 0.2627 | 0.7080 | 0.7641 |
| 226 | | | indoleacetylglutamine | LC/MS pos | 302.11463 | 42087 | | HMDB13240 | 25200879 | 1.00 | 0.92 | 0.4150 | 0.2277 | 1.3199 | 1.2157 |
| 227 | | | tryptophan betaine | LC/MS pos | 247.14411 | 37097 | C09213 | HMDB61115 | 442106 | 0.55 | 0.60 | 0.0000 | 0.0000 | 3.6167 | 2.1754 |
| 233 | | | indole-3-carboxylic acid | LC/MS neg | 160.0404 | 38116 | C19837 | HMDB03320 | 69867 | 1.15 | 1.03 | 0.6661 | 0.3114 | 1.0085 | 1.0392 |
| 234 | | | C-glycosyltryptophan | LC/MS pos | 367.14998 | 48782 | | | 10981970 | 1.10 | 1.09 | 0.1094 | 0.0910 | 0.9702 | 1.0534 |
| 235 | | | N-acetylkynurenine (2) | LC/MS neg | 249.08808 | 48757 | | | | 0.68 | 0.60 | 0.0010 | 0.0035 | 1.1806 | 0.7137 |
| 237 | | Leucine, Isoleucine and Valine Metabolism | leucine | LC/MS pos | 132.10191 | 60 | C00123 | HMDB00687 | 6106 | 0.81 | 0.80 | 0.0002 | 0.0012 | 1.1029 | 0.8828 |
| 238 | | | N-acetylleucine | LC/MS neg | 172.09791 | 1587 | C02710 | HMDB11756 | 70912 | 0.83 | 0.83 | 0.0153 | 0.0234 | 1.0607 | 0.8766 |
| 240 | | | 4-methyl-2-oxopentanoate | LC/MS neg | 129.05571 | 22116 | C00233 | HMDB00695 | 70 | 0.86 | 0.86 | 0.0157 | 0.0234 | 1.1075 | 0.9495 |
| 243 | | | isovalerate | LC/MS neg | 101.0608 | 44656 | C08262 | HMDB00718 | 10430 | 0.60 | 0.58 | 0.0000 | 0.0001 | 1.2311 | 0.7200 |
| 244 | | | isovalerylglycine | LC/MS neg | 158.08226 | 35107 | | HMDB00678 | 546304 | 0.54 | 0.55 | 0.0008 | 0.0030 | 1.2632 | 0.6927 |
| 245 | | | isovalerylcarnitine | LC/MS pos | 246.16999 | 34407 | | HMDB00688 | 6426851 | 0.74 | 0.74 | 0.0005 | 0.0022 | 1.1596 | 0.8548 |
| 248 | | | beta-hydroxyisovalerate | LC/MS neg | 117.05572 | 12129 | | HMDB00754 | 69362 | 0.83 | 0.82 | 0.0012 | 0.0041 | 1.1749 | 0.9671 |
| 249 | | | beta-hydroxyisovaleroylcarnitine | LC/MS pos | 262.1649 | 35433 | | | | 0.88 | 0.88 | 0.0444 | 0.0477 | 1.1032 | 0.9744 |
| 252 | | | 3-methylglutaconate | LC/MS polar | 143.03498 | 38667 | | HMDB00522 | 1551553 | 0.94 | 0.96 | 0.2008 | 0.1398 | 1.1269 | 1.0768 |
| 256 | | | alpha-hydroxyisovalerate | LC/MS neg | 117.05571 | 33937 | | HMDB00407 | 99823 | 1.21 | 1.27 | 0.0071 | 0.0134 | 1.0033 | 1.2732 |
| 258 | | | methylsuccinate | LC/MS polar | 131.03498 | 15745 | | HMDB01844 | 10349 | 0.93 | 0.92 | 0.1095 | 0.0910 | 1.0555 | 0.9691 |
| 266 | | | isoleucine | LC/MS pos | 132.10191 | 1125 | C00407 | HMDB00172 | 6306 | 0.80 | 0.78 | 0.0005 | 0.0024 | 1.1221 | 0.8719 |
| 267 | | | allo-isoleucine | GC/MS | 274.22357 | 47034 | | | 6950182;99288 | 1.31 | 1.25 | 0.0085 | 0.0155 | 0.9935 | 1.2390 |
| 268 | | | N-acetylisoleucine | LC/MS neg | 172.09791 | 33967 | | | 2802421 | 0.95 | 0.93 | 0.2237 | 0.1504 | 0.9721 | 0.9087 |
| 269 | | | 3-methyl-2-oxovalerate | LC/MS neg | 129.05572 | 15676 | C00671 | HMDB03736 | 47 | 0.86 | 0.86 | 0.0105 | 0.0176 | 1.1373 | 0.9769 |
| 270 | | | 2-methylbutyrylcarnitine (C5) | LC/MS pos | 246.16999 | 45095 | | HMDB00378 | 6426901 | 0.89 | 0.86 | 0.0219 | 0.0285 | 1.0657 | 0.9210 |
| 272 | | | tiglyl carnitine | LC/MS pos | 244.15434 | 35428 | | HMDB02366 | 22833596 | 1.03 | 0.99 | 0.9726 | 0.3895 | 1.0775 | 1.0638 |
| 273 | | | tigloylglycine | LC/MS pos | 158.08117 | 1598 | | HMDB00959 | 6441567 | 0.61 | 0.57 | 0.0001 | 0.0006 | 1.2313 | 0.6963 |
| 275 | | | 2-hydroxy-3-methylvalerate | LC/MS neg | 131.07136 | 36746 | | HMDB00317 | 164623 | 1.20 | 1.07 | 0.4459 | 0.2385 | 0.9196 | 0.9816 |
| 276 | | | 3-hydroxy-2-ethylpropionate | LC/MS neg | 117.05571 | 32397 | | HMDB00396 | 188979 | 1.02 | 1.01 | 0.6731 | 0.3127 | 1.0507 | 1.0572 |
| 277 | | | ethylmalonate | LC/MS polar | 131.03498 | 15765 | | HMDB00622 | 11756 | 1.26 | 1.24 | 0.0047 | 0.0099 | 1.1462 | 1.4161 |
| 278 | | | valine | LC/MS pos | 118.08626 | 1649 | C00183 | HMDB00883 | 6287 | 0.81 | 0.80 | 0.0000 | 0.0001 | 1.0966 | 0.8766 |
| 279 | | | N-acetylvaline | LC/MS neg | 158.08226 | 1591 | | HMDB11757 | 66789 | 1.09 | 1.08 | 0.0175 | 0.0248 | 0.9730 | 1.0539 |
| 281 | | | 3-methyl-2-oxobutyrate | LC/MS neg | 115.04006 | 44526 | C00141 | HMDB00019 | 49 | 1.07 | 1.06 | 0.5078 | 0.2627 | 1.0511 | 1.1160 |
| 282 | | | isobutyrylcarnitine | LC/MS pos | 232.15434 | 33441 | | HMDB00736 | 168379 | 0.82 | 0.78 | 0.0056 | 0.0116 | 1.2666 | 0.9868 |
| 283 | | | isobutyrylglycine | LC/MS neg | 144.06661 | 35437 | | HMDB00730 | 10855600 | 0.57 | 0.55 | 0.0001 | 0.0004 | 1.4584 | 0.8001 |
| 284 | | | 3-hydroxyisobutyrate | LC/MS polar | 103.04007 | 1549 | C06001 | HMDB00336 | 87 | 0.86 | 0.81 | 0.0243 | 0.0299 | 1.1686 | 0.9447 |
| 285 | | | alpha-hydroxyisocaproate | LC/MS neg | 131.07136 | 22132 | C03264 | HMDB00746 | 83697 | 0.95 | 0.93 | 0.2323 | 0.1552 | 0.9515 | 0.8831 |
| 290 | | Methionine, Cysteine, SAM and Taurine Metabolism | methionine | LC/MS pos | 150.05833 | 1302 | C00073 | HMDB00696 | 6137 | 0.77 | 0.75 | 0.0001 | 0.0005 | 1.1628 | 0.8752 |
| 291 | | | N-acetylmethionine | LC/MS neg | 190.05434 | 1589 | C02712 | HMDB11745 | 448580 | 1.89 | 1.88 | 0.0000 | 0.0000 | 0.7976 | 1.4971 |
| 292 | | | N-formylmethionine | LC/MS neg | 176.03869 | 2829 | C03145 | HMDB01015 | 439750 | 1.05 | 1.06 | 0.5808 | 0.2862 | 1.0324 | 1.0930 |
| 294 | | | methionine sulfone | LC/MS polar | 180.0336 | 44878 | | | 69961 | 1.04 | 1.04 | 0.6819 | 0.3158 | 1.0787 | 1.1183 |
| 295 | | | methionine sulfoxide | LC/MS polar | 164.03869 | 18374 | C02989 | HMDB02005 | 158980 | 0.94 | 0.90 | 0.2070 | 0.1418 | 1.0534 | 0.9517 |
| 298 | | | S-adenosylhomocysteine (SAH) | LC/MS neg | 383.11431 | 42382 | C00021 | HMDB00939 | 439155 | 1.33 | 1.18 | 0.4639 | 0.2465 | 0.7005 | 0.8258 |
| 303 | | | alpha-ketobutyrate | LC/MS polar | 101.02442 | 4968 | C00109 | HMDB00005 | 58 | 1.75 | 1.48 | 0.0300 | 0.0347 | 0.9656 | 1.4328 |
| 305 | | | 2-aminobutyrate | LC/MS pos | 104.07061 | 42374 | C02261 | HMDB00650 | 439691 | 1.01 | 0.99 | 0.8186 | 0.3494 | 1.0219 | 1.0119 |
| 306 | | | 2-hydroxybutyrate (AHB) | LC/MS polar | 103.04006 | 21044 | C05984 | HMDB00008 | 440864 | 1.41 | 1.30 | 0.0170 | 0.0245 | 0.9659 | 1.2542 |
| 307 | | | cysteine | GC/MS | 218 | 31453 | C00097 | HMDB00574 | 5862 | 0.69 | 0.66 | 0.0005 | 0.0022 | 1.2676 | 0.8375 |
| 309 | | | cystine | LC/MS pos | 241.03113 | 56 | C00491 | HMDB00192 | 67678 | 0.67 | 0.65 | 0.0000 | 0.0000 | 1.2565 | 0.8189 |
| 310 | | | S-methylcysteine | LC/MS neg | 134.02812 | 39592 | | HMDB02108 | 24417 | 1.03 | 0.93 | 0.5986 | 0.2896 | 1.1396 | 1.0628 |
| 312 | | | cysteine s-sulfate | LC/MS neg | 199.96928 | 22176 | C05824 | HMDB00731 | 115015 | 0.69 | 0.54 | 0.0030 | 0.0074 | 1.2694 | 0.6871 |
| 316 | | | hypotaurine | LC/MS polar | 108.01247 | 590 | C00519 | HMDB00965 | 107812 | 1.04 | 0.91 | 0.6069 | 0.2924 | 1.2855 | 1.1651 |
| 317 | | | taurine | LC/MS neg | 124.00739 | 2125 | C00245 | HMDB00251 | 1123 | 0.78 | 0.73 | 0.0077 | 0.0144 | 1.2349 | 0.9064 |
| 318 | | | N-acetyltaurine | LC/MS neg | 166.01795 | 48187 | | | 159864 | 1.62 | 1.60 | 0.0000 | 0.0001 | 0.9214 | 1.4727 |
| 325 | | Urea cycle; Arginine and Proline Metabolism | arginine | LC/MS pos | 175.11896 | 1638 | C00062 | HMDB00517 | 232 | 0.85 | 0.84 | 0.0031 | 0.0075 | 1.1366 | 0.9536 |
| 326 | | | urea | LC/MS pos | 121.07201 | 1670 | C00086 | HMDB00294 | 1176 | 0.84 | 0.82 | 0.0040 | 0.0087 | 1.1318 | 0.9297 |
| 327 | | | ornithine | LC/MS polar | 131.0826 | 1493 | C00077 | HMDB03374 | 6262 | 0.68 | 0.67 | 0.0001 | 0.0007 | 1.2163 | 0.8201 |
| 328 | | | proline | LC/MS pos | 116.07061 | 1898 | C00148 | HMDB00162 | 145742 | 1.01 | 1.00 | 0.9388 | 0.3821 | 1.0226 | 1.0219 |
| 329 | | | citrulline | LC/MS pos | 176.10297 | 2132 | C00327 | HMDB00904 | 9750 | 0.94 | 0.92 | 0.1759 | 0.1280 | 1.0654 | 0.9769 |
| 332 | | | homocitrulline | LC/MS pos | 190.11862 | 22138 | C02427 | HMDB00679 | 65072 | 1.31 | 1.26 | 0.0570 | 0.0585 | 1.0883 | 1.3693 |
| 336 | | | dimethylarginine (SDMA + ADMA) | LC/MS pos | 203.15026 | 36808 | C03626 | HMDB01539 | 123831 | 1.26 | 1.25 | 0.0001 | 0.0004 | 0.8930 | 1.1130 |
| 337 | | | N-acetylarginine | LC/MS pos | 217.12952 | 33953 | C02562 | HMDB04620 | 67427 | 0.65 | 0.66 | 0.0000 | 0.0000 | 1.4152 | 0.9292 |
| 340 | | | N-delta-acetylornithine | LC/MS pos | 175.10772 | 43249 | | | 9920500 | 0.91 | 0.91 | 0.0953 | 0.0824 | 1.0595 | 0.9639 |
| 344 | | | N-methylproline | LC/MS pos | 130.08626 | 37431 | | | 557 | 1.36 | 0.83 | 0.5715 | 0.2847 | 2.3253 | 1.9318 |
| 346 | | | trans-4-hydroxyproline | LC/MS pos | 132.06552 | 32306 | C01157 | HMDB00725 | 5810 | 1.04 | 0.94 | 0.7143 | 0.3239 | 1.1325 | 1.0696 |
| 348 | | | pro-hydroxy-pro | LC/MS pos | 229.11829 | 35127 | | HMDB06695 | 11673055 | 1.07 | 1.06 | 0.5858 | 0.2872 | 1.0023 | 1.0618 |
| 352 | | | N-acetylcitrulline | LC/MS neg | 216.09898 | 48434 | C15532 | HMDB00856 | 656979 | 0.59 | 0.60 | 0.0005 | 0.0022 | 1.9575 | 1.1697 |
| 353 | | Creatine Metabolism | creatine | LC/MS pos | 132.07676 | 27718 | C00300 | HMDB00064 | 586 | 0.98 | 0.97 | 0.4960 | 0.2587 | 1.0013 | 0.9676 |
| 354 | | | creatinine | LC/MS pos | 114.06619 | 513 | C00791 | HMDB00562 | 588 | 1.08 | 1.07 | 0.0697 | 0.0667 | 0.9715 | 1.0420 |
| 359 | | | guanidinoacetate | LC/MS polar | 116.04655 | 43802 | C00581 | HMDB00128 | 763 | 0.72 | 0.71 | 0.0000 | 0.0002 | 1.1968 | 0.8516 |
| 362 | | Polyamine Metabolism | acisoga | LC/MS pos | 185.12846 | 43258 | | | 129397 | 1.33 | 1.25 | 0.0060 | 0.0121 | 0.8772 | 1.1006 |
| 366 | | | 5-methylthioadenosine (MTA) | LC/MS neg | 296.0823 | 1419 | C00170 | HMDB01173 | 439176 | 1.19 | 1.18 | 0.0012 | 0.0041 | 0.9092 | 1.0733 |
| 369 | | | N-acetylputrescine | LC/MS pos | 131.11789 | 37496 | C02714 | HMDB02064 | 122356 | 1.62 | 1.05 | 0.5801 | 0.2862 | 0.9360 | 0.9811 |
| 370 | | | 4-acetamidobutanoate | LC/MS pos | 146.08117 | 1558 | C02946 | HMDB03681 | 18189 | 0.95 | 0.95 | 0.2156 | 0.1462 | 1.0295 | 0.9786 |
| 375 | | Guanidino and Acetamido Metabolism | 4-guanidinobutanoate | LC/MS pos | 146.09241 | 15681 | C01035 | HMDB03464 | 500 | 0.93 | 0.92 | 0.1895 | 0.1346 | 1.1762 | 1.0778 |
| 376 | | | guanidinosuccinate | LC/MS polar | 174.05203 | 32446 | C03139 | HMDB03157 | 97856 | 1.06 | 1.09 | 0.2840 | 0.1777 | 0.6731 | 0.7356 |
| 380 | | Glutathione Metabolism | cysteine-glutathione disulfide | LC/MS pos | 214.05123 | 35159 | | HMDB00656 | 4247235 | 0.95 | 0.89 | 0.2246 | 0.1506 | 0.9960 | 0.8907 |
| 384 | | | cys-gly, oxidized | LC/MS neg | 353.0595 | 18368 | | | 333293 | 1.16 | 1.15 | 0.1324 | 0.1045 | 1.0214 | 1.1729 |
| 385 | | | 5-oxoproline | LC/MS neg | 128.03531 | 1494 | C01879 | HMDB00267 | 7405 | 0.94 | 0.93 | 0.0930 | 0.0808 | 1.0355 | 0.9641 |
| 393 | | Gamma-glutamyl Amino Acid | gamma-glutamylalanine | LC/MS pos | 219.09755 | 37063 | | HMDB29142 | 440103 | 1.01 | 1.00 | 0.8125 | 0.3473 | 1.0106 | 1.0076 |
| 395 | | | gamma-glutamylglutamate | LC/MS pos | 277.10303 | 36738 | C05282 | HMDB11737 | 92865 | 0.80 | 0.77 | 0.0367 | 0.0406 | 1.2617 | 0.9684 |
| 396 | | | gamma-glutamylglutamine | LC/MS pos | 276.11902 | 2730 | C05283 | HMDB11738 | 150914 | 0.92 | 0.91 | 0.0717 | 0.0680 | 1.0731 | 0.9790 |
| 397 | | | gamma-glutamylglycine | LC/MS neg | 203.06734 | 33949 | | HMDB11667 | 165527 | 0.67 | 0.50 | 0.0068 | 0.0132 | 1.7954 | 0.9043 |
| 398 | | | gamma-glutamylhistidine | LC/MS neg | 283.10479 | 18245 | | | 7017195 | 0.90 | 0.87 | 0.0921 | 0.0802 | 1.3621 | 1.1883 |
| 399 | | | gamma-glutamylisoleucine* | LC/MS pos | 261.1445 | 34456 | | HMDB11170 | 14253342 | 0.87 | 0.81 | 0.0342 | 0.0387 | 1.1889 | 0.9599 |
| 400 | | | gamma-glutamylleucine | LC/MS pos | 261.1445 | 18369 | | HMDB11171 | 151023 | 0.86 | 0.83 | 0.0132 | 0.0213 | 1.1059 | 0.9147 |
| 401 | | | gamma-glutamyl-epsilon-lysine | LC/MS pos | 276.1554 | 33934 | | HMDB03869 | 65254;14284565 | 0.98 | 0.93 | 0.4439 | 0.2384 | 1.0674 | 0.9955 |
| 402 | | | gamma-glutamylmethionine | LC/MS neg | 277.08636 | 44872 | | HMDB29155 | 7009567 | 0.78 | 0.74 | 0.0029 | 0.0073 | 1.2240 | 0.9080 |

**Comparison mean values significantly different:**

- (red) $p \leq 0.05$, fold of change ≥ 1.00
- (dark green) $p \leq 0.05$, fold of change < 1.00

**Comparison mean value difference approaching significance:**

- (pink) $0.05 < p < 0.10$, fold of change ≥ 1.00
- (light green) $0.05 < p < 0.10$, fold of change < 1.00

| Pathway Sort Order | Super Pathway | Sub Pathway | Biochemical Name | Platform | Mass | Comp ID | KEGG | HMDB | PubChem | Fold of Change, Paired t-Test: Post G-CSF / Pre G-CSF — Mean Pairs Ratio | Fold of Change, Paired t-Test: Post G-CSF / Pre G-CSF — Group Mean Ratio | Paired t-Test: Post G-CSF / Pre G-CSF — *p*-value | Paired t-Test: Post G-CSF / Pre G-CSF — *q*-value | Mean Values — Pre G-CSF | Mean Values — Post G-CSF |
|---|---|---|---|---|---|---|---|---|---|---|---|---|---|---|---|
| 403 | Peptide | | gamma-glutamylphenylalanine | LC/MS pos | 295.12885 | 33422 | | HMDB00594 | 111299 | 0.98 | 0.96 | 0.4448 | 0.2384 | 1.0615 | 1.0153 |
| 404 | | | gamma-glutamylthreonine* | LC/MS pos | 249.10812 | 33364 | | HMDB29159 | | 0.90 | 0.90 | 0.0482 | 0.0511 | 1.0908 | 0.9776 |
| 405 | | | gamma-glutamyltryptophan | LC/MS pos | 334.13975 | 33947 | | HMDB29160 | 3989307 | 0.80 | 0.78 | 0.0017 | 0.0053 | 1.1941 | 0.9356 |
| 406 | | | gamma-glutamyltyrosine | LC/MS pos | 311.12377 | 2734 | | HMDB11741 | 94340 | 0.85 | 0.82 | 0.0166 | 0.0244 | 1.0817 | 0.8885 |
| 407 | | | gamma-glutamylvaline | LC/MS pos | 247.12885 | 43829 | | HMDB11172 | 7015683 | 0.88 | 0.86 | 0.0147 | 0.0228 | 1.0957 | 0.9398 |
| 408 | | | gamma-glutamyl-2-aminobutyrate | LC/MS pos | 233.1132 | 37092 | | | | 1.18 | 1.10 | 0.7803 | 0.3405 | 0.8844 | 0.9689 |
| 410 | | Dipeptide Derivative | N-acetylcarnosine | LC/MS pos | 269.12444 | 43488 | | HMDB12881 | 9903482 | 1.00 | 0.98 | 0.6322 | 0.3013 | 1.0450 | 1.0260 |
| 517 | | Dipeptide | cyclo(val-val) | LC/MS pos | 199.14411 | 49796 | | | 519728 | 1.00 | 0.86 | 0.5622 | 0.2822 | 1.1562 | 0.9924 |
| 545 | | | glycylvaline | LC/MS pos | 175.10772 | 18357 | | HMDB28854 | 97417 | 0.56 | 0.15 | 0.0034 | 0.0079 | 6.9270 | 1.0565 |
| 569 | | | isoleucylglycine | LC/MS pos | 189.12337 | 40008 | | | 342532 | 0.82 | 0.75 | 0.0157 | 0.0234 | 1.1828 | 0.8889 |
| 587 | | | leucylglycine | LC/MS pos | 189.12337 | 40045 | | | 79070 | 0.51 | 0.51 | 0.0001 | 0.0004 | 1.1721 | 0.5961 |
| 656 | | | prolylglycine | LC/MS pos | 173.09207 | 40703 | | | 7408076;6426709 | 0.89 | 0.80 | 0.0837 | 0.0744 | 1.0835 | 0.8663 |
| 696 | | | threonylphenylalanine | LC/MS pos | 267.13394 | 31530 | | | 4099799;4099798 | 0.81 | 0.73 | 0.0449 | 0.0480 | 1.1720 | 0.8526 |
| 735 | | | valylglycine | LC/MS pos | 175.10772 | 40475 | | | 136487 | 0.71 | 0.57 | 0.0054 | 0.0112 | 1.2603 | 0.7244 |
| 738 | | | valylleucine | LC/MS pos | 231.17032 | 39994 | | | 352039 | 0.80 | 0.78 | 0.0039 | 0.0087 | 1.1095 | 0.8613 |
| 751 | | Polypeptide | bradykinin, des-arg(9) | LC/MS pos | 452.73742 | 34420 | C00306 | HMDB04246 | 105044 | 0.84 | 0.68 | 0.0329 | 0.0374 | 1.5525 | 1.0516 |
| 755 | | | HWESASXX* | LC/MS pos | 471.73762 | 32836 | | | | 0.83 | 0.80 | 0.0598 | 0.0599 | 1.3486 | 1.0734 |
| 781 | | Fibrinogen Cleavage Peptide | ADSGEGDFXAEGGGVR* | LC/MS pos | 768.84989 | 33084 | | | 16133137 | 2.00 | 1.18 | 0.3276 | 0.1945 | 0.9326 | 1.1044 |
| 782 | | | DSGEGDFXAEGGGVR* | LC/MS pos | 733.33133 | 31548 | | | | 2.08 | 1.20 | 0.7362 | 0.3303 | 0.9152 | 1.1005 |
| 793 | Carbohydrate | Glycolysis, Gluconeogenesis, and Pyruvate Metabolism | 1,5-anhydroglucitol (1,5-AG) | LC/MS neg | 163.06119 | 20675 | C07326 | HMDB02712 | 64960 | 0.91 | 0.92 | 0.0028 | 0.0073 | 0.9963 | 0.9126 |
| 796 | | | glucose | LC/MS polar | 179.05611 | 20488 | C00031 | HMDB00122 | 79025 | 1.00 | 0.99 | 0.7329 | 0.3294 | 1.0467 | 1.0349 |
| 812 | | | pyruvate | LC/MS polar | 87.00877 | 48990 | C00022 | HMDB00243 | 1060 | 1.60 | 1.42 | 0.0019 | 0.0057 | 0.9167 | 1.3015 |
| 813 | | | lactate | LC/MS polar | 89.02442 | 527 | C00186 | HMDB00190 | 612 | 1.46 | 1.41 | 0.0002 | 0.0009 | 0.8890 | 1.2513 |
| 816 | | | glycerate | LC/MS polar | 105.01933 | 1572 | C00258 | HMDB00139 | 752 | 0.83 | 0.83 | 0.0008 | 0.0032 | 1.0458 | 0.8642 |
| 831 | | Pentose Metabolism | ribose | GC/MS | 204 | 12083 | C00121 | HMDB00283 | 5779 | 1.14 | 1.06 | 0.5913 | 0.2889 | 0.9277 | 0.9788 |
| 832 | | | ribitol | LC/MS polar | 151.06119 | 15772 | C00474 | HMDB00508 | 6912 | 1.03 | 1.02 | 0.9083 | 0.3726 | 1.0396 | 1.0571 |
| 833 | | | ribonate | LC/MS polar | 165.04046 | 27731 | C01685 | HMDB00867 | 5460677 | 0.95 | 0.94 | 0.2785 | 0.1758 | 1.0076 | 0.9487 |
| 836 | | | xylonate | GC/MS | 292 | 35638 | C05411 | HMDB60256 | 6602431 | 1.08 | 1.00 | 0.9999 | 0.3961 | 1.0565 | 1.0544 |
| 837 | | | xylose | GC/MS | 204 | 15835 | C00181 | HMDB00098 | 135191 | 1.31 | 1.19 | 0.5196 | 0.2672 | 1.0809 | 1.2824 |
| 838 | | | xylitol | GC/MS | 307.2 | 4966 | C00379 | HMDB02917 | 6912 | 1.27 | 1.20 | 0.1646 | 0.1217 | 0.9197 | 1.1026 |
| 839 | | | arabinose | GC/MS | 217 | 575 | C00216 | HMDB00646 | 66308 | 1.25 | 1.17 | 0.3259 | 0.1944 | 1.0208 | 1.1953 |
| 842 | | | threitol | GC/MS | 217.1 | 35854 | C16884 | HMDB04136 | 169019 | 1.16 | 1.14 | 0.2020 | 0.1398 | 1.0166 | 1.1617 |
| 844 | | | arabitol | GC/MS | 307.1 | 38075 | C01904 | HMDB00568 | 94154 | 1.00 | 0.94 | 0.5603 | 0.2821 | 1.0551 | 0.9881 |
| 850 | | | fucose | GC/MS | 204 | 15821 | C01018 | HMDB00174 | 19466 | 1.13 | 1.10 | 0.2856 | 0.1779 | 1.0383 | 1.1442 |
| 861 | | Glycogen Metabolism | maltotriose | LC/MS neg | 503.16175 | 44688 | C01835 | HMDB01262 | 439586 | 39.18 | 16.27 | 0.0000 | 0.0000 | 0.0782 | 1.2721 |
| 864 | | | maltose | LC/MS polar | 387.11442 | 15586 | C00208 | HMDB00163 | 10991489 | 8.72 | 6.67 | 0.0000 | 0.0000 | 0.4175 | 2.7867 |
| 886 | | Disaccharides and Oligosaccharides | sucrose | LC/MS neg | 341.10894 | 1519 | C00089 | HMDB00258 | 5988 | 1.60 | 0.82 | 0.6396 | 0.3040 | 1.9134 | 1.5684 |
| 900 | | Fructose, Mannose and Galactose Metabolism | fructose | LC/MS polar | 179.05611 | 577 | C00095 | HMDB00660 | 5984 | 0.92 | 0.67 | 0.0818 | 0.0738 | 1.4563 | 0.9765 |
| 906 | | | mannose | LC/MS polar | 179.05611 | 584 | C00159 | HMDB00169 | 18950 | 1.40 | 1.23 | 0.0197 | 0.0270 | 0.9723 | 1.1979 |
| 910 | | | mannitol | GC/MS | 319.1 | 15335 | C00392 | HMDB00765 | 6251 | 1.34 | 1.03 | 0.8009 | 0.3453 | 1.1207 | 1.1490 |
| 924 | | | galactonate | LC/MS polar | 195.05102 | 27719 | C00880 | HMDB00565 | 128869 | 1.19 | 0.77 | 0.4280 | 0.2323 | 1.6234 | 1.2481 |
| 950 | | Aminosugar Metabolism | glucuronate | LC/MS polar | 193.03537 | 15443 | C00191 | HMDB00127 | 444791 | 1.02 | 1.00 | 0.5349 | 0.2733 | 1.0362 | 1.0334 |
| 961 | | | N-acetylneuraminate | LC/MS polar | 308.0987 | 32377 | C00270 | HMDB00230 | 439197 | 1.02 | 1.00 | 0.7713 | 0.3371 | 1.0008 | 0.9985 |
| 973 | | | erythronate* | LC/MS polar | 135.02989 | 42420 | | HMDB00613 | 2781043 | 1.26 | 1.26 | 0.0003 | 0.0016 | 0.9399 | 1.1804 |
| 980 | Energy | TCA Cycle | citrate | LC/MS neg | 191.01973 | 1564 | C00158 | HMDB00094 | 311 | 6.51 | 5.51 | 0.1201 | 0.0970 | 0.9461 | 5.2152 |
| 982 | | | aconitate [cis or trans] | LC/MS neg | 173.00916 | 46173 | C00417 | HMDB00072 | | 1.29 | 1.22 | 0.0264 | 0.0315 | 0.9360 | 1.1391 |
| 985 | | | alpha-ketoglutarate | LC/MS polar | 145.01425 | 528 | C00026 | HMDB00208 | 51 | 0.98 | 0.96 | 0.4248 | 0.2316 | 1.0262 | 0.9807 |
| 987 | | | succinylcarnitine | LC/MS pos | 262.12852 | 37058 | | | | 1.10 | 1.10 | 0.0557 | 0.0577 | 1.0311 | 1.1354 |
| 988 | | | succinate | LC/MS polar | 117.01933 | 1437 | C00042 | HMDB00254 | 1110 | 0.88 | 0.87 | 0.0704 | 0.0671 | 1.0640 | 0.9279 |
| 989 | | | fumarate | LC/MS polar | 115.00368 | 1643 | C00122 | HMDB00134 | 444972 | 1.10 | 1.04 | 0.5064 | 0.2627 | 1.0186 | 1.0570 |
| 990 | | | malate | LC/MS polar | 133.01425 | 1303 | C00149 | HMDB00156 | 525 | 1.02 | 0.97 | 0.6297 | 0.3006 | 1.1418 | 1.1068 |
| 1000 | | | 2-methylcitrate/homocitrate | LC/MS neg | 205.03538 | 52282 | | | | 0.94 | 0.91 | 0.1793 | 0.1291 | 1.0576 | 0.9598 |
| 1003 | | Oxidative Phosphorylation | phosphate | LC/MS neg | 96.96962 | 42109 | C00009 | HMDB01429 | 1061 | 0.81 | 0.81 | 0.0175 | 0.0248 | 1.0886 | 0.8828 |
| 1004 | | Short Chain Fatty Acid | valerate | LC/MS neg | 101.0608 | 33443 | C00803 | HMDB00892 | 7991 | 0.95 | 0.84 | 0.2023 | 0.1398 | 1.0622 | 0.8885 |
| 1005 | | Medium Chain Fatty Acid | caproate (6:0) | LC/MS neg | 115.07645 | 32489 | C01585 | HMDB00535 | 8892 | 1.08 | 1.05 | 0.7504 | 0.3314 | 1.0679 | 1.1209 |
| 1006 | | | heptanoate (7:0) | LC/MS neg | 129.0921 | 1644 | C17714 | HMDB00666 | 8094 | 0.86 | 0.85 | 0.0157 | 0.0234 | 1.1133 | 0.9497 |
| 1007 | | | caprylate (8:0) | LC/MS neg | 143.10775 | 32492 | C06423 | HMDB00482 | 379 | 1.22 | 1.21 | 0.2506 | 0.1637 | 1.0863 | 1.3186 |
| 1009 | | | caprate (10:0) | LC/MS neg | 171.13905 | 1642 | C01571 | HMDB00511 | 2969 | 1.65 | 1.48 | 0.0274 | 0.0325 | 1.0080 | 1.4871 |
| 1011 | | | 10-undecenoate (11:1n1) | LC/MS neg | 183.13905 | 32497 | | | | 1.15 | 1.04 | 0.4756 | 0.2512 | 1.1526 | 1.1938 |
| 1012 | | | laurate (12:0) | LC/MS neg | 199.17035 | 1645 | C02679 | HMDB00638 | 3893 | 1.49 | 1.39 | 0.0146 | 0.0228 | 0.9576 | 1.3346 |
| 1013 | | | 5-dodecenoate (12:1n7) | LC/MS neg | 197.1547 | 33968 | | HMDB00529 | 5312378 | 1.32 | 1.34 | 0.1766 | 0.1280 | 1.1826 | 1.5857 |
| 1015 | | Long Chain Fatty Acid | myristate (14:0) | LC/MS neg | 227.20165 | 1365 | C06424 | HMDB00806 | 11005 | 1.64 | 1.51 | 0.0247 | 0.0300 | 1.0213 | 1.5461 |
| 1016 | | | myristoleate (14:1n5) | LC/MS neg | 225.186 | 32418 | C08322 | HMDB02000 | 5281119 | 1.80 | 1.64 | 0.0233 | 0.0293 | 1.3317 | 2.1785 |
| 1017 | | | pentadecanoate (15:0) | LC/MS neg | 241.2173 | 1361 | C16537 | HMDB00826 | 13849 | 1.40 | 1.36 | 0.0022 | 0.0062 | 1.0312 | 1.3977 |
| 1018 | | | palmitate (16:0) | LC/MS neg | 255.23295 | 1336 | C00249 | HMDB00220 | 985 | 1.24 | 1.24 | 0.0193 | 0.0267 | 1.0105 | 1.2489 |
| 1019 | | | palmitoleate (16:1n7) | LC/MS neg | 253.2173 | 33447 | C08362 | HMDB03229 | 445638 | 1.85 | 1.73 | 0.0754 | 0.0696 | 1.3848 | 2.3988 |
| 1021 | | | margarate (17:0) | LC/MS neg | 269.2486 | 1121 | | HMDB02259 | 10465 | 1.55 | 1.50 | 0.0064 | 0.0127 | 1.0301 | 1.5499 |
| 1022 | | | 10-heptadecenoate (17:1n7) | LC/MS neg | 267.23295 | 33971 | | HMDB60038 | 5312435 | 1.77 | 1.65 | 0.0198 | 0.0270 | 1.0032 | 1.6548 |
| 1023 | | | stearate (18:0) | LC/MS neg | 283.26425 | 1358 | C01530 | HMDB00827 | 5281 | 1.23 | 1.23 | 0.0202 | 0.0273 | 0.9912 | 1.2197 |
| 1025 | | | oleate (18:1n9) | GC/MS | 339.2 | 1359 | C00712 | HMDB00207 | 445639 | 1.70 | 1.61 | 0.0086 | 0.0156 | 1.0068 | 1.6250 |
| 1027 | | | cis-vaccenate (18:1n7) | GC/MS | 339.3 | 33970 | C08367 | HMDB03231 | 5282761 | 1.58 | 1.38 | 0.0110 | 0.0182 | 0.9850 | 1.3596 |
| 1031 | | | nonadecanoate (19:0) | LC/MS neg | 297.2799 | 1356 | C16535 | HMDB00772 | 12591 | 1.34 | 1.35 | 0.0008 | 0.0032 | 0.9524 | 1.2840 |
| 1032 | | | 10-nonadecenoate (19:1n9) | LC/MS neg | 295.26425 | 33972 | | HMDB13622 | 5312513 | 1.88 | 1.74 | 0.0044 | 0.0094 | 0.9312 | 1.6195 |
| 1034 | | | arachidate (20:0) | LC/MS neg | 311.29555 | 1118 | C06425 | HMDB02212 | 10467 | 1.31 | 1.18 | 0.1587 | 0.1189 | 1.0427 | 1.2306 |
| 1037 | | | eicosenoate (20:1) | LC/MS neg | 309.2799 | 33587 | | HMDB02231 | 5282768 | 2.04 | 1.97 | 0.0001 | 0.0005 | 0.8493 | 1.6754 |
| 1041 | | | erucate (22:1n9) | LC/MS neg | 337.3112 | 1552 | C08316 | HMDB02068 | 5281116 | 2.39 | 1.38 | 0.0463 | 0.0493 | 1.0137 | 1.4032 |
| 1050 | | Polyunsaturated Fatty Acid (n3 and n6) | stearidonate (18:4n3) | LC/MS neg | 275.20165 | 33969 | C16300 | HMDB06547 | 5312508 | 1.33 | 1.23 | 0.1590 | 0.1189 | 1.2865 | 1.5873 |
| 1051 | | | eicosapentaenoate (EPA; 20:5n3) | LC/MS neg | 301.2173 | 18467 | C06428 | HMDB01999 | 446284 | 0.83 | 0.79 | 0.0596 | 0.0599 | 1.6164 | 1.2783 |
| 1052 | | | docosapentaenoate (n3 DPA; 22:5n3) | LC/MS neg | 329.2486 | 32504 | C16513 | HMDB01976 | 6441454 | 1.45 | 1.37 | 0.0277 | 0.0326 | 0.9218 | 1.2634 |
| 1053 | | | docosahexaenoate (DHA; 22:6n3) | LC/MS neg | 327.23295 | 44675 | C06429 | HMDB02183 | 445580 | 0.96 | 0.90 | 0.3363 | 0.1955 | 1.3251 | 1.1889 |
| 1057 | | | linoleate (18:2n6) | LC/MS neg | 279.23295 | 1105 | C01595 | HMDB00673 | 5280450 | 1.42 | 1.38 | 0.0222 | 0.0288 | 1.0030 | 1.3855 |
| 1059 | | | linolenate [alpha or gamma; (18:3n3 or 6)] | LC/MS neg | 277.2173 | 34035 | C06426 | HMDB03073 | 5280934 | 1.80 | 1.70 | 0.0101 | 0.0173 | 0.9509 | 1.6206 |
| 1061 | | | dihomo-linolenate (20:3n3 or n6) | LC/MS neg | 305.2486 | 35718 | C03242 | HMDB02925 | 5280581 | 0.90 | 0.86 | 0.1201 | 0.0970 | 1.1769 | 1.0136 |
| 1062 | | | arachidonate (20:4n6) | LC/MS neg | 303.23295 | 1110 | C00219 | HMDB01043 | 444899 | 0.76 | 0.73 | 0.0093 | 0.0168 | 1.1828 | 0.8666 |
| 1063 | | | adrenate (22:4n6) | LC/MS neg | 331.26425 | 32980 | C16527 | HMDB02226 | 5497181 | 2.36 | 2.25 | 0.0000 | 0.0001 | 0.7346 | 1.6524 |
| 1064 | | | docosapentaenoate (n6 DPA; 22:5n6) | LC/MS neg | 329.2486 | 37478 | C16513 | HMDB01976 | 6441454 | 0.86 | 0.81 | 0.0397 | 0.0432 | 1.2608 | 1.0182 |
| 1065 | | | docosadienoate (22:2n6) | LC/MS neg | 335.29555 | 32415 | C16533 | HMDB61714 | 5282807 | 2.96 | 2.79 | 0.0000 | 0.0000 | 0.6261 | 1.7463 |
| 1066 | | | dihomo-linoleate (20:2n6) | LC/MS neg | 307.26425 | 17805 | C16525 | HMDB05060 | 6439848 | 1.90 | 1.77 | 0.0002 | 0.0010 | 0.8468 | 1.4958 |
| 1116 | | Fatty Acid, Branched | 13-methylmyristic acid | LC/MS neg | 241.2173 | 38293 | | | 151014 | 1.40 | 1.28 | 0.0813 | 0.0737 | 0.9630 | 1.2317 |
| 1119 | | | 15-methylpalmitate (isobar with 2-methylpalmitate) | LC/MS neg | 269.24868 | 38768 | | | 17903417 | 1.34 | 1.32 | 0.3212 | 0.1930 | 1.0557 | 1.3975 |
| 1122 | | | 17-methylstearate | LC/MS neg | 297.2799 | 38296 | | | 3083779 | 1.71 | 1.63 | 0.0019 | 0.0057 | 0.7957 | 1.2957 |
| 1133 | | Fatty Acid, Dicarboxylate | 2-hydroxyglutarate | LC/MS polar | 147.02989 | 37253 | C02630 | HMDB00606 | 43 | 1.71 | 1.61 | 0.0214 | 0.0284 | 0.9920 | 1.5942 |
| 1136 | | | 2-hydroxyadipate | LC/MS polar | 161.04554 | 31934 | C02360 | HMDB00321 | 193530 | 0.88 | 0.68 | 0.0558 | 0.0577 | 1.1531 | 0.7814 |
| 1137 | | | 3-methyladipate | LC/MS polar | 159.06628 | 36749 | | HMDB00555 | 12292 | 1.45 | 1.35 | 0.1395 | 0.1088 | 0.9878 | 1.3318 |
| 1139 | | | maleate | LC/MS polar | 115.00368 | 20676 | C01384 | HMDB00176 | 444266 | 0.77 | 0.73 | 0.0070 | 0.0134 | 1.3816 | 1.0110 |
| 1142 | | | suberate (octanedioate) | LC/MS polar | 173.08193 | 15730 | C08278 | HMDB00893 | 10457 | 1.23 | 0.79 | 0.3338 | 0.1953 | 2.0104 | 1.5913 |
| 1145 | | | azelate (nonanedioate) | LC/MS neg | 187.09758 | 18362 | C08261 | HMDB00784 | 2266 | 1.00 | 0.75 | 0.1780 | 0.1286 | 1.8739 | 1.4075 |
| 1146 | | | sebacate (decanedioate) | LC/MS neg | 201.11323 | 32398 | C08277 | HMDB00792 | 5192 | 1.37 | 1.31 | 0.0662 | 0.0654 | 0.9876 | 1.2948 |
| 1149 | | | dodecanedioate | LC/MS neg | 229.14453 | 32388 | C02678 | HMDB00623 | 12736 | 1.28 | 1.23 | 0.0541 | 0.0564 | 1.0110 | 1.2428 |
| 1150 | | | tetradecanedioate | LC/MS neg | 257.17583 | 35669 | | HMDB00872 | 13185 | 1.12 | 1.08 | 0.3204 | 0.1930 | 1.2258 | 1.3216 |
| 1151 | | | hexadecanedioate | LC/MS neg | 285.20713 | 35678 | C19615 | HMDB00672 | 10459 | 1.71 | 1.68 | 0.0009 | 0.0034 | 0.9444 | 1.5827 |
| 1152 | | | octadecanedioate | LC/MS neg | 313.23843 | 36754 | | HMDB00782 | 70095 | 2.08 | 1.94 | 0.0003 | 0.0013 | 0.9011 | 1.7485 |
| 1153 | | | eicosanodioate | LC/MS neg | 341.26973 | 39831 | | | 75502 | 0.95 | 0.78 | 0.2812 | 0.1769 | 1.3290 | 1.0409 |
| 1155 | | | 3-carboxy-4-methyl-5-propyl-2-furanpropanoate (CMPF) | LC/MS neg | 239.09249 | 31787 | | HMDB61112 | 123979 | 0.89 | 0.96 | 0.1013 | 0.0862 | 2.0180 | 1.9357 |
| 1180 | | Fatty Acid, Amide | oleamide | LC/MS neg | 280.26458 | 32458 | C19670 | HMDB02117 | 5283387 | 0.69 | 0.37 | 0.0105 | 0.0176 | 2.6343 | 0.9708 |
| 1186 | | Fatty Acid, Amino | 2-aminoheptanoate | LC/MS pos | 146.11756 | 43761 | | | 227939 | 1.00 | 0.94 | 0.4756 | 0.2512 | 1.0619 | 0.9952 |
| 1191 | | | 2-aminooctanoate | LC/MS pos | 160.13321 | 43343 | | HMDB00991 | 69522 | 1.16 | 0.94 | 0.9291 | 0.3793 | 1.1555 | 1.0818 |
| 1199 | | Fatty Acid Synthesis | malonylcarnitine | LC/MS pos | 248.11287 | 37059 | | HMDB02095 | 22833583 | 1.05 | 0.99 | 0.8270 | 0.3524 | 0.9712 | 0.9581 |
| 1200 | | | malonate | LC/MS polar | 103.00368 | 15872 | C00383 | HMDB00691 | 867 | 2.34 | 2.12 | 0.1487 | 0.1132 | 1.0365 | 2.1925 |
| 1201 | | | 2-methylmalonyl carnitine | LC/MS pos | 262.12852 | 35482 | | HMDB13133 | 53481628 | 1.05 | 1.02 | 0.8324 | 0.3532 | 0.9749 | 0.9922 |
| 1213 | | Fatty Acid Metabolism (also BCAA Metabolism) | butyrylcarnitine | LC/MS pos | 232.15434 | 32412 | C02862 | HMDB02013 | 439829 | 1.29 | 1.25 | 0.0010 | 0.0035 | 1.0910 | 1.3637 |
| 1214 | | | butyrylglycine | LC/MS neg | 144.06661 | 31850 | | HMDB00808 | 88412 | 0.96 | 0.90 | 0.3037 | 0.1860 | 1.5664 | 1.4076 |
| 1216 | | | propionylcarnitine | LC/MS pos | 218.13869 | 32452 | C03017 | HMDB00824 | 107738 | 0.84 | 0.84 | 0.0021 | 0.0061 | 1.0682 | 0.8925 |
| 1217 | | | propionylglycine | LC/MS pos | 132.06552 | 31932 | | HMDB00783 | 98681 | 0.78 | 0.77 | 0.0349 | 0.0391 | 1.0967 | 0.8400 |
| 1219 | | | methylmalonate (MMA) | LC/MS polar | 117.01933 | 1496 | C02170 | HMDB00202 | 487 | 0.91 | 0.92 | 0.1045 | 0.0880 | 1.0177 | 0.9355 |
| 1222 | | Fatty Acid Metabolism(Acyl Glycine) | hexanoylglycine | LC/MS neg | 172.09791 | 35436 | | HMDB00701 | 99463 | 1.38 | 1.45 | 0.0237 | 0.0294 | 0.9499 | 1.3790 |
| 1225 | | | N-palmitoyl glycine | LC/MS neg | 312.25441 | 42092 | | | 151008 | 1.23 | 1.18 | 0.1112 | 0.0921 | 0.9318 | 1.1001 |
| 1230 | | Fatty Acid Metabolism(Acyl Carnitine) | acetylcarnitine | LC/MS pos | 204.12304 | 32198 | C02571 | HMDB00201 | 1 | 1.09 | 1.08 | 0.2524 | 0.1645 | 0.9316 | 1.0068 |
| 1231 | | | hydroxybutyrylcarnitine* | LC/MS pos | 248.14925 | 43264 | | HMDB13127 | 53481617 | 1.49 | 1.44 | 0.0006 | 0.0025 | 0.8635 | 1.2458 |
| 1233 | | | hexanoylcarnitine | LC/MS pos | 260.18564 | 32328 | | HMDB00705 | 6426853 | 1.39 | 1.37 | 0.0010 | 0.0035 | 1.0121 | 1.3885 |
| 1234 | | | octanoylcarnitine | LC/MS pos | 288.21694 | 33936 | C02838 | HMDB00791 | 123701 | 1.32 | 1.12 | 0.0998 | 0.0852 | 1.0252 | 1.1431 |
| 1236 | | | decanoylcarnitine | LC/MS pos | 316.24824 | 33941 | | HMDB00651 | 10245190 | 1.42 | 1.13 | 0.0880 | 0.0777 | 0.9643 | 1.0899 |
| 1238 | | | cis-4-decenoyl carnitine | LC/MS pos | 314.23259 | 38178 | | | | 1.05 | 1.05 | 0.9411 | 0.3821 | 0.9371 | 0.9856 |
| 1239 | | | laurylcarnitine | LC/MS pos | 344.27954 | 34534 | | HMDB02250 | 10427569 | 2.86 | 1.82 | 0.0696 | 0.0667 | 0.7475 | 1.3612 |
| 1242 | | | palmitoylcarnitine | LC/MS pos | 400.34214 | 44681 | C02990 | HMDB00222 | 461 | 2.77 | 2.31 | 0.2703 | 0.1735 | 0.9507 | 2.1947 |
| 1244 | | | linoleoylcarnitine* | LC/MS pos | 424.34214 | 46223 | | HMDB06469 | 6450015 | 4.73 | 3.60 | 0.2368 | 0.1575 | 0.3817 | 1.3723 |
| 1245 | | | oleoylcarnitine | LC/MS pos | 426.35779 | 35160 | | HMDB05065 | 6441392;53477789 | 3.77 | 2.98 | 0.0586 | 0.0596 | 0.7766 | 2.3145 |
| 1247 | | | myristoleoylcarnitine* | LC/MS pos | 370.29471 | 48182 | | | | 3.62 | 2.54 | 0.0247 | 0.0300 | 0.6640 | 1.6882 |
| 1248 | | Carnitine Metabolism | deoxycarnitine | LC/MS pos | 146.11756 | 36747 | C01181 | HMDB01161 | 134 | 1.02 | 1.02 | 0.8811 | 0.3674 | 1.0017 | 1.0174 |
| 1249 | | | carnitine | LC/MS pos | 162.11247 | 15500 | C00318 | HMDB00062 | 10917 | 1.07 | 1.07 | 0.0034 | 0.0079 | 0.9669 | 1.0327 |
| 1252 | | Ketone Bodies | acetoacetate | LC/MS pos | 103.03898 | 33963 | C00164 | HMDB00060 | 96 | 1.20 | 0.97 | 0.7890 | 0.3418 | 1.1123 | 1.0767 |
| 1254 | | | 3-hydroxybutyrate (BHBA) | LC/MS polar | 103.04007 | 542 | C01089 | HMDB00357 | 441 | 1.03 | 1.00 | 0.7415 | 0.3304 | 1.1796 | 1.1834 |
| 1257 | | Fatty Acid, Monohydroxy | alpha-hydroxycaproate | LC/MS neg | 131.07136 | 37073 | | HMDB01624 | 99824 | 1.19 | 1.23 | 0.5691 | 0.2840 | 1.0678 | 1.3089 |
| 1258 | | | 2-hydroxyoctanoate | LC/MS neg | 159.10266 | 22036 | | HMDB02264 | 94180 | 0.94 | 0.85 | 0.2385 | 0.1579 | 1.1505 | 0.9768 |
| 1259 | | | 2-hydroxydecanoate | LC/MS neg | 187.13396 | 42489 | | | 21488 | 1.18 | 0.89 | 0.5437 | 0.2762 | 1.2932 | 1.1506 |
| 1261 | | | 2-hydroxypalmitate | LC/MS neg | 271.22786 | 35675 | | HMDB31057 | 92836 | 0.90 | 0.85 | 0.0878 | 0.0777 | 1.0830 | 0.9220 |
| 1262 | | | 2-hydroxystearate | LC/MS neg | 299.25917 | 17945 | C03045 | | 69417 | 0.88 | 0.83 | 0.0525 | 0.0554 | 1.1743 | 0.9778 |
| 1265 | | | 3-hydroxyoctanoate | LC/MS neg | 159.10266 | 22001 | | HMDB01954 | 26613 | 1.31 | 1.19 | 0.1614 | 0.1203 | 1.0634 | 1.2665 |
| 1266 | | | 3-hydroxydecanoate | LC/MS neg | 187.13396 | 22053 | | HMDB02203 | 26612 | 1.37 | 1.25 | 0.0175 | 0.0248 | 1.0661 | 1.3361 |
| 1267 | | | 3-hydroxysebacate | LC/MS neg | 217.10814 | 31943 | | HMDB00350 | 3017884 | 2.15 | 1.72 | 0.0041 | 0.0089 | 0.9934 | 1.7070 |
| 1268 | | | 3-hydroxylaurate | LC/MS neg | 215.16526 | 32457 | | HMDB00387 | 94216 | 1.57 | 1.36 | 0.0234 | 0.0293 | 0.9687 | 1.3179 |
| 1273 | | | 5-hydroxyhexanoate | LC/MS neg | 131.07136 | 31938 | | HMDB00525 | 170748 | 1.16 | 1.07 | 0.4059 | 0.2255 | 1.0430 | 1.1150 |
| 1280 | | | 16-hydroxypalmitate | LC/MS neg | 271.22786 | 39609 | C18218 | HMDB06294 | 10466 | 1.44 | 1.39 | 0.0246 | 0.0300 | 0.9760 | 1.3539 |
| 1285 | | | 13-HODE + 9-HODE | LC/MS neg | 295.22825 | 37752 | | | 43013 | 1.12 | 0.96 | 0.9724 | 0.3895 | 1.3972 | 1.3377 |
| 1295 | | Fatty Acid, Dihydroxy | 12,13-DiHOME | LC/MS neg | 313.23843 | 38395 | C14829 | HMDB04705 | 10236635 | 1.60 | 1.22 | 0.3483 | 0.2002 | 0.9774 | 1.1947 |
| 1296 | | | 9,10-DiHOME | LC/MS neg | 313.23843 | 38399 | C14828 | HMDB04704 | 9966640 | 1.12 | 1.10 | 0.2207 | 0.1488 | 1.1846 | 1.3070 |
| 1361 | | Eicosanoid | 5-HETE | LC/MS neg | 319.22786 | 37372 | C04805 | HMDB11134 | 5280733 | 1.99 | 1.77 | 0.0187 | 0.0259 | 0.6777 | 1.1989 |
| 1366 | | | 12-HETE | LC/MS neg | 319.22786 | 37536 | | HMDB06111 | 5312983 | 0.60 | 0.31 | 0.0011 | 0.0036 | 2.9009 | 0.9121 |
| 1376 | | Endocannabinoid | oleic ethanolamide | LC/MS neg | 324.2908 | 38102 | | HMDB02088 | 5283454 | 1.14 | 1.08 | 0.7185 | 0.3253 | 1.0167 | 1.0956 |
| 1387 | | | N-stearoyltaurine | LC/MS neg | 390.26835 | 39730 | | | 168274 | 1.26 | 1.01 | 0.9724 | 0.3895 | 0.9774 | 0.9827 |
| 1391 | | Inositol Metabolism | myo-inositol | LC/MS polar | 225.06159 | 1124 | C00137 | HMDB00211 | 892 | 0.87 | 0.88 | 0.0293 | 0.0342 | 1.0789 | 0.9455 |
| 1392 | | | chiro-inositol | GC/MS | 318.1 | 37112 | C19891 | HMDB34220 | | 3.00 | 1.12 | 0.8301 | 0.3531 | 1.5734 | 1.7646 |
| 1394 | | | scyllo-inositol | GC/MS | 318.2 | 32379 | C06153 | HMDB06088 | 892 | 0.79 | 0.71 | 0.0096 | 0.0169 | 1.5570 | 1.0997 |
| 1399 | | | inositol 1-phosphate (I1P) | GC/MS | 318.1 | 1481 | C04006 | HMDB00213 | 440194 | 0.78 | 0.75 | 0.0029 | 0.0073 | 1.1882 | 0.8856 |
| 1418 | | Phospholipid Metabolism | choline | LC/MS pos | 104.10699 | 15506 | C00114 | HMDB00097 | 305 | 0.89 | 0.89 | 0.0065 | 0.0127 | 1.0740 | 0.9552 |
| 1419 | | | choline phosphate | LC/MS pos | 184.07332 | 34396 | C00588 | HMDB01565 | 1014 | 0.38 | 0.24 | 0.0000 | 0.0002 | 1.5486 | 0.3782 |
| 1421 | | | glycerophosphorylcholine (GPC) | LC/MS pos | 258.1101 | 15990 | C00670 | HMDB00086 | 71920 | 0.77 | 0.76 | 0.0017 | 0.0051 | 1.1454 | 0.8651 |
| 1424 | | | ethanolamine | GC/MS | 174.1 | 1497 | C00189 | HMDB00149 | 700 | 1.02 | 1.00 | 0.8639 | 0.3620 | 1.0190 | 1.0169 |
| 1427 | | | glycerophosphoethanolamine | LC/MS polar | 214.04859 | 37455 | C01233 | HMDB00114 | 123874 | 0.98 | 0.97 | 0.4561 | 0.2432 | 0.9595 | 0.9343 |
| 1428 | | | trimethylamine N-oxide | LC/MS pos | 151.14411 | 40406 | C01104 | HMDB00925 | 1145 | 2.34 | 2.51 | 0.7706 | 0.3371 | 1.7278 | 4.3427 |
| 1429 | | | glycerophosphoinositol* | LC/MS polar | 333.05922 | 47155 | | | | 1.71 | 1.29 | 0.6174 | 0.2959 | 0.9448 | 1.2208 |
| 1430 | | | 1-palmitoyl-2-oleoyl-GPC (16:0/18:1)* | LC/MS polar | 804.57604 | 52461 | | | 6436017 | 0.97 | 0.96 | 0.3560 | 0.2028 | 1.0222 | 0.9765 |
| 1431 | | | 1-stearoyl-2-arachidonoyl-GPC (18:0/20:4) | LC/MS polar | 854.59091 | 42450 | | | 16219824 | 0.90 | 0.86 | 0.0830 | 0.0743 | 1.2047 | 1.0307 |
| 1432 | | | 1-palmitoyl-2-linoleoyl-GPC (16:0/18:2)* | LC/MS polar | 802.56018 | 52460 | | | 5287971 | 0.93 | 0.93 | 0.0722 | 0.0680 | 1.0673 | 0.9914 |
| 1433 | | | 1,2-dipalmitoyl-GPC (16:0/16:0) | LC/MS polar | 778.5604 | 19130 | | HMDB00564 | 452110 | 1.10 | 1.09 | 0.3692 | 0.2090 | 0.9167 | 0.9994 |
| 1434 | | | 1-stearoyl-2-oleoyl-GPC (18:0/18:1) | LC/MS polar | 832.6073 | 52438 | | | | 0.91 | 0.88 | 0.1237 | 0.0992 | 1.0690 | 0.9435 |
| 1436 | | | 1-palmitoyl-2-arachidonoyl-GPC (16:0/20:4)* | LC/MS polar | 826.56034 | 52459 | | | 10747814 | 0.88 | 0.87 | 0.0036 | 0.0081 | 1.0829 | 0.9405 |
| 1437 | | | 1-stearoyl-2-linoleoyl-GPC (18:0/18:2)* | LC/MS polar | 830.59165 | 52452 | | | | 0.89 | 0.87 | 0.0594 | 0.0599 | 1.0246 | 0.8948 |
| 1440 | | | 1-palmitoyl-2-palmitoleoyl-GPC (16:0/16:1)* | LC/MS polar | 776.5447 | 52470 | | | | 0.96 | 0.85 | 0.3125 | 0.1900 | 1.2584 | 1.0713 |
| 1443 | | | 1-(1-enyl-palmitoyl)-2-oleoyl-GPC (P-16:0/18:1)* | LC/MS polar | 788.58109 | 52478 | | | | 1.26 | 0.93 | 0.7901 | 0.3418 | 0.8943 | 0.8284 |
| 1444 | | | 1-stearoyl-2-arachidonoyl-GPI (18:0/20:4)* | LC/MS polar | 885.54948 | 52449 | | | | 0.86 | 0.82 | 0.0051 | 0.0108 | 1.2290 | 1.0081 |
| 1445 | | | 1-palmitoyl-2-linoleoyl-GPI (16:0/18:2)* | LC/MS polar | 833.51845 | 52450 | | | | 0.78 | 0.76 | 0.0029 | 0.0073 | 1.1804 | 0.8935 |
| 1446 | | | 1-oleoyl-2-linoleoyl-GPI (18:1/18:2)* | LC/MS polar | 859.53393 | 52451 | | | | 0.74 | 0.70 | 0.0006 | 0.0025 | 1.2817 | 0.8955 |
| 1447 | | | 1-palmitoyl-2-arachidonoyl-GPI (16:0/20:4)* | LC/MS polar | 857.51855 | 52467 | | | | 0.70 | 0.66 | 0.0010 | 0.0035 | 1.2151 | 0.7965 |
| 1448 | | | 1-stearoyl-2-linoleoyl-GPI (18:0/18:2)* | LC/MS polar | 861.54985 | 52468 | | | | 0.79 | 0.78 | 0.0031 | 0.0075 | 1.1257 | 0.8800 |
| 1451 | | | 1-palmitoyl-2-oleoyl-GPG (16:0/18:1)* | LC/MS polar | 747.51853 | 52448 | | | 5283509 | 1.10 | 1.04 | 0.7099 | 0.3231 | 1.1149 | 1.1559 |

**Comparison mean values significantly different:**
- (red) $p \le 0.05$, fold of change ≥ 1.00
- (dark green) $p \le 0.05$, fold of change < 1.00

**Comparison mean value difference approaching significance:**
- (pink) $0.05 < p < 0.10$, fold of change ≥ 1.00
- (light green) $0.05 < p < 0.10$, fold of change < 1.00

| Pathway Sort Order | Super Pathway | Sub Pathway | Biochemical Name | Platform | Mass | Comp ID | KEGG | HMDB | PubChem | Fold of Change, Paired t-Test: Post G-CSF / Pre G-CSF: Mean Pairs Ratio | Group Mean Ratio | Paired t-Test: Post G-CSF / Pre G-CSF: p-value | q-value | Mean Values: Pre G-CSF | Post G-CSF |
|---|---|---|---|---|---|---|---|---|---|---|---|---|---|---|---|
| 1453 | Lipid | | 1-(1-enyl-stearoyl)-2-arachidonoyl-GPE (P-18:0/20:4)* | LC/MS polar | 750.54431 | 52475 | | | | 0.96 | 0.93 | 0.3066 | 0.1873 | 1.1298 | 1.0458 |
| 1454 | | | 1-(1-enyl-stearoyl)-2-docosahexaenoyl-GPE (P-18:0/22:6)* | LC/MS polar | 774.54431 | 52476 | | | | 1.08 | 1.00 | 0.9702 | 0.3895 | 1.1649 | 1.1669 |
| 1456 | | | 1-palmitoyl-2-oleoyl-GPE (16:0/18:1) | LC/MS polar | 716.52357 | 19263 | | HMDB05320 | 5283496 | 0.99 | 0.88 | 0.3681 | 0.2090 | 1.0473 | 0.9226 |
| 1458 | | | 1-stearoyl-2-arachidonoyl-GPE (18:0/20:4)* | LC/MS polar | 766.53922 | 52447 | | | | 1.00 | 0.96 | 0.6628 | 0.3112 | 1.0365 | 0.9950 |
| 1459 | | | 1-stearoyl-2-docosahexaenoyl-GPE (18:0/22:6)* | LC/MS polar | 790.53922 | 52466 | | | | 1.11 | 0.95 | 0.6260 | 0.2994 | 1.2806 | 1.2189 |
| 1460 | | | 1-stearoyl-2-oleoyl-GPE (18:0/18:1) | LC/MS polar | 744.55488 | 42448 | | | | 1.83 | 1.23 | 0.1075 | 0.0902 | 0.7743 | 0.9548 |
| 1461 | | | 1-(1-enyl-palmitoyl)-GPC (P-16:0)* | LC/MS polar | 524.33577 | 52474 | | | 10917802 | 0.79 | 0.77 | 0.0003 | 0.0016 | 1.1293 | 0.8739 |
| 1462 | | | 1-palmitoyl-2-arachidonoyl-GPE (16:0/20:4)* | LC/MS polar | 738.50792 | 52464 | | | | 1.03 | 0.95 | 0.7670 | 0.3364 | 1.0369 | 0.9799 |
| 1463 | | | 1-palmitoyl-2-docosahexaenoyl-GPE (16:0/22:6)* | LC/MS polar | 762.50792 | 52465 | | | | 1.12 | 0.96 | 0.7135 | 0.3239 | 1.4741 | 1.4083 |
| 1464 | | | 1-palmitoyl-2-linoleoyl-GPE (16:0/18:2) | LC/MS polar | 714.50792 | 42449 | | HMDB05322 | 9546747 | 1.12 | 0.99 | 0.7200 | 0.3253 | 1.0925 | 1.0797 |
| 1465 | | | 1-stearoyl-2-linoleoyl-GPE (18:0/18:2)* | LC/MS polar | 742.5392 | 52446 | | | | 0.93 | 0.92 | 0.1404 | 0.1089 | 1.1628 | 1.0644 |
| 1471 | | Lysolipid | 1-palmitoyl-GPC (16:0) | LC/MS pos | 496.33977 | 33955 | | HMDB10382 | 86554 | 1.06 | 0.87 | 0.1800 | 0.1292 | 1.3091 | 1.1433 |
| 1472 | | | 2-palmitoyl-GPC (16:0)* | LC/MS pos | 496.33977 | 35253 | | HMDB61702 | 15061532 | 1.08 | 0.87 | 0.1967 | 0.1389 | 1.3901 | 1.2148 |
| 1473 | | | 1-palmitoleoyl-GPC (16:1)* | LC/MS pos | 494.32412 | 33230 | | HMDB10383 | 24779461 | 0.90 | 0.79 | 0.0667 | 0.0654 | 1.4601 | 1.1500 |
| 1474 | | | 2-palmitoleoyl-GPC (16:1)* | LC/MS pos | 494.32412 | 35819 | | | | 1.12 | 0.91 | 0.4853 | 0.2547 | 1.0618 | 0.9612 |
| 1477 | | | 1-stearoyl-GPC (18:0) | LC/MS pos | 524.37107 | 33961 | | HMDB10384 | 497299 | 1.15 | 0.85 | 0.2012 | 0.1398 | 1.6766 | 1.4276 |
| 1478 | | | 2-stearoyl-GPC (18:0)* | LC/MS pos | 524.37107 | 35255 | | | 10208382 | 1.26 | 0.85 | 0.1133 | 0.0936 | 1.6241 | 1.3864 |
| 1479 | | | 1-oleoyl-GPC (18:1) | LC/MS pos | 522.35542 | 48258 | | HMDB02815 | 16081932 | 1.13 | 1.01 | 0.3288 | 0.1948 | 1.3328 | 1.3464 |
| 1482 | | | 1-linoleoyl-GPC (18:2) | LC/MS pos | 520.33977 | 34419 | C04100 | HMDB10386 | 11988421 | 1.17 | 0.92 | 0.4091 | 0.2265 | 1.4350 | 1.3218 |
| 1484 | | | 1-linolenoyl-GPC (18:3)* | LC/MS pos | 518.32412 | 45951 | | | | 1.08 | 0.85 | 0.2002 | 0.1398 | 1.4876 | 1.2706 |
| 1494 | | | 1-arachidonoyl-GPC (20:4)* | LC/MS pos | 544.33977 | 33228 | C05208 | HMDB10395 | | 1.23 | 1.17 | 0.8088 | 0.3470 | 1.1068 | 1.3001 |
| 1505 | | | 1-(1-enyl-palmitoyl)-GPE (P-16:0)* | LC/MS neg | 436.28334 | 39270 | | | | 1.54 | 1.06 | 0.7613 | 0.3345 | 1.3034 | 1.3826 |
| 1506 | | | 1-(1-enyl-stearoyl)-GPE (P-18:0)* | LC/MS neg | 464.31464 | 39271 | | | | 0.90 | 0.84 | 0.0884 | 0.0778 | 1.1594 | 0.9687 |
| 1507 | | | 1-(1-enyl-oleoyl)-GPE (P-18:1)* | LC/MS neg | 462.29899 | 44621 | | | | 2.23 | 1.27 | 0.3766 | 0.2122 | 1.3984 | 1.7765 |
| 1510 | | | 1-palmitoyl-GPE (16:0) | LC/MS neg | 452.27826 | 35631 | | HMDB11503 | 9547069 | 0.77 | 0.73 | 0.0030 | 0.0073 | 1.2350 | 0.9027 |
| 1513 | | | 1-stearoyl-GPE (18:0) | LC/MS neg | 480.30956 | 42398 | | HMDB11130 | 9547068 | 2.73 | 0.89 | 0.7224 | 0.3259 | 2.2349 | 1.9810 |
| 1514 | | | 2-stearoyl-GPE (18:0)* | LC/MS neg | 480.30956 | 41220 | | | | 1.20 | 1.01 | 0.6858 | 0.3167 | 1.2215 | 1.2307 |
| 1515 | | | 1-oleoyl-GPE (18:1) | LC/MS neg | 478.29391 | 35628 | | HMDB11506 | 9547071 | 1.12 | 0.86 | 0.5551 | 0.2809 | 1.1738 | 1.0091 |
| 1519 | | | 1-linoleoyl-GPE (18:2)* | LC/MS neg | 476.27826 | 32635 | | HMDB11507 | 52925130 | 0.84 | 0.76 | 0.0228 | 0.0289 | 1.1630 | 0.8872 |
| 1523 | | | 1-arachidonoyl-GPE (20:4)* | LC/MS neg | 500.27826 | 35186 | | HMDB11517 | 42607465 | 0.96 | 0.90 | 0.2710 | 0.1735 | 1.1057 | 0.9908 |
| 1533 | | | 1-palmitoyl-GPI (16:0)* | LC/MS neg | 571.28888 | 35305 | | HMDB61695 | | 0.68 | 0.61 | 0.0058 | 0.0119 | 1.3543 | 0.8292 |
| 1536 | | | 1-stearoyl-GPI (18:0) | LC/MS neg | 599.32018 | 19324 | | HMDB61696 | | 0.85 | 0.73 | 0.0535 | 0.0560 | 1.3270 | 0.9626 |
| 1538 | | | 1-oleoyl-GPI (18:1)* | LC/MS neg | 597.30453 | 36602 | | | | 0.59 | 0.55 | 0.0005 | 0.0022 | 1.3719 | 0.7493 |
| 1540 | | | 1-linoleoyl-GPI (18:2)* | LC/MS neg | 595.28888 | 36594 | | | | 0.82 | 0.78 | 0.0326 | 0.0373 | 1.1479 | 0.8917 |
| 1544 | | | 1-arachidonoyl-GPI (20:4)* | LC/MS neg | 619.28888 | 34214 | | HMDB61690 | | 0.91 | 0.84 | 0.1368 | 0.1070 | 1.0692 | 0.8930 |
| 1555 | | | 1-arachidonoyl-GPA (20:4) | LC/MS neg | 457.23606 | 46325 | | | | 0.68 | 0.59 | 0.0070 | 0.0134 | 1.3556 | 0.7951 |
| 1560 | | | 1-stearoyl-GPG (18:0) | LC/MS neg | 511.30414 | 34437 | | | | 1.02 | 0.79 | 0.2733 | 0.1746 | 0.8553 | 0.6729 |
| 1595 | | Glycerolipid Metabolism | glycerol | LC/MS neg | 91.04007 | 15122 | C00116 | HMDB00131 | 753 | 0.67 | 0.71 | 0.0024 | 0.0067 | 1.3120 | 0.9366 |
| 1596 | | | glycerol 3-phosphate | GC/MS | 357.1 | 15365 | C00093 | HMDB00126 | 754 | 1.22 | 0.59 | 0.0745 | 0.0694 | 3.5547 | 2.1042 |
| 1602 | | Monoacylglycerol | 1-myristoylglycerol (14:0) | LC/MS neg | 227.20163 | 35625 | C01885 | HMDB11561 | 79050 | 1.18 | 1.02 | 0.9995 | 0.3961 | 1.1593 | 1.1828 |
| 1606 | | | 1-palmitoylglycerol (16:0) | LC/MS neg | 255.23293 | 21127 | | HMDB31074 | 14900 | 1.50 | 1.11 | 0.7831 | 0.3409 | 1.1446 | 1.2650 |
| 1607 | | | 2-palmitoylglycerol (16:0) | LC/MS neg | 255.23293 | 33419 | | HMDB11533 | 123409 | 1.13 | 0.55 | 0.1293 | 0.1024 | 1.0680 | 0.5908 |
| 1611 | | | 1-oleoylglycerol (18:1) | LC/MS pos | 374.32649 | 21184 | | HMDB11567 | 5283468 | 1.58 | 1.57 | 0.1875 | 0.1335 | 1.0427 | 1.6322 |
| 1612 | | | 2-oleoylglycerol (18:1) | LC/MS neg | 281.24858 | 21232 | | | 5319879 | 2.48 | 1.09 | 0.9940 | 0.3961 | 0.9554 | 1.0369 |
| 1613 | | | 1-linoleoylglycerol (18:2) | LC/MS neg | 279.23293 | 27447 | | | 5283469 | 1.36 | 1.16 | 0.2659 | 0.1715 | 0.8844 | 1.0280 |
| 1614 | | | 2-linoleoylglycerol (18:2) | LC/MS neg | 279.23293 | 32506 | | HMDB11538 | 5365676 | 1.41 | 1.07 | 0.7523 | 0.3317 | 0.9901 | 1.0612 |
| 1615 | | | 1-linolenoylglycerol (18:3) | LC/MS neg | 277.21728 | 34393 | | HMDB11569 | 53480978 | 1.57 | 1.12 | 0.7006 | 0.3212 | 1.0108 | 1.1313 |
| 1616 | | | 1-arachidonylglycerol (20:4) | LC/MS neg | 303.23293 | 34397 | C13857 | HMDB11572 | 5282281 | 1.48 | 1.08 | 0.7480 | 0.3314 | 1.2854 | 1.3867 |
| 1617 | | | 2-arachidonoylglycerol (20:4) | LC/MS neg | 303.23293 | 19266 | C13856 | HMDB04666 | 5282280 | 2.65 | 1.50 | 0.1823 | 0.1302 | 0.7387 | 1.1068 |
| 1621 | | | 1-docosahexaenoylglycerol (22:6) | LC/MS neg | 309.22236 | 35153 | | HMDB11587 | | 1.45 | 1.07 | 0.6735 | 0.3127 | 1.4517 | 1.5595 |
| 1623 | | | 1-dihomo-linolenylglycerol (alpha, gamma) | LC/MS neg | 305.24858 | 48341 | | | | 1.58 | 0.98 | 0.7840 | 0.3409 | 1.2670 | 1.2413 |
| 1624 | | | oleoyl-linoleoyl-glycerol | LC/MS polar | 663.52055 | 48424 | | | | 1.86 | 0.63 | 0.3136 | 0.1901 | 0.9718 | 0.6148 |
| 1625 | | | 2-docosahexaenoylglcyerol* | LC/MS neg | 309.22236 | 48675 | | HMDB11557 | | 1.13 | 0.85 | 0.4065 | 0.2255 | 1.4629 | 1.2405 |
| 1626 | | | 1-palmitoleoylglycerol (16:1)* | LC/MS neg | 253.21727 | 52431 | | | | 1.72 | 1.01 | 0.9970 | 0.3961 | 1.1795 | 1.1964 |
| 1627 | | | 2-palmitoleoylglycerol (16:1)* | LC/MS neg | 253.21727 | 52432 | | | | 1.33 | 1.03 | 0.8113 | 0.3473 | 0.8085 | 0.8366 |
| 1638 | | Sphingolipid Metabolism | palmitoyl sphingomyelin (d18:1/16:0) | LC/MS polar | 747.56577 | 37506 | | | 9939941 | 0.94 | 0.94 | 0.1546 | 0.1167 | 1.0367 | 0.9742 |
| 1639 | | | stearoyl sphingomyelin (d18:1/18:0) | LC/MS polar | 775.59707 | 19503 | C00550 | HMDB01348 | 6453725 | 0.99 | 0.97 | 0.6438 | 0.3051 | 0.9985 | 0.9697 |
| 1640 | | | sphingomyelin (d18:1/18:1, d18:2/18:0) | LC/MS polar | 773.58142 | 37529 | | | 6443882 | 0.95 | 0.92 | 0.2148 | 0.1462 | 1.1196 | 1.0306 |
| 1643 | | | sphingosine 1-phosphate | LC/MS polar | 378.24148 | 34445 | C06124 | HMDB00277 | 5283560 | 0.84 | 0.82 | 0.0078 | 0.0144 | 1.0986 | 0.9009 |
| 1654 | | | sphingomyelin (d18:1/14:0, d16:1/16:0)* | LC/MS polar | 719.53447 | 42463 | | | 11433862 | 0.87 | 0.88 | 0.0155 | 0.0234 | 1.0413 | 0.9144 |
| 1655 | | | sphingomyelin (d18:2/14:0, d18:1/14:1)* | LC/MS polar | 717.51882 | 47154 | | | | 0.78 | 0.77 | 0.0027 | 0.0072 | 1.1159 | 0.8560 |
| 1656 | | | sphingomyelin (d18:1/24:1, d18:2/24:0)* | LC/MS polar | 857.67533 | 47153 | | | | 1.05 | 1.04 | 0.5966 | 0.2892 | 0.9530 | 0.9938 |
| 1657 | | | sphingomyelin (d18:2/16:0, d18:1/16:1)* | LC/MS polar | 745.55012 | 42459 | | | | 0.86 | 0.86 | 0.0025 | 0.0068 | 1.0752 | 0.9201 |
| 1658 | | | sphingomyelin (d18:1/20:1, d18:2/20:0)* | LC/MS polar | 801.61272 | 48491 | | | | 0.95 | 0.93 | 0.2481 | 0.1625 | 1.0335 | 0.9604 |
| 1659 | | | behenoyl sphingomyelin (d18:1/22:0)* | LC/MS polar | 831.65967 | 48492 | | | | 1.07 | 1.00 | 0.9536 | 0.3856 | 0.9538 | 0.9550 |
| 1660 | | | sphingomyelin (d18:1/22:1, d18:2/22:0, d16:1/24:1)* | LC/MS polar | 829.64402 | 48493 | | | | 0.96 | 0.95 | 0.2966 | 0.1838 | 1.0023 | 0.9560 |
| 1661 | | | sphingomyelin (d18:1/20:0, d16:1/22:0)* | LC/MS polar | 803.62837 | 48490 | | | | 1.04 | 1.00 | 0.9156 | 0.3750 | 1.0199 | 1.0157 |
| 1664 | | Mevalonate Metabolism | 3-hydroxy-3-methylglutarate | LC/MS polar | 161.04555 | 531 | C03761 | HMDB00355 | 1662 | 0.85 | 0.80 | 0.0205 | 0.0275 | 1.1788 | 0.9375 |
| 1674 | | Sterol | lathosterol | GC/MS | 255.2 | 39864 | C01189 | HMDB01170 | 65728 | 0.99 | 0.91 | 0.5099 | 0.2633 | 1.0795 | 0.9857 |
| 1676 | | | cholesterol | GC/MS | 329.3 | 63 | C00187 | HMDB00067 | 11025495 | 0.82 | 0.82 | 0.0000 | 0.0001 | 1.1012 | 0.9015 |
| 1686 | | | 7-alpha-hydroxy-3-oxo-4-cholestenoate (7-Hoca) | LC/MS neg | 429.30103 | 36776 | C17337 | HMDB12458 | 3081085 | 1.11 | 1.10 | 0.0770 | 0.0707 | 0.9698 | 1.0627 |
| 1699 | | | campesterol | GC/MS | 343.4 | 39511 | C01789 | HMDB02869 | 173183 | 0.87 | 0.81 | 0.0241 | 0.0298 | 1.1340 | 0.9240 |
| 1720 | | Steroid | pregnenolone sulfate | LC/MS neg | 395.18976 | 38170 | | HMDB00774 | 105074 | 0.90 | 0.92 | 0.1245 | 0.0995 | 1.4959 | 1.3824 |
| 1728 | | | 21-hydroxypregnenolone disulfate | LC/MS neg | 245.06711 | 46115 | C05485 | HMDB04026 | 134595 | 1.02 | 1.01 | 0.8085 | 0.3470 | 1.1006 | 1.1098 |
| 1736 | | | 5-pregnen-3b, 17-diol-20-one 3-sulfate | LC/MS neg | 411.18468 | 37482 | | HMDB00416 | 152971 | 1.17 | 1.23 | 0.1142 | 0.0937 | 0.6391 | 0.7846 |
| 1738 | | | 5alpha-pregnan-3beta,20beta-diol monosulfate (1) | LC/MS neg | 399.22106 | 37196 | | | | 1.11 | 1.03 | 0.3774 | 0.2122 | 1.0087 | 1.0414 |
| 1741 | | | 5alpha-pregnan-3beta,20alpha-diol monosulfate (2) | LC/MS neg | 399.22106 | 37200 | | | | 1.16 | 1.11 | 0.3471 | 0.2000 | 1.0967 | 1.2166 |
| 1744 | | | 5alpha-pregnan-3beta,20alpha-diol disulfate | LC/MS neg | 239.0853 | 37198 | | | | 1.25 | 1.13 | 0.9809 | 0.3922 | 0.9300 | 1.0531 |
| 1745 | | | 5alpha-pregnan-3(alpha or beta),20beta-diol disulfate | LC/MS neg | 239.0853 | 46172 | | | 5127902 | 1.04 | 0.85 | 0.4829 | 0.2540 | 1.0012 | 0.8517 |
| 1747 | | | pregnen-diol disulfate* | LC/MS neg | 238.07747 | 32562 | | | | 1.04 | 1.02 | 0.8627 | 0.3620 | 1.0242 | 1.0481 |
| 1748 | | | pregn steroid monosulfate* | LC/MS neg | 397.20541 | 32619 | | | | 1.09 | 1.05 | 0.1978 | 0.1393 | 1.2464 | 1.3148 |
| 1749 | | | pregnanediol-3-glucuronide | LC/MS neg | 495.29634 | 40708 | | | 123796 | 1.14 | 1.12 | 0.6403 | 0.3040 | 0.9874 | 1.1021 |
| 1757 | | | cortisol | LC/MS neg | 361.20204 | 1712 | C00735 | HMDB00063 | 5754 | 0.99 | 0.96 | 0.4397 | 0.2372 | 0.9891 | 0.9510 |
| 1759 | | | corticosterone | LC/MS neg | 345.20713 | 5983 | C02140 | HMDB01547 | 5753 | 1.48 | 1.10 | 0.9035 | 0.3712 | 0.8957 | 0.9842 |
| 1762 | | | cortisone | LC/MS neg | 359.18639 | 1769 | C00762 | HMDB02802 | 222786 | 0.85 | 0.82 | 0.0138 | 0.0220 | 1.1471 | 0.9461 |
| 1767 | | | dehydroisoandrosterone sulfate (DHEA-S) | LC/MS neg | 367.15846 | 32425 | C04555 | HMDB01032 | 12594 | 0.97 | 0.93 | 0.3141 | 0.1901 | 1.1466 | 1.0715 |
| 1768 | | | 16a-hydroxy DHEA 3-sulfate | LC/MS neg | 383.15338 | 38168 | | | | 0.97 | 0.94 | 0.3431 | 0.1981 | 1.0935 | 1.0309 |
| 1770 | | | epiandrosterone sulfate | LC/MS neg | 369.17411 | 33973 | C07635 | HMDB00365 | | 1.01 | 1.00 | 0.7441 | 0.3309 | 1.0730 | 1.0727 |
| 1773 | | | androsterone sulfate | LC/MS neg | 369.17411 | 31591 | | HMDB02759 | 159663 | 1.08 | 1.02 | 0.2394 | 0.1580 | 1.2290 | 1.2593 |
| 1774 | | | 4-androsten-3beta,17beta-diol monosulfate (1) | LC/MS neg | 369.17411 | 37211 | | HMDB03818 | | 1.22 | 1.18 | 0.0755 | 0.0696 | 1.0317 | 1.2170 |
| 1775 | | | 4-androsten-3beta,17beta-diol monosulfate (2) | LC/MS neg | 369.17411 | 37210 | | | | 0.84 | 0.75 | 0.0668 | 0.0654 | 1.2791 | 0.9582 |
| 1778 | | | 4-androsten-3alpha,17alpha-diol monosulfate (2) | LC/MS neg | 369.17411 | 37207 | | | | 1.10 | 1.10 | 0.9967 | 0.3961 | 1.3812 | 1.5208 |
| 1779 | | | 4-androsten-3alpha,17alpha-diol monosulfate (3) | LC/MS neg | 369.17411 | 37209 | | | | 1.09 | 1.08 | 0.2557 | 0.1658 | 1.1219 | 1.2077 |
| 1783 | | | 4-androsten-3beta,17beta-diol disulfate (1) | LC/MS neg | 224.06182 | 37202 | C04295 | HMDB03818 | 10634 | 0.94 | 0.94 | 0.0997 | 0.0852 | 1.0630 | 0.9950 |
| 1784 | | | 4-androsten-3beta,17beta-diol disulfate (2) | LC/MS neg | 224.0624 | 37203 | C04295 | HMDB03818 | 10634 | 0.79 | 0.82 | 0.0203 | 0.0274 | 1.2465 | 1.0254 |
| 1788 | | | 5alpha-androstan-3alpha,17beta-diol monosulfate (1) | LC/MS neg | 371.18976 | 37186 | | | | 1.32 | 1.26 | 0.0026 | 0.0069 | 1.1409 | 1.4394 |
| 1789 | | | 5alpha-androstan-3alpha,17alpha-diol monosulfate | LC/MS neg | 371.18976 | 37183 | | | | 1.34 | 1.06 | 0.3260 | 0.1944 | 0.7442 | 0.7917 |
| 1791 | | | 5alpha-androstan-3beta,17beta-diol monosulfate (2) | LC/MS neg | 371.18976 | 37192 | | | | 1.25 | 1.12 | 0.0569 | 0.0585 | 1.0768 | 1.2012 |
| 1796 | | | 5alpha-androstan-3beta,17alpha-diol disulfate | LC/MS neg | 225.06965 | 37187 | | | | 1.33 | 0.93 | 0.5113 | 0.2635 | 1.0675 | 0.9882 |
| 1797 | | | 5alpha-androstan-3alpha,17beta-diol disulfate | LC/MS neg | 225.06965 | 37184 | | | | 1.07 | 1.03 | 0.6671 | 0.3114 | 0.6755 | 0.6929 |
| 1798 | | | 5alpha-androstan-3beta,17beta-diol disulfate | LC/MS neg | 225.06965 | 37190 | C12525 | HMDB00493 | 242332 | 0.86 | 0.87 | 0.0375 | 0.0413 | 1.4057 | 1.2287 |
| 1803 | | | andro steroid monosulfate (1)* | LC/MS neg | 383.1534 | 32827 | C04555 | HMDB02759 | | 0.93 | 0.90 | 0.1590 | 0.1189 | 1.0725 | 0.9699 |
| 1826 | | | etiocholanolone glucuronide | LC/MS neg | 465.24939 | 47112 | | | | 0.88 | 0.93 | 0.0401 | 0.0434 | 1.4135 | 1.3203 |
| 1828 | | | 5alpha-androstan-3alpha,17beta-diol monosulfate (2) | LC/MS neg | 371.18976 | 37185 | | | | 0.98 | 0.93 | 0.4230 | 0.2311 | 1.3318 | 1.2430 |
| 1829 | | | 17alpha-hydroxypregnanolone glucuronide | LC/MS neg | 509.2756 | 47403 | | | | 1.19 | 1.12 | 0.2941 | 0.1827 | 0.9477 | 1.0567 |
| 1834 | | | pregnanolone/allopregnanolone sulfate | LC/MS neg | 397.20541 | 48394 | | | | 0.79 | 0.56 | 0.0216 | 0.0284 | 1.0092 | 0.5669 |
| 1841 | | Primary Bile Acid Metabolism | cholate | LC/MS neg | 407.28029 | 22842 | C00695 | HMDB00619 | 221493 | 1.20 | 1.12 | 0.2545 | 0.1655 | 2.6735 | 3.0064 |
| 1842 | | | glycocholate | LC/MS neg | 464.30176 | 18476 | C01921 | HMDB00138 | 10140 | 1.73 | 1.07 | 0.2695 | 0.1734 | 1.3238 | 1.4101 |
| 1843 | | | taurocholate | LC/MS neg | 514.28439 | 18497 | C05122 | HMDB00036 | 6675 | 2.02 | 0.81 | 0.3405 | 0.1971 | 1.7445 | 1.4159 |
| 1844 | | | chenodeoxycholate | LC/MS neg | 391.28538 | 1563 | C02528 | HMDB00518 | 10133 | 1.51 | 0.86 | 0.5609 | 0.2821 | 1.2556 | 1.0758 |
| 1845 | | | glycochenodeoxycholate | LC/MS neg | 448.30684 | 32346 | C05466 | HMDB00637 | 12544 | 1.20 | 0.81 | 0.7292 | 0.3283 | 1.5177 | 1.2302 |
| 1846 | | | taurochenodeoxycholate | LC/MS neg | 498.28948 | 18494 | C05465 | HMDB00951 | 387316 | 1.33 | 0.71 | 0.7397 | 0.3303 | 2.1697 | 1.5304 |
| 1850 | | | tauro-beta-muricholate | LC/MS neg | 514.28439 | 33983 | | HMDB00932 | 168408 | 1.49 | 0.84 | 0.7556 | 0.3326 | 1.4203 | 1.1961 |
| 1852 | | Secondary Bile Acid Metabolism | deoxycholate | LC/MS neg | 391.28538 | 1114 | C04483 | HMDB00626 | 222528 | 1.19 | 0.95 | 0.7400 | 0.3303 | 1.2145 | 1.1520 |
| 1853 | | | glycodeoxycholate | LC/MS neg | 448.30684 | 18477 | C05464 | HMDB00631 | 3035026 | 1.79 | 1.44 | 0.0386 | 0.0423 | 0.9031 | 1.3019 |
| 1854 | | | taurodeoxycholate | LC/MS neg | 498.28948 | 12261 | C05463 | HMDB00896 | 2733768 | 1.48 | 0.97 | 0.2592 | 0.1676 | 1.2745 | 1.2357 |
| 1858 | | | glycolithocholate | LC/MS neg | 432.31193 | 31912 | C15557 | HMDB00698 | 115245 | 1.48 | 1.16 | 0.0727 | 0.0682 | 0.8704 | 1.0108 |
| 1859 | | | glycolithocholate sulfate* | LC/MS neg | 255.63073 | 32620 | C11301 | HMDB02639 | 72222 | 1.84 | 1.35 | 0.1176 | 0.0959 | 0.8721 | 1.1776 |
| 1860 | | | taurolithocholate | LC/MS neg | 482.29456 | 31889 | C02592 | HMDB00722 | 10595 | 1.08 | 1.06 | 0.3524 | 0.2017 | 0.8723 | 0.9241 |
| 1861 | | | taurolithocholate 3-sulfate | LC/MS neg | 280.62205 | 36850 | C03642 | HMDB02580 | 440071 | 1.78 | 0.95 | 0.8332 | 0.3532 | 1.3687 | 1.3063 |
| 1863 | | | ursodeoxycholate | LC/MS neg | 391.28538 | 1605 | C07880 | HMDB00946 | 31401 | 0.92 | 0.68 | 0.0694 | 0.0667 | 1.3801 | 0.9317 |
| 1864 | | | glycoursodeoxycholate | LC/MS neg | 448.30684 | 39379 | | HMDB00708 | 12310288 | 1.22 | 0.97 | 0.9978 | 0.3961 | 1.4790 | 1.4325 |
| 1865 | | | tauroursodeoxycholate | LC/MS neg | 498.28948 | 39378 | | HMDB00874 | 9848818 | 1.92 | 0.70 | 0.8530 | 0.3592 | 1.8828 | 1.3244 |
| 1872 | | | glycohyocholate | LC/MS neg | 464.30176 | 42574 | | | | 1.51 | 1.22 | 0.1674 | 0.1230 | 1.0591 | 1.2917 |
| 1881 | | | glycocholenate sulfate* | LC/MS neg | 254.62291 | 32599 | | | | 1.04 | 0.94 | 0.2846 | 0.1777 | 1.1210 | 1.0563 |
| 1882 | | | taurocholenate sulfate | LC/MS neg | 279.61422 | 32807 | | | | 0.91 | 0.76 | 0.0982 | 0.0844 | 1.6493 | 1.2503 |
| 1886 | | | 3b-hydroxy-5-cholenoic acid | LC/MS neg | 373.27482 | 43507 | | HMDB00308 | 92997 | 1.08 | 0.85 | 0.4214 | 0.2307 | 1.1839 | 1.0086 |
| 2430 | Nucleotide | Purine Metabolism, (Hypo)Xanthine/Inosine containing | inosine | LC/MS neg | 267.07349 | 1123 | C00294 | HMDB00195 | 6021 | 0.34 | 0.25 | 0.0000 | 0.0002 | 2.2074 | 0.5410 |
| 2431 | | | hypoxanthine | LC/MS neg | 135.03123 | 3127 | C00262 | HMDB00157 | 790 | 1.22 | 1.16 | 0.4043 | 0.2253 | 0.9047 | 1.0515 |
| 2432 | | | xanthine | LC/MS pos | 153.04071 | 3147 | C00385 | HMDB00292 | 1188 | 1.18 | 1.06 | 0.5955 | 0.2892 | 0.9284 | 0.9849 |
| 2434 | | | xanthosine | LC/MS pos | 285.08296 | 15136 | C01762 | HMDB00299 | 64959 | 1.36 | 1.24 | 0.6936 | 0.3197 | 0.8634 | 1.0725 |
| 2439 | | | urate | LC/MS neg | 167.02106 | 1604 | C00366 | HMDB00289 | 1175 | 1.05 | 1.04 | 0.1231 | 0.0990 | 1.0240 | 1.0686 |
| 2440 | | | allantoin | LC/MS polar | 157.03671 | 1107 | C02350 | HMDB00462 | 204 | 2.22 | 2.13 | 0.0000 | 0.0001 | 0.7023 | 1.4963 |
| 2448 | | Purine Metabolism, Adenine containing | adenosine 3',5'-cyclic monophosphate (cAMP) | LC/MS neg | 328.04524 | 2831 | C00575 | HMDB00058 | 6076 | 0.81 | 0.77 | 0.0217 | 0.0284 | 1.1050 | 0.8499 |
| 2452 | | | adenosine | LC/MS neg | 134.0472 | 555 | C00212 | HMDB00050 | 60961 | 0.78 | 0.29 | 0.0215 | 0.0284 | 3.4216 | 0.9964 |
| 2453 | | | adenine | LC/MS pos | 136.06178 | 554 | C00147 | HMDB00034 | 190 | 0.89 | 0.83 | 0.0811 | 0.0737 | 1.1237 | 0.9381 |
| 2458 | | | N1-methyladenosine | LC/MS pos | 282.11969 | 15650 | C02494 | HMDB03331 | 27476 | 1.14 | 1.15 | 0.8697 | 0.3632 | 0.9297 | 1.0679 |
| 2459 | | | N6-methyladenosine | LC/MS neg | 148.06285 | 37114 | | HMDB04044 | 102175 | 1.45 | 1.40 | 0.0000 | 0.0003 | 0.8416 | 1.1763 |
| 2468 | | | N6-carbamoylthreonyladenosine | LC/MS neg | 411.12698 | 35157 | | HMDB41623 | 161466 | 1.51 | 1.47 | 0.0000 | 0.0001 | 0.8161 | 1.2014 |
| 2477 | | | N6-succinyladenosine | LC/MS neg | 382.10043 | 48130 | | HMDB00912 | | 2.10 | 1.99 | 0.0000 | 0.0002 | 0.7039 | 1.4013 |
| 2485 | | Purine Metabolism, Guanine containing | guanosine | LC/MS pos | 284.09895 | 1573 | C00387 | HMDB00133 | 6802 | 0.31 | 0.22 | 0.0000 | 0.0002 | 2.2781 | 0.5097 |
| 2488 | | | 7-methylguanine | LC/MS pos | 166.07234 | 35114 | C02242 | HMDB00897 | 11361 | 1.09 | 1.09 | 0.5806 | 0.2862 | 0.9617 | 1.0524 |
| 2492 | | | N1-methylguanosine | LC/MS pos | 298.1146 | 31609 | | HMDB01563 | 96373 | 1.16 | 1.15 | 0.0122 | 0.0200 | 0.9447 | 1.0890 |
| 2493 | | | N2-methylguanosine | LC/MS pos | 296.10004 | 35133 | | HMDB05862 | 3035422 | 1.35 | 1.32 | 0.0016 | 0.0051 | 0.8647 | 1.1409 |
| 2494 | | | N2,N2-dimethylguanosine | LC/MS pos | 312.13025 | 35137 | | HMDB04824 | 92919 | 1.28 | 1.25 | 0.0001 | 0.0006 | 0.9081 | 1.1346 |
| 2505 | | Pyrimidine Metabolism, Orotate containing | dihydroorotate | LC/MS polar | 157.02548 | 601 | C00337 | HMDB03349 | 648 | 0.73 | 0.72 | 0.0000 | 0.0000 | 1.2239 | 0.8836 |
| 2506 | | | orotate | LC/MS polar | 155.00983 | 1505 | C00295 | HMDB00226 | 967 | 1.27 | 1.08 | 0.2068 | 0.1418 | 1.0800 | 1.1654 |
| 2508 | | | orotidine | LC/MS polar | 287.05209 | 35172 | C01103 | HMDB00788 | 92751 | 1.89 | 1.90 | 0.0000 | 0.0000 | 0.8196 | 1.5540 |
| 2518 | | Pyrimidine Metabolism, Uracil containing | uridine | LC/MS neg | 243.06226 | 606 | C00299 | HMDB00296 | 6029 | 1.03 | 1.02 | 0.6472 | 0.3061 | 1.0040 | 1.0221 |
| 2519 | | | uracil | LC/MS neg | 111.02 | 605 | C00106 | HMDB00300 | 1174 | 1.34 | 1.30 | 0.0139 | 0.0220 | 0.9029 | 1.1707 |
| 2520 | | | pseudouridine | LC/MS pos | 245.07682 | 33442 | C02067 | HMDB00767 | 15047 | 1.26 | 1.26 | 0.0000 | 0.0000 | 0.9344 | 1.1812 |
| 2521 | | | 5-methyluridine (ribothymidine) | LC/MS neg | 257.07791 | 35136 | | HMDB00884 | 445408 | 0.84 | 0.83 | 0.0228 | 0.0289 | 1.0719 | 0.8916 |
| 2528 | | | 5,6-dihydrouracil | LC/MS pos | 115.05021 | 1559 | C00429 | HMDB00076 | 649 | 1.18 | 1.19 | 0.0071 | 0.0134 | 0.9634 | 1.1445 |
| 2532 | | | 2'-deoxyuridine | LC/MS neg | 111.01997 | 1412 | C00526 | HMDB00012 | 13712 | 1.54 | 1.44 | 0.0105 | 0.0176 | 0.8843 | 1.2728 |
| 2533 | | | 4-ureidobutyrate | LC/MS polar | 145.06186 | 22118 | | | 1571307 | 0.72 | 0.71 | 0.0025 | 0.0068 | 1.2137 | 0.8620 |
| 2534 | | | 3-ureidopropionate | LC/MS pos | 133.06077 | 3155 | C02642 | HMDB00026 | 111 | 1.75 | 1.64 | 0.0033 | 0.0077 | 0.8271 | 1.3524 |
| 2535 | | | beta-alanine | GC/MS | 334.1 | 35838 | C00099 | HMDB00056 | 239 | 1.27 | 1.04 | 0.6052 | 0.2922 | 1.0703 | 1.1088 |
| 2536 | | | N-acetyl-beta-alanine | LC/MS pos | 132.06552 | 37432 | C01073 | | 76406 | 1.02 | 0.98 | 0.8902 | 0.3677 | 1.1099 | 1.0896 |
| 2544 | | Pyrimidine Metabolism, Cytidine containing | cytidine | LC/MS neg | 242.07824 | 514 | C00475 | HMDB00089 | 6175 | 1.03 | 0.73 | 0.3225 | 0.1933 | 1.4520 | 1.0532 |
| 2548 | | | N4-acetylcytidine | LC/MS pos | 286.10337 | 35130 | | HMDB05923 | 107461 | 1.46 | 1.45 | 0.0002 | 0.0013 | 0.9307 | 1.3468 |
| 2562 | | Pyrimidine Metabolism, Thymine containing | 5,6-dihydrothymine | LC/MS pos | 129.06586 | 1418 | C00906 | HMDB00079 | 93556 | 0.90 | 0.88 | 0.0532 | 0.0560 | 1.1117 | 0.9749 |
| 2566 | Cofactors and Vitamins | Nicotinate and Nicotinamide Metabolism | quinolinate | LC/MS polar | 166.01458 | 1899 | C03722 | HMDB00232 | 1066 | 0.98 | 0.94 | 0.4144 | 0.2277 | 0.9752 | 0.9195 |
| 2570 | | | nicotinamide | LC/MS pos | 123.05529 | 594 | C00153 | HMDB01406 | 936 | 1.40 | 1.17 | 0.8360 | 0.3538 | 0.9529 | 1.1141 |
| 2582 | | | 1-methylnicotinamide | LC/MS pos | 137.07094 | 27665 | C02918 | HMDB00699 | 10129985 | 0.66 | 0.56 | 0.0006 | 0.0025 | 1.2096 | 0.6797 |
| 2587 | | | trigonelline (N'-methylnicotinate) | LC/MS pos | 138.05496 | 32401 | C01004 | HMDB00875 | 5570 | 1.00 | 0.70 | 0.2016 | 0.1398 | 1.8939 | 1.3334 |
| 2589 | | | N1-Methyl-2-pyridone-5-carboxamide | LC/MS pos | 153.06586 | 40469 | C05842 | HMDB04193 | 69698 | 0.67 | 0.62 | 0.0010 | 0.0035 | 1.2544 | 0.7773 |
| 2595 | | Pantothenate and CoA Metabolism | pantothenate | LC/MS neg | 218.10339 | 1508 | C00864 | HMDB00210 | 6613 | 1.10 | 1.02 | 0.4289 | 0.2323 | 1.0451 | 1.0701 |
| 2603 | | Ascorbate and Aldarate Metabolism | ascorbate (Vitamin C) | GC/MS | 332.1 | 1640 | C00072 | HMDB00044 | | 2.05 | 1.59 | 0.8849 | 0.3677 | 1.1762 | 1.8725 |
| 2605 | | | threonate | LC/MS polar | 135.02989 | 27738 | C01620 | HMDB00943 | 151152 | 0.78 | 0.76 | 0.0012 | 0.0041 | 1.0236 | 0.7782 |
| 2606 | | | arabonate | GC/MS | 292.1 | 37516 | C00878 | HMDB00539 | 122045 | 1.03 | 0.96 | 0.8671 | 0.3627 | 1.0812 | 1.0392 |
| 2607 | | | oxalate (ethanedioate) | LC/MS neg | 88.98803 | 20694 | C00209 | HMDB02329 | 971 | 0.65 | 0.64 | 0.0166 | 0.0244 | 1.1510 | 0.7406 |
| 2608 | | | gulonic acid* | LC/MS polar | 195.05102 | 46957 | | | 9794176 | 2.06 | 1.75 | 0.0001 | 0.0008 | 0.6970 | 1.2204 |
| 2609 | | Tocopherol Metabolism | alpha-tocopherol | GC/MS | 502.5 | 1561 | C02477 | HMDB01893 | 14985 | 0.82 | 0.81 | 0.0005 | 0.0023 | 1.0892 | 0.8851 |
| 2611 | | | beta-tocopherol | GC/MS | 488.3 | 35702 | C14152 | HMDB06335 | 6857447 | 1.15 | 0.82 | 0.5367 | 0.2733 | 1.0853 | 0.8921 |
| 2614 | | | gamma-tocopherol | GC/MS | 488.4 | 33420 | C02483 | HMDB01492 | 14986 | 0.82 | 0.66 | 0.0359 | 0.0401 | 1.4611 | 0.9659 |
| 2617 | | | gamma-CEHC | LC/MS neg | 263.12888 | 44876 | | HMDB01931 | 133098 | 0.89 | 0.76 | 0.0785 | 0.0718 | 1.3059 | 0.9880 |
| 2618 | | | gamma-CEHC glucuronide* | LC/MS neg | 439.16097 | 42381 | | | | 1.24 | 0.93 | 0.4958 | 0.2587 | 0.9912 | 0.9227 |
| 2620 | | | alpha-CEHC sulfate | LC/MS neg | 357.10134 | 47666 | | | | 1.46 | 1.25 | 0.4440 | 0.2384 | 0.9307 | 1.1635 |
| 2644 | | | bilirubin (Z,Z) | LC/MS neg | 583.2562 | 43807 | C00486 | HMDB00054 | 5280352 | 0.56 | 0.51 | 0.0001 | 0.0008 | 1.7259 | 0.8726 |

**Comparison mean values significantly different:**

- (red) $p \le 0.05$, fold of change ≥ 1.00
- (dark green) $p \le 0.05$, fold of change < 1.00

**Comparison mean value difference approaching significance:**

- (pink) $0.05 < p < 0.10$, fold of change ≥ 1.00
- (light green) $0.05 < p < 0.10$, fold of change < 1.00

| Pathway Sort Order | Super Pathway | Sub Pathway | Biochemical Name | Platform | Mass | Comp ID | KEGG | HMDB | PubChem | Fold of Change, Paired t-Test: Post G-CSF / Pre G-CSF: Mean Pairs Ratio | Fold of Change, Paired t-Test: Post G-CSF / Pre G-CSF: Group Mean Ratio | Paired t-Test: Post G-CSF / Pre G-CSF: *p*-value | Paired t-Test: Post G-CSF / Pre G-CSF: *q*-value | Mean Values: Pre G-CSF | Mean Values: Post G-CSF |
|---|---|---|---|---|---|---|---|---|---|---|---|---|---|---|---|
| 2645 | | Hemoglobin and Porphyrin Metabolism | bilirubin (E,E)* | LC/MS neg | 583.2562 | 32586 | | | 5315454 | 0.74 | 0.69 | 0.0042 | 0.0091 | 1.3280 | 0.9198 |
| 2647 | | | biliverdin | LC/MS neg | 581.24056 | 2137 | C00500 | HMDB01008 | 5353439 | 0.86 | 0.75 | 0.0695 | 0.0667 | 1.4135 | 1.0622 |
| 2648 | | | I-urobilinogen | LC/MS neg | 591.31881 | 32426 | C05790 | HMDB04157 | 26818 | 1.38 | 0.63 | 0.5673 | 0.2837 | 1.3546 | 0.8574 |
| 2670 | | Vitamin B6 Metabolism | pyridoxate | LC/MS neg | 182.04588 | 31555 | C00847 | HMDB00017 | 6723 | 0.77 | 0.70 | 0.0021 | 0.0061 | 1.4754 | 1.0353 |
| 2672 | Xenobiotics | Benzoate Metabolism | hippurate | LC/MS neg | 178.05096 | 15753 | C01586 | HMDB00714 | 464 | 1.23 | 0.67 | 0.2450 | 0.1609 | 1.8526 | 1.2397 |
| 2676 | | | 2-hydroxyhippurate (salicylurate) | LC/MS neg | 194.04588 | 18281 | C07588 | HMDB00840 | 10253 | 1.45 | 0.72 | 0.7075 | 0.3228 | 1.4091 | 1.0133 |
| 2677 | | | 3-hydroxyhippurate | LC/MS neg | 194.04588 | 39600 | | HMDB06116 | 450268 | 0.96 | 0.67 | 0.0596 | 0.0599 | 1.2752 | 0.8492 |
| 2678 | | | 4-hydroxyhippurate | LC/MS neg | 194.04588 | 35527 | | HMDB13678 | 151012 | 1.56 | 1.22 | 0.8892 | 0.3677 | 1.4274 | 1.7445 |
| 2684 | | | benzoate | LC/MS neg | 121.0295 | 15778 | C00180 | HMDB01870 | 243 | 1.01 | 0.99 | 0.8878 | 0.3677 | 0.9957 | 0.9902 |
| 2693 | | | 4-hydroxybenzoate | LC/MS neg | 137.02441 | 21133 | C00156 | HMDB00500 | 135 | 1.93 | 1.72 | 0.1819 | 0.1302 | 0.9442 | 1.6219 |
| 2701 | | | catechol sulfate | LC/MS neg | 188.98631 | 35320 | C00090 | HMDB59724 | 3083879 | 0.88 | 0.71 | 0.0743 | 0.0694 | 1.2579 | 0.8891 |
| 2703 | | | O-methylcatechol sulfate | LC/MS neg | 203.00196 | 46111 | | | 22473 | 0.94 | 0.72 | 0.1158 | 0.0947 | 1.6539 | 1.1962 |
| 2704 | | | 3-methyl catechol sulfate (1) | LC/MS neg | 203.00196 | 46165 | | | | 0.77 | 0.53 | 0.0229 | 0.0289 | 1.4830 | 0.7879 |
| 2705 | | | 3-methyl catechol sulfate (2) | LC/MS neg | 203.00196 | 46164 | | | | 0.74 | 0.52 | 0.0158 | 0.0234 | 1.3245 | 0.6851 |
| 2706 | | | 4-methylcatechol sulfate | LC/MS neg | 203.00196 | 46146 | | | | 1.40 | 1.00 | 0.3081 | 0.1878 | 1.4099 | 1.4078 |
| 2710 | | | methyl-4-hydroxybenzoate | LC/MS polar | 151.04006 | 34386 | D01400 | HMDB32572 | 7456 | 49.34 | 30.31 | 0.0363 | 0.0404 | 0.9339 | 28.3049 |
| 2714 | | | 2-ethylphenylsulfate | LC/MS neg | 201.0227 | 36847 | | | | 1.67 | 0.93 | 0.9712 | 0.3895 | 1.9349 | 1.8018 |
| 2715 | | | 3-ethylphenylsulfate | LC/MS neg | 201.0227 | 36848 | C14386 | | | 3.11 | 0.80 | 0.3012 | 0.1860 | 1.8728 | 1.4896 |
| 2716 | | | 4-ethylphenylsulfate | LC/MS neg | 201.0227 | 36099 | C13637 | | | 1.43 | 0.78 | 0.9712 | 0.3895 | 1.6244 | 1.2733 |
| 2717 | | | 4-vinylphenol sulfate | LC/MS neg | 199.00705 | 36098 | C05627 | HMDB04072 | 6426766 | 0.73 | 0.71 | 0.0116 | 0.0191 | 3.2346 | 2.3007 |
| 2729 | | | 3-methoxycatechol sulfate (1) | LC/MS neg | 218.99688 | 48763 | | | | 1.06 | 0.93 | 0.5731 | 0.2849 | 0.8722 | 0.8089 |
| 2730 | | | 3-methoxycatechol sulfate (2) | LC/MS neg | 218.99688 | 48752 | | | | 1.03 | 0.78 | 0.1419 | 0.1094 | 1.7490 | 1.3576 |
| 2731 | | | methyl-4-hydroxybenzoate sulfate | LC/MS neg | 230.99688 | 48429 | | | | 45.71 | 2.93 | 0.3213 | 0.1930 | 2.5108 | 7.3622 |
| 2732 | | | propyl 4-hydroxybenzoate sulfate | LC/MS neg | 259.02818 | 48460 | | | | 2.04 | 1.83 | 0.0009 | 0.0034 | 0.6908 | 1.2675 |
| 2735 | | Xanthine Metabolism | caffeine | LC/MS pos | 195.08766 | 569 | C07481 | HMDB01847 | 2519 | 1.11 | 0.80 | 0.7071 | 0.3228 | 1.2373 | 0.9898 |
| 2736 | | | paraxanthine | LC/MS pos | 181.07201 | 18254 | C13747 | HMDB01860 | 4687 | 1.09 | 0.81 | 0.5850 | 0.2872 | 1.2918 | 1.0507 |
| 2737 | | | theobromine | LC/MS pos | 181.07201 | 18392 | C07480 | HMDB02825 | 5429 | 1.12 | 0.88 | 0.3978 | 0.2221 | 1.2205 | 1.0723 |
| 2738 | | | theophylline | LC/MS pos | 181.07201 | 18394 | C07130 | HMDB01889 | 2153 | 1.08 | 0.87 | 0.5598 | 0.2821 | 1.2923 | 1.1239 |
| 2739 | | | 1-methylurate | LC/MS pos | 183.05127 | 34395 | C16359 | HMDB03099 | 69726 | 0.87 | 0.63 | 0.0575 | 0.0588 | 1.5026 | 0.9466 |
| 2741 | | | 1,3-dimethylurate | LC/MS neg | 195.05236 | 32391 | | HMDB01857 | 70346 | 0.90 | 0.78 | 0.1199 | 0.0970 | 1.3652 | 1.0687 |
| 2742 | | | 1,7-dimethylurate | LC/MS neg | 195.05236 | 34400 | C16356 | HMDB11103 | 91611 | 1.08 | 0.82 | 0.3272 | 0.1945 | 1.1517 | 0.9452 |
| 2743 | | | 3,7-dimethylurate | LC/MS neg | 195.05236 | 34399 | C16360 | HMDB01982 | 83126 | 0.73 | 0.63 | 0.0085 | 0.0155 | 1.7071 | 1.0823 |
| 2744 | | | 1,3,7-trimethylurate | LC/MS neg | 209.06801 | 34404 | C16361 | HMDB02123 | 79437 | 0.98 | 0.83 | 0.3356 | 0.1955 | 1.3519 | 1.1184 |
| 2745 | | | 1-methylxanthine | LC/MS pos | 167.05636 | 34389 | C16358 | HMDB10738 | 80220 | 0.68 | 0.55 | 0.0041 | 0.0089 | 1.7840 | 0.9756 |
| 2746 | | | 3-methylxanthine | LC/MS pos | 167.05636 | 32445 | C16357 | HMDB01886 | 70639 | 0.97 | 0.80 | 0.1759 | 0.1280 | 1.2181 | 0.9687 |
| 2747 | | | 7-methylxanthine | LC/MS pos | 167.05636 | 34390 | C16353 | HMDB01991 | 68374 | 0.81 | 0.61 | 0.0290 | 0.0340 | 1.4468 | 0.8764 |
| 2748 | | | 5-acetylamino-6-amino-3-methyluracil | LC/MS pos | 199.08257 | 34424 | C16366 | HMDB04400 | 88299 | 1.48 | 0.52 | 0.2326 | 0.1552 | 1.7199 | 0.8979 |
| 2749 | | | 5-acetylamino-6-formylamino-3-methyluracil | LC/MS pos | 227.07748 | 34401 | C16365 | HMDB11105 | 108214 | 0.80 | 0.51 | 0.0304 | 0.0350 | 1.2637 | 0.6425 |
| 2751 | | Tobacco Metabolite | cotinine | LC/MS pos | 177.10224 | 553 | | HMDB01046 | 854019 | 0.91 | 0.80 | 0.2006 | 0.1398 | 4.9467 | 3.9568 |
| 2752 | | | hydroxycotinine | LC/MS pos | 193.09716 | 38661 | | HMDB01390 | 10219774 | 0.93 | 0.82 | 0.3037 | 0.1860 | 0.1734 | 0.1420 |
| 2753 | | | cotinine N-oxide | LC/MS pos | 193.09716 | 38662 | | HMDB01411 | 9815514 | 1.00 | 1.00 | 0.3343 | 0.1953 | 0.9699 | 0.9653 |
| 2761 | | Food Component/Plant | 2-piperidinone | LC/MS pos | 100.07569 | 43400 | | | 12665 | 0.73 | 0.67 | 0.0012 | 0.0041 | 1.4596 | 0.9842 |
| 2833 | | | levulinate (4-oxovalerate) | LC/MS pos | 99.04406 | 22177 | | HMDB00720 | 11579 | 0.94 | 0.89 | 0.2054 | 0.1415 | 1.1401 | 1.0113 |
| 2844 | | | 1,6-anhydroglucose | GC/MS | 204.1 | 21049 | | HMDB00640 | 2724705 | 1.77 | 1.45 | 0.3694 | 0.2090 | 1.1835 | 1.7115 |
| 2847 | | | 2,3-dihydroxyisovalerate | LC/MS polar | 133.05063 | 38276 | C04039 | HMDB12141 | 677 | 1.17 | 0.92 | 0.7490 | 0.3314 | 1.3236 | 1.2239 |
| 2853 | | | 2-isopropylmalate | LC/MS polar | 175.06119 | 15667 | C02504 | HMDB00402 | 77 | 1.33 | 0.91 | 0.8870 | 0.3677 | 1.1364 | 1.0373 |
| 2864 | | | 3-hydroxyindolin-2-one | LC/MS pos | 132.0444 | 42561 | C11130 | | 6097 | 1.32 | 1.17 | 0.2768 | 0.1754 | 1.0098 | 1.1811 |
| 2871 | | | betonicine | LC/MS pos | 160.09682 | 38100 | C08269 | HMDB29412 | 164642 | 2.13 | 0.71 | 0.5291 | 0.2715 | 4.4510 | 3.1706 |
| 2875 | | | gluconate | LC/MS polar | 195.05102 | 587 | C00257 | HMDB00625 | 10690 | 1.09 | 1.04 | 0.7001 | 0.3212 | 1.0182 | 1.0584 |
| 2885 | | | N-acetylalliin | LC/MS neg | 218.04925 | 45404 | | | | 1.56 | 0.87 | 0.6664 | 0.3114 | 0.7451 | 0.6463 |
| 2915 | | | cinnamoylglycine | LC/MS neg | 204.06661 | 38637 | | HMDB11621 | 709625 | 0.86 | 0.67 | 0.0692 | 0.0667 | 1.8104 | 1.2175 |
| 2932 | | | dihydroferulic acid | LC/MS neg | 195.06628 | 40481 | | | 14340 | 0.97 | 0.76 | 0.0909 | 0.0796 | 1.6383 | 1.2442 |
| 2941 | | | ergothioneine | LC/MS neg | 228.08122 | 37459 | C05570 | HMDB03045 | 3032311 | 0.46 | 0.51 | 0.0000 | 0.0000 | 1.8383 | 0.9406 |
| 2943 | | | erythritol | GC/MS | 217 | 20699 | C00503 | HMDB02994 | 222285 | 1.13 | 1.11 | 0.0833 | 0.0743 | 0.9812 | 1.0929 |
| 2946 | | | ferulic acid 4-sulfate | LC/MS neg | 273.00744 | 47114 | | HMDB29200 | 6305574 | 0.87 | 0.56 | 0.0747 | 0.0694 | 1.8641 | 1.0487 |
| 2965 | | | homostachydrine* | LC/MS pos | 158.11756 | 33009 | C08283 | HMDB33433 | 441447 | 0.97 | 0.82 | 0.3027 | 0.1860 | 1.1571 | 0.9504 |
| 2969 | | | indolin-2-one | LC/MS pos | 134.06004 | 43374 | C12312 | | 321710 | 1.34 | 0.94 | 0.8925 | 0.3679 | 1.1563 | 1.0841 |
| 2991 | | | methyl indole-3-acetate | LC/MS pos | 190.08626 | 1584 | | HMDB29738 | 74706 | 1.11 | 1.01 | 0.8905 | 0.3677 | 1.4215 | 1.4291 |
| 2994 | | | N-(2-furoyl)glycine | LC/MS neg | 168.03023 | 31536 | | HMDB00439 | 21863 | 0.91 | 0.41 | 0.0411 | 0.0443 | 2.3108 | 0.9588 |
| 3017 | | | piperine | LC/MS pos | 286.14377 | 33935 | C03882 | HMDB29377 | 638024 | 1.06 | 0.87 | 0.2148 | 0.1462 | 1.5315 | 1.3378 |
| 3027 | | | quinate | LC/MS polar | 191.05611 | 18335 | C00296 | HMDB03072 | 6508 | 1.11 | 0.68 | 0.5945 | 0.2892 | 1.3111 | 0.8937 |
| 3031 | | | saccharin | LC/MS neg | 181.99173 | 21151 | D01085 | HMDB29723 | 5143 | 1.34 | 0.79 | 0.2378 | 0.1578 | 0.7700 | 0.6058 |
| 3034 | | | S-allylcysteine | LC/MS pos | 162.05833 | 43239 | | | 98280 | 0.73 | 0.31 | 0.0225 | 0.0289 | 1.3827 | 0.4245 |
| 3049 | | | stachydrine | LC/MS pos | 144.10191 | 34384 | C10172 | HMDB04827 | 115244 | 1.75 | 0.75 | 0.9436 | 0.3821 | 2.1440 | 1.6021 |
| 3054 | | | tartarate | LC/MS neg | 149.00916 | 15336 | C00898 | HMDB00956 | 444305 | 1.72 | 1.66 | 0.1455 | 0.1114 | 1.7360 | 2.8763 |
| 3055 | | | theanine | LC/MS pos | 175.10772 | 22206 | C01047 | HMDB34365 | 439378 | 0.99 | 1.07 | 0.6583 | 0.3102 | 0.2744 | 0.2928 |
| 3057 | | | thymol sulfate | LC/MS neg | 229.054 | 36095 | C09908 | HMDB01878 | | 1.29 | 0.48 | 0.1021 | 0.0865 | 3.8456 | 1.8532 |
| 3070 | | | 4-allylphenol sulfate | LC/MS neg | 213.0227 | 37181 | | | | 0.80 | 0.38 | 0.0263 | 0.0315 | 2.5218 | 0.9684 |
| 3071 | | | methyl glucopyranoside (alpha + beta) | LC/MS pos | 212.11268 | 46144 | | | | 1.31 | 0.80 | 0.4872 | 0.2552 | 1.9362 | 1.5396 |
| 3098 | | | 4-vinylguaiacol sulfate | LC/MS neg | 229.01761 | 48442 | | | | 0.81 | 0.57 | 0.0155 | 0.0234 | 1.4245 | 0.8159 |
| 3099 | | | pyrraline | LC/MS pos | 255.13394 | 48428 | | | | 1.28 | 1.14 | 0.4679 | 0.2482 | 0.9757 | 1.1162 |
| 3100 | | | umbelliferone sulfate | LC/MS neg | 240.98123 | 48674 | | | 129659 | 1.66 | 0.64 | 0.3018 | 0.1860 | 3.2169 | 2.0485 |
| 3102 | | | eugenol sulfate | LC/MS neg | 243.03326 | 48715 | | | 180632 | 1.19 | 0.15 | 0.1420 | 0.1094 | 12.2355 | 1.8394 |
| 3108 | | | isoeugenol sulfate | LC/MS neg | 243.03326 | 48997 | | | | 3.78 | 0.87 | 0.4282 | 0.2323 | 1.8474 | 1.6002 |
| 3126 | | Bacterial/Fungal | tartronate (hydroxymalonate) | LC/MS neg | 118.99859 | 20693 | C02287 | HMDB35227 | 45 | 0.50 | 0.48 | 0.0035 | 0.0079 | 1.4044 | 0.6673 |
| 3156 | | Drug | 2-hydroxyacetaminophen sulfate* | LC/MS neg | 246.00778 | 33173 | | | | 787.95 | 13.37 | 0.0097 | 0.0169 | 4.4804 | 59.9252 |
| 3157 | | | 2-methoxyacetaminophen sulfate* | LC/MS neg | 260.02343 | 47031 | | | | 432.16 | 18.75 | 0.0038 | 0.0086 | 0.0561 | 1.0516 |
| 3158 | | | 3-(cystein-S-yl)acetaminophen* | LC/MS pos | 271.07471 | 34365 | | | 5233914 | 25.58 | 9.80 | 0.0146 | 0.0228 | 0.0845 | 0.8285 |
| 3159 | | | 3-(N-acetyl-L-cystein-S-yl) acetaminophen | LC/MS neg | 311.07072 | 45721 | | | 83967 | 15.96 | 5.53 | 0.0170 | 0.0245 | 0.0965 | 0.5341 |
| 3160 | | | 4-acetaminophen sulfate | LC/MS neg | 230.01287 | 37475 | C06804 | HMDB59911 | 83939 | 1306.61 | 11.32 | 0.0095 | 0.0169 | 0.7167 | 8.1123 |
| 3161 | | | 4-acetamidophenol | LC/MS neg | 150.05605 | 12032 | C06804 | HMDB01859 | 1983 | 197.35 | 14.07 | 0.0102 | 0.0175 | 0.0965 | 1.3577 |
| 3162 | | | 4-acetamidophenylglucuronide | LC/MS neg | 326.08814 | 15736 | | HMDB10316 | 83944 | 361.77 | 17.34 | 0.0098 | 0.0170 | 0.0708 | 1.2280 |
| 3163 | | | 2-methoxyacetaminophen glucuronide* | LC/MS neg | 356.0987 | 33161 | | | | 50.09 | 20.62 | 0.0128 | 0.0208 | 0.0618 | 1.2742 |
| 3166 | | | ibuprofen | LC/MS pos | 224.16451 | 17799 | D00126 | HMDB01925 | 3672 | 6.10 | 4.11 | 0.6627 | 0.3112 | 0.1768 | 0.7265 |
| 3167 | | | 2-hydroxyibuprofen | LC/MS pos | 205.12231 | 43330 | | | | 1.73 | 1.60 | 0.5743 | 0.2849 | 0.4312 | 0.6901 |
| 3169 | | | carboxyibuprofen | LC/MS neg | 235.09758 | 43333 | | | 10444113 | 6.34 | 2.93 | 0.7914 | 0.3418 | 0.1163 | 0.3408 |
| 3181 | | | 4-acetylphenol sulfate | LC/MS neg | 215.00196 | 44620 | | | 4684006 | 0.94 | 0.78 | 0.0719 | 0.0680 | 1.1673 | 0.9059 |
| 3188 | | | 6-oxopiperidine-2-carboxylic acid | LC/MS pos | 142.05096 | 43231 | | | 3014237 | 0.92 | 0.86 | 0.1628 | 0.1207 | 1.1532 | 0.9940 |
| 3228 | | | 4-hydroxycoumarin | LC/MS neg | 161.02441 | 37445 | C20414 | | 54682930 | 1.42 | 0.67 | 0.7080 | 0.3228 | 1.1857 | 0.7970 |
| 3258 | | | hydrochlorothiazide | LC/MS neg | 295.95719 | 39625 | C07041 | HMDB01928 | 3639 | 0.99 | 0.99 | 0.3343 | 0.1953 | 0.9481 | 0.9401 |
| 3260 | | | hydroquinone sulfate | LC/MS neg | 188.98631 | 35322 | C00530 | HMDB02434 | 161220 | 1.96 | 1.01 | 0.8564 | 0.3601 | 1.2086 | 1.2180 |
| 3278 | | | lidocaine | LC/MS pos | 235.18049 | 35661 | D00358 | HMDB14426 | 3676 | 1.42 | 1.42 | 0.0970 | 0.0836 | 0.3582 | 0.5084 |
| 3284 | | | metformin | LC/MS pos | 130.10873 | 38306 | C07151 | HMDB01921 | 4091 | 0.93 | 0.14 | 0.3343 | 0.1953 | 0.1525 | 0.0221 |
| 3288 | | | metoprolol | LC/MS pos | 268.19072 | 18037 | D02358 | HMDB01932 | 4171 | 0.99 | 0.98 | 0.3343 | 0.1953 | 0.8911 | 0.8743 |
| 3289 | | | metoprolol acid metabolite* | LC/MS pos | 268.15434 | 34109 | | | 62936 | 0.98 | 0.98 | 0.3343 | 0.1953 | 0.8904 | 0.8736 |
| 3299 | | | N-ethylglycinexylidide | LC/MS pos | 207.14919 | 33080 | C16561 | HMDB60656 | 24415 | 1.17 | 1.17 | 0.1264 | 0.1008 | 0.6983 | 0.8140 |
| 3307 | | | omeprazole | LC/MS neg | 344.10743 | 38600 | C07324 | HMDB01913 | 4594 | 1.00 | 1.00 | | | 1.0000 | 1.0000 |
| 3335 | | | salicylate | LC/MS neg | 137.02442 | 1515 | C00805 | HMDB01895 | 338 | 0.88 | 0.57 | 0.0671 | 0.0654 | 1.6994 | 0.9720 |
| 3376 | | | 2-acetamidophenol sulfate | LC/MS neg | 230.01286 | 48580 | | | 181671 | 1.02 | 0.51 | 0.2743 | 0.1747 | 1.7075 | 0.8723 |
| 3421 | | Chemical | diglycerol | LC/MS pos | 167.0914 | 40702 | | | 42953 | 1.53 | 1.08 | 0.2157 | 0.1462 | 1.3049 | 1.4121 |
| 3422 | | | 1,2-propanediol | GC/MS | 117 | 38002 | C00583 | HMDB01881 | 1030 | 1.43 | 1.04 | 0.8440 | 0.3561 | 1.3576 | 1.4136 |
| 3429 | | | 2-pyrrolidinone | LC/MS pos | 86.06004 | 31675 | | HMDB02039 | 12025 | 1.01 | 0.90 | 0.3403 | 0.1971 | 1.0221 | 0.9225 |
| 3430 | | | sulfate* | LC/MS neg | 96.9601 | 46960 | C00059 | HMDB01448 | 1118 | 0.76 | 0.76 | 0.0168 | 0.0245 | 1.0917 | 0.8347 |
| 3433 | | | O-sulfo-L-tyrosine | LC/MS neg | 260.02343 | 45413 | | | 514186 | 1.40 | 1.40 | 0.0000 | 0.0000 | 0.8221 | 1.1480 |
| 3449 | | | 2-aminophenol sulfate | LC/MS neg | 188.0023 | 43266 | | HMDB61116 | 181670 | 0.85 | 0.67 | 0.0668 | 0.0654 | 2.2961 | 1.5358 |
| 3492 | | | dimethyl sulfone | LC/MS pos | 95.01613 | 43424 | C11142 | HMDB04983 | 6213 | 1.20 | 1.15 | 0.1404 | 0.1089 | 1.0538 | 1.2138 |
| 3494 | | | ectoine | LC/MS pos | 143.08151 | 35651 | C06231 | | 126041 | 1.58 | 1.26 | 0.5650 | 0.2831 | 0.9602 | 1.2085 |
| 3520 | | | phenylcarnitine* | LC/MS pos | 266.13869 | 43265 | | | | 1.08 | 0.86 | 0.5815 | 0.2862 | 1.3880 | 1.1878 |
| 3536 | | | N-methylpipecolate | LC/MS pos | 144.10191 | 47101 | | | 11862129;11286529 | 1.01 | 1.07 | 0.6826 | 0.3158 | 0.9978 | 1.0682 |
| 3538 | | | 4-methylbenzenesulfonate | LC/MS neg | 171.01213 | 43592 | C06677 | | 6101 | 0.82 | 0.76 | 0.0058 | 0.0118 | 1.3533 | 1.0330 |
| 3541 | | | 4-hydroxychlorothalonil | LC/MS neg | 244.90817 | 48441 | | | 34217 | 0.87 | 0.86 | 0.0006 | 0.0025 | 1.5026 | 1.2861 |
| 3545 | | | 1,2,3-benzenetriol sulfate (2) | LC/MS neg | 204.98123 | 48762 | | | | 1.64 | 1.44 | 0.7882 | 0.3418 | 1.9093 | 2.7573 |
| 3546 | | | 2-methyoxyresorcinol sulfate | LC/MS neg | 218.99688 | 48445 | | | | 1.39 | 1.08 | 0.3717 | 0.2099 | 1.4372 | 1.5579 |
| 3547 | | | 3-hydroxypyridine sulfate | LC/MS neg | 173.98665 | 48448 | | | | 0.72 | 0.64 | 0.0110 | 0.0182 | 1.2264 | 0.7808 |
| 3550 | | | cyclohexanebutanoic acid | LC/MS neg | 169.1234 | 48776 | | | 75200 | 1.35 | 1.09 | 0.5350 | 0.2733 | 0.9974 | 1.0850 |
| 3553 | | | ethyl paraben sulfate | LC/MS neg | 245.01253 | 48463 | | | | 1.11 | 0.69 | 0.1767 | 0.1280 | 9.3006 | 6.3786 |

*[Biochemical Name]*  Indicates compounds that have not been officially confirmed based on a standard, but we are confident in its identity.*

**Supplementary Table 2.** All significantly altered metabolites (p≤0.05), sorted according to their p-value.

**Comparison mean values significantly different:**

- (red) $p \le 0.05$, fold of change ≥ 1.00
- (green) $p \le 0.05$, fold of change < 1.00

| Pathway Sort Order | Super Pathway | Sub Pathway | Biochemical Name | Fold of Change, Paired t-Test: Post G-CSF / Pre G-CSF | | Paired t-Test: Post G-CSF / Pre G-CSF | | Mean Values | |
|---|---|---|---|---|---|---|---|---|---|
| | | | | Mean Pairs Ratio | Group Mean Ratio | *p*-value | *q*-value | Pre G-CSF | Post G-CSF |
| 10 | Amino Acid | Glycine, Serine and Threonine Metabolism | N-acetylserine | 1.63 | 1.61 | 0.0000 | 0.0000 | 0.8101 | 1.3054 |
| 1065 | Lipid | Polyunsaturated Fatty Acid (n3 and n6) | docosadienoate (22:2n6) | 2.96 | 2.79 | 0.0000 | 0.0000 | 0.6261 | 1.7463 |
| 2941 | Xenobiotics | Food Component/Plant | ergothioneine | 0.46 | 0.51 | 0.0000 | 0.0000 | 1.8383 | 0.9406 |
| 291 | Amino Acid | Methionine, Cysteine, SAM and Taurine Metabolism | N-acetylmethionine | 1.89 | 1.88 | 0.0000 | 0.0000 | 0.7976 | 1.4971 |
| 2508 | Nucleotide | Pyrimidine Metabolism, Orotate containing | orotidine | 1.89 | 1.90 | 0.0000 | 0.0000 | 0.8196 | 1.5540 |
| 227 | Amino Acid | Tryptophan Metabolism | tryptophan betaine | 0.55 | 0.60 | 0.0000 | 0.0000 | 3.6167 | 2.1754 |
| 309 | Amino Acid | Methionine, Cysteine, SAM and Taurine Metabolism | cystine | 0.67 | 0.65 | 0.0000 | 0.0000 | 1.2565 | 0.8189 |
| 2505 | Nucleotide | Pyrimidine Metabolism, Orotate containing | dihydroorotate | 0.73 | 0.72 | 0.0000 | 0.0000 | 1.2239 | 0.8836 |
| 84 | Amino Acid | Lysine Metabolism | 3-methylglutarylcarnitine (1) | 2.23 | 2.19 | 0.0000 | 0.0000 | 0.9545 | 2.0915 |
| 861 | Carbohydrate | Glycogen Metabolism | maltotriose | 39.18 | 16.27 | 0.0000 | 0.0000 | 0.0782 | 1.2721 |
| 864 | Carbohydrate | Glycogen Metabolism | maltose | 8.72 | 6.67 | 0.0000 | 0.0000 | 0.4175 | 2.7867 |
| 337 | Amino Acid | Urea cycle; Arginine and Proline Metabolism | N-acetylarginine | 0.65 | 0.66 | 0.0000 | 0.0000 | 1.4152 | 0.9292 |
| 2520 | Nucleotide | Pyrimidine Metabolism, Uracil containing | pseudouridine | 1.26 | 1.26 | 0.0000 | 0.0000 | 0.9344 | 1.1812 |
| 193 | Amino Acid | Tryptophan Metabolism | tryptophan | 0.75 | 0.75 | 0.0000 | 0.0000 | 1.1807 | 0.8829 |
| 3433 | Xenobiotics | Chemical | O-sulfo-L-tyrosine | 1.40 | 1.40 | 0.0000 | 0.0000 | 0.8221 | 1.1480 |
| 1676 | Lipid | Sterol | cholesterol | 0.82 | 0.82 | 0.0000 | 0.0001 | 1.1012 | 0.9015 |
| 318 | Amino Acid | Methionine, Cysteine, SAM and Taurine Metabolism | N-acetyltaurine | 1.62 | 1.60 | 0.0000 | 0.0001 | 0.9214 | 1.4727 |
| 1063 | Lipid | Polyunsaturated Fatty Acid (n3 and n6) | adrenate (22:4n6) | 2.36 | 2.25 | 0.0000 | 0.0001 | 0.7346 | 1.6524 |
| 278 | Amino Acid | Leucine, Isoleucine and Valine Metabolism | valine | 0.81 | 0.80 | 0.0000 | 0.0001 | 1.0966 | 0.8766 |
| 243 | Amino Acid | Leucine, Isoleucine and Valine Metabolism | isovalerate | 0.60 | 0.58 | 0.0000 | 0.0001 | 1.2311 | 0.7200 |
| 111 | Amino Acid | Phenylalanine and Tyrosine Metabolism | tyrosine | 0.78 | 0.77 | 0.0000 | 0.0001 | 1.1540 | 0.8850 |
| 211 | Amino Acid | Tryptophan Metabolism | picolinate | 0.62 | 0.60 | 0.0000 | 0.0001 | 1.3651 | 0.8190 |
| 2468 | Nucleotide | Purine Metabolism, Adenine containing | N6-carbamoylthreonyladenosine | 1.51 | 1.47 | 0.0000 | 0.0001 | 0.8161 | 1.2014 |
| 2440 | Nucleotide | Purine Metabolism, (Hypo)Xanthine/Inosine containing | allantoin | 2.22 | 2.13 | 0.0000 | 0.0001 | 0.7023 | 1.4963 |
| 26 | Amino Acid | Alanine and Aspartate Metabolism | N-acetylalanine | 1.29 | 1.28 | 0.0000 | 0.0001 | 0.8756 | 1.1198 |
| 2477 | Nucleotide | Purine Metabolism, Adenine containing | N6-succinyladenosine | 2.10 | 1.99 | 0.0000 | 0.0002 | 0.7039 | 1.4013 |
| 2430 | Nucleotide | Purine Metabolism, (Hypo)Xanthine/Inosine containing | inosine | 0.34 | 0.25 | 0.0000 | 0.0002 | 2.2074 | 0.5410 |
| 359 | Amino Acid | Creatine Metabolism | guanidinoacetate | 0.72 | 0.71 | 0.0000 | 0.0002 | 1.1968 | 0.8516 |
| 2485 | Nucleotide | Purine Metabolism, Guanine containing | guanosine | 0.31 | 0.22 | 0.0000 | 0.0002 | 2.2781 | 0.5097 |
| 1419 | Lipid | Phospholipid Metabolism | choline phosphate | 0.38 | 0.24 | 0.0000 | 0.0002 | 1.5486 | 0.3782 |
| 2459 | Nucleotide | Purine Metabolism, Adenine containing | N6-methyladenosine | 1.45 | 1.40 | 0.0000 | 0.0003 | 0.8416 | 1.1763 |
| 9 | Amino Acid | Glycine, Serine and Threonine Metabolism | serine | 0.79 | 0.79 | 0.0000 | 0.0003 | 1.1173 | 0.8779 |
| 336 | Amino Acid | Urea cycle; Arginine and Proline Metabolism | dimethylarginine (SDMA + ADMA) | 1.26 | 1.25 | 0.0001 | 0.0004 | 0.8930 | 1.1130 |
| 283 | Amino Acid | Leucine, Isoleucine and Valine Metabolism | isobutyrylglycine | 0.57 | 0.55 | 0.0001 | 0.0004 | 1.4584 | 0.8001 |
| 587 | Peptide | Dipeptide | leucylglycine | 0.51 | 0.51 | 0.0001 | 0.0004 | 1.1721 | 0.5961 |
| 1037 | Lipid | Long Chain Fatty Acid | eicosenoate (20:1) | 2.04 | 1.97 | 0.0001 | 0.0005 | 0.8493 | 1.6754 |
| 290 | Amino Acid | Methionine, Cysteine, SAM and Taurine Metabolism | methionine | 0.77 | 0.75 | 0.0001 | 0.0005 | 1.1628 | 0.8752 |
| 2494 | Nucleotide | Purine Metabolism, Guanine containing | N2,N2-dimethylguanosine | 1.28 | 1.25 | 0.0001 | 0.0006 | 0.9081 | 1.1346 |
| 273 | Amino Acid | Leucine, Isoleucine and Valine Metabolism | tigloylglycine | 0.61 | 0.57 | 0.0001 | 0.0006 | 1.2313 | 0.6963 |
| 327 | Amino Acid | Urea cycle; Arginine and Proline Metabolism | ornithine | 0.68 | 0.67 | 0.0001 | 0.0007 | 1.2163 | 0.8201 |
| 2608 | ofactors and Vitamii | Ascorbate and Aldarate Metabolism | gulonic acid* | 2.06 | 1.75 | 0.0001 | 0.0008 | 0.6970 | 1.2204 |
| 2644 | ofactors and Vitamii | Hemoglobin and Porphyrin Metabolism | bilirubin (Z,Z) | 0.56 | 0.51 | 0.0001 | 0.0008 | 1.7259 | 0.8726 |
| 73 | Amino Acid | Lysine Metabolism | lysine | 0.83 | 0.82 | 0.0002 | 0.0009 | 1.1419 | 0.9333 |
| 112 | Amino Acid | Phenylalanine and Tyrosine Metabolism | N-acetyltyrosine | 0.72 | 0.73 | 0.0002 | 0.0013 | 1.1960 | 0.8752 |
| 237 | Amino Acid | Leucine, Isoleucine and Valine Metabolism | leucine | 0.81 | 0.80 | 0.0002 | 0.0012 | 1.1029 | 0.8828 |
| 813 | Carbohydrate | Glycolysis, Gluconeogenesis, and Pyruvate Metabolism | lactate | 1.46 | 1.41 | 0.0002 | 0.0009 | 0.8890 | 1.2513 |
| 1066 | Lipid | Polyunsaturated Fatty Acid (n3 and n6) | dihomo-linoleate (20:2n6) | 1.90 | 1.77 | 0.0002 | 0.0010 | 0.8468 | 1.4958 |
| 2548 | Nucleotide | Pyrimidine Metabolism, Cytidine containing | N4-acetylcytidine | 1.46 | 1.45 | 0.0002 | 0.0013 | 0.9307 | 1.3468 |
| 973 | Carbohydrate | Aminosugar Metabolism | erythronate* | 1.26 | 1.26 | 0.0003 | 0.0016 | 0.9399 | 1.1804 |
| 1152 | Lipid | Fatty Acid, Dicarboxylate | octadecanedioate | 2.08 | 1.94 | 0.0003 | 0.0013 | 0.9011 | 1.7485 |
| 1461 | Lipid | Phospholipid Metabolism | 1-(1-enyl-palmitoyl)-GPC (P-16:0)* | 0.79 | 0.77 | 0.0003 | 0.0016 | 1.1293 | 0.8739 |
| 203 | Amino Acid | Tryptophan Metabolism | kynurenine | 0.84 | 0.83 | 0.0004 | 0.0020 | 1.1147 | 0.9269 |
| 245 | Amino Acid | Leucine, Isoleucine and Valine Metabolism | isovalerylcarnitine | 0.74 | 0.74 | 0.0005 | 0.0022 | 1.1596 | 0.8548 |
| 266 | Amino Acid | Leucine, Isoleucine and Valine Metabolism | isoleucine | 0.80 | 0.78 | 0.0005 | 0.0024 | 1.1221 | 0.8719 |
| 307 | Amino Acid | Methionine, Cysteine, SAM and Taurine Metabolism | cysteine | 0.69 | 0.66 | 0.0005 | 0.0022 | 1.2676 | 0.8375 |
| 352 | Amino Acid | Urea cycle; Arginine and Proline Metabolism | N-acetylcitrulline | 0.59 | 0.60 | 0.0005 | 0.0022 | 1.9575 | 1.1697 |
| 1538 | Lipid | Lysolipid | 1-oleoyl-GPI (18:1)* | 0.59 | 0.55 | 0.0005 | 0.0022 | 1.3719 | 0.7493 |
| 2609 | ofactors and Vitamii | Tocopherol Metabolism | alpha-tocopherol | 0.82 | 0.81 | 0.0005 | 0.0023 | 1.0892 | 0.8851 |
| 1231 | Lipid | Fatty Acid Metabolism(Acyl Carnitine) | hydroxybutyrylcarnitine* | 1.49 | 1.44 | 0.0006 | 0.0025 | 0.8635 | 1.2458 |
| 1446 | Lipid | Phospholipid Metabolism | 1-oleoyl-2-linoleoyl-GPI (18:1/18:2)* | 0.74 | 0.70 | 0.0006 | 0.0025 | 1.2817 | 0.8955 |
| 2582 | ofactors and Vitamii | Nicotinate and Nicotinamide Metabolism | 1-methylnicotinamide | 0.66 | 0.56 | 0.0006 | 0.0025 | 1.2096 | 0.6797 |
| 3541 | Xenobiotics | Chemical | 4-hydroxychlorothalonil | 0.87 | 0.86 | 0.0006 | 0.0025 | 1.5026 | 1.2861 |
| 204 | Amino Acid | Tryptophan Metabolism | kynurenate | 0.77 | 0.77 | 0.0007 | 0.0027 | 1.2853 | 0.9840 |
| 244 | Amino Acid | Leucine, Isoleucine and Valine Metabolism | isovalerylglycine | 0.54 | 0.55 | 0.0008 | 0.0030 | 1.2632 | 0.6927 |
| 816 | Carbohydrate | Glycolysis, Gluconeogenesis, and Pyruvate Metabolism | glycerate | 0.83 | 0.83 | 0.0008 | 0.0032 | 1.0458 | 0.8642 |
| 1031 | Lipid | Long Chain Fatty Acid | nonadecanoate (19:0) | 1.34 | 1.35 | 0.0008 | 0.0032 | 0.9524 | 1.2840 |
| 1151 | Lipid | Fatty Acid, Dicarboxylate | hexadecanedioate | 1.71 | 1.68 | 0.0009 | 0.0034 | 0.9444 | 1.5827 |
| 2732 | Xenobiotics | Benzoate Metabolism | propyl 4-hydroxybenzoate sulfate | 2.04 | 1.83 | 0.0009 | 0.0034 | 0.6908 | 1.2675 |
| 235 | Amino Acid | Tryptophan Metabolism | N-acetylkynurenine (2) | 0.68 | 0.60 | 0.0010 | 0.0035 | 1.1806 | 0.7137 |
| 1213 | Lipid | Fatty Acid Metabolism (also BCAA Metabolism) | butyrylcarnitine | 1.29 | 1.25 | 0.0010 | 0.0035 | 1.0910 | 1.3637 |
| 1233 | Lipid | Fatty Acid Metabolism(Acyl Carnitine) | hexanoylcarnitine | 1.39 | 1.37 | 0.0010 | 0.0035 | 1.0121 | 1.3885 |
| 1447 | Lipid | Phospholipid Metabolism | 1-palmitoyl-2-arachidonoyl-GPI (16:0/20:4)* | 0.70 | 0.66 | 0.0010 | 0.0035 | 1.2151 | 0.7965 |
| 2589 | ofactors and Vitamii | Nicotinate and Nicotinamide Metabolism | N1-Methyl-2-pyridone-5-carboxamide | 0.67 | 0.62 | 0.0010 | 0.0035 | 1.2544 | 0.7773 |
| 1366 | Lipid | Eicosanoid | 12-HETE | 0.60 | 0.31 | 0.0011 | 0.0036 | 2.9009 | 0.9121 |
| 248 | Amino Acid | Leucine, Isoleucine and Valine Metabolism | beta-hydroxyisovalerate | 0.83 | 0.82 | 0.0012 | 0.0041 | 1.1749 | 0.9671 |
| 366 | Amino Acid | Polyamine Metabolism | 5-methylthioadenosine (MTA) | 1.19 | 1.18 | 0.0012 | 0.0041 | 0.9092 | 1.0733 |
| 2605 | ofactors and Vitamii | Ascorbate and Aldarate Metabolism | threonate | 0.78 | 0.76 | 0.0012 | 0.0041 | 1.0236 | 0.7782 |
| 2761 | Xenobiotics | Food Component/Plant | 2-piperidinone | 0.73 | 0.67 | 0.0012 | 0.0041 | 1.4596 | 0.9842 |
| 53 | Amino Acid | Histidine Metabolism | histidine | 0.84 | 0.82 | 0.0013 | 0.0042 | 1.0801 | 0.8873 |
| 194 | Amino Acid | Tryptophan Metabolism | N-acetyltryptophan | 0.75 | 0.73 | 0.0016 | 0.0050 | 1.2745 | 0.9310 |
| 2493 | Nucleotide | Purine Metabolism, Guanine containing | N2-methylguanosine | 1.35 | 1.32 | 0.0016 | 0.0051 | 0.8647 | 1.1409 |
| 405 | Peptide | Gamma-glutamyl Amino Acid | gamma-glutamyltryptophan | 0.80 | 0.78 | 0.0017 | 0.0053 | 1.1941 | 0.9356 |
| 1421 | Lipid | Phospholipid Metabolism | glycerophosphorylcholine (GPC) | 0.77 | 0.76 | 0.0017 | 0.0051 | 1.1454 | 0.8651 |
| 98 | Amino Acid | Phenylalanine and Tyrosine Metabolism | phenylalanine | 0.88 | 0.87 | 0.0019 | 0.0057 | 1.0679 | 0.9320 |
| 812 | Carbohydrate | Glycolysis, Gluconeogenesis, and Pyruvate Metabolism | pyruvate | 1.60 | 1.42 | 0.0019 | 0.0057 | 0.9167 | 1.3015 |
| 1122 | Lipid | Fatty Acid, Branched | 17-methylstearate | 1.71 | 1.63 | 0.0019 | 0.0057 | 0.7957 | 1.2957 |
| 1216 | Lipid | Fatty Acid Metabolism (also BCAA Metabolism) | propionylcarnitine | 0.84 | 0.84 | 0.0021 | 0.0061 | 1.0682 | 0.8925 |
| 2670 | ofactors and Vitamii | Vitamin B6 Metabolism | pyridoxate | 0.77 | 0.70 | 0.0021 | 0.0061 | 1.4754 | 1.0353 |
| 71 | Amino Acid | Histidine Metabolism | 4-imidazoleacetate | 0.67 | 0.67 | 0.0022 | 0.0062 | 1.0848 | 0.7269 |
| 1017 | Lipid | Long Chain Fatty Acid | pentadecanoate (15:0) | 1.40 | 1.36 | 0.0022 | 0.0062 | 1.0312 | 1.3977 |
| 82 | Amino Acid | Lysine Metabolism | glutarate (pentanedioate) | 0.66 | 0.62 | 0.0023 | 0.0064 | 1.7006 | 1.0553 |
| 1595 | Lipid | Glycerolipid Metabolism | glycerol | 0.67 | 0.71 | 0.0024 | 0.0067 | 1.3120 | 0.9366 |
| 1657 | Lipid | Sphingolipid Metabolism | sphingomyelin (d18:2/16:0, d18:1/16:1)* | 0.86 | 0.86 | 0.0025 | 0.0068 | 1.0752 | 0.9201 |
| 2533 | Nucleotide | Pyrimidine Metabolism, Uracil containing | 4-ureidobutyrate | 0.72 | 0.71 | 0.0025 | 0.0068 | 1.2137 | 0.8620 |
| 1788 | Lipid | Steroid | 5alpha-androstan-3alpha,17beta-diol monosulfate (1) | 1.32 | 1.26 | 0.0026 | 0.0069 | 1.1409 | 1.4394 |
| 141 | Amino Acid | Phenylalanine and Tyrosine Metabolism | 3-methoxytyrosine | 1.11 | 1.11 | 0.0027 | 0.0072 | 1.0033 | 1.1138 |
| 1655 | Lipid | Sphingolipid Metabolism | sphingomyelin (d18:2/14:0, d18:1/14:1)* | 0.78 | 0.77 | 0.0027 | 0.0072 | 1.1159 | 0.8560 |
| 793 | Carbohydrate | Glycolysis, Gluconeogenesis, and Pyruvate Metabolism | 1,5-anhydroglucitol (1,5-AG) | 0.91 | 0.92 | 0.0028 | 0.0073 | 0.9963 | 0.9126 |
| 402 | Peptide | Gamma-glutamyl Amino Acid | gamma-glutamylmethionine | 0.78 | 0.74 | 0.0029 | 0.0073 | 1.2240 | 0.9080 |
| 1399 | Lipid | Inositol Metabolism | inositol 1-phosphate (I1P) | 0.78 | 0.75 | 0.0029 | 0.0073 | 1.1882 | 0.8856 |
| 1445 | Lipid | Phospholipid Metabolism | 1-palmitoyl-2-linoleoyl-GPI (16:0/18:2)* | 0.78 | 0.76 | 0.0029 | 0.0073 | 1.1804 | 0.8935 |
| 75 | Amino Acid | Lysine Metabolism | N6-acetyllysine | 1.15 | 1.14 | 0.0030 | 0.0074 | 0.9215 | 1.0495 |
| 312 | Amino Acid | Methionine, Cysteine, SAM and Taurine Metabolism | cysteine s-sulfate | 0.69 | 0.54 | 0.0030 | 0.0074 | 1.2694 | 0.6871 |
| 1510 | Lipid | Lysolipid | 1-palmitoyl-GPE (16:0) | 0.77 | 0.73 | 0.0030 | 0.0073 | 1.2350 | 0.9027 |
| 325 | Amino Acid | Urea cycle; Arginine and Proline Metabolism | arginine | 0.85 | 0.84 | 0.0031 | 0.0075 | 1.1366 | 0.9536 |
| 1448 | Lipid | Phospholipid Metabolism | 1-stearoyl-2-linoleoyl-GPI (18:0/18:2)* | 0.79 | 0.78 | 0.0031 | 0.0075 | 1.1257 | 0.8800 |
| 218 | Amino Acid | Tryptophan Metabolism | 5-hydroxyindoleacetate | 0.63 | 0.49 | 0.0033 | 0.0078 | 1.5830 | 0.7758 |
| 2534 | Nucleotide | Pyrimidine Metabolism, Uracil containing | 3-ureidopropionate | 1.75 | 1.64 | 0.0033 | 0.0077 | 0.8271 | 1.3524 |
| 545 | Peptide | Dipeptide | glycylvaline | 0.56 | 0.15 | 0.0034 | 0.0079 | 6.9270 | 1.0565 |
| 1249 | Lipid | Carnitine Metabolism | carnitine | 1.07 | 1.07 | 0.0034 | 0.0079 | 0.9669 | 1.0327 |
| 3126 | Xenobiotics | Bacterial/Fungal | tartronate (hydroxymalonate) | 0.50 | 0.48 | 0.0035 | 0.0079 | 1.4044 | 0.6673 |
| 1436 | Lipid | Phospholipid Metabolism | 1-palmitoyl-2-arachidonoyl-GPC (16:0/20:4)* | 0.88 | 0.87 | 0.0036 | 0.0081 | 1.0829 | 0.9405 |
| 3157 | Xenobiotics | Drug | 2-methoxyacetaminophen sulfate* | 432.16 | 18.75 | 0.0038 | 0.0086 | 0.0561 | 1.0516 |
| 738 | Peptide | Dipeptide | valylleucine | 0.80 | 0.78 | 0.0039 | 0.0087 | 1.1095 | 0.8613 |
| 326 | Amino Acid | Urea cycle; Arginine and Proline Metabolism | urea | 0.84 | 0.82 | 0.0040 | 0.0087 | 1.1318 | 0.9297 |
| 1267 | Lipid | Fatty Acid, Monohydroxy | 3-hydroxysebacate | 2.15 | 1.72 | 0.0041 | 0.0089 | 0.9934 | 1.7070 |
| 2745 | Xenobiotics | Xanthine Metabolism | 1-methylxanthine | 0.68 | 0.55 | 0.0041 | 0.0089 | 1.7840 | 0.9756 |
| 2645 | ofactors and Vitamii | Hemoglobin and Porphyrin Metabolism | bilirubin (E,E)* | 0.74 | 0.69 | 0.0042 | 0.0091 | 1.3280 | 0.9198 |
| 1032 | Lipid | Long Chain Fatty Acid | 10-nonadecenoate (19:1n9) | 1.88 | 1.74 | 0.0044 | 0.0094 | 0.9312 | 1.6195 |
| 277 | Amino Acid | Leucine, Isoleucine and Valine Metabolism | ethylmalonate | 1.26 | 1.24 | 0.0047 | 0.0099 | 1.1462 | 1.4161 |
| 1444 | Lipid | Phospholipid Metabolism | 1-stearoyl-2-arachidonoyl-GPI (18:0/20:4)* | 0.86 | 0.82 | 0.0051 | 0.0108 | 1.2290 | 1.0081 |
| 735 | Peptide | Dipeptide | valylglycine | 0.71 | 0.57 | 0.0054 | 0.0112 | 1.2603 | 0.7244 |
| 282 | Amino Acid | Leucine, Isoleucine and Valine Metabolism | isobutyrylcarnitine | 0.82 | 0.78 | 0.0056 | 0.0116 | 1.2666 | 0.9868 |
| 1533 | Lipid | Lysolipid | 1-palmitoyl-GPI (16:0)* | 0.68 | 0.61 | 0.0058 | 0.0119 | 1.3543 | 0.8292 |
| 3538 | Xenobiotics | Chemical | 4-methylbenzenesulfonate | 0.82 | 0.76 | 0.0058 | 0.0118 | 1.3533 | 1.0330 |
| 362 | Amino Acid | Polyamine Metabolism | acisoga | 1.33 | 1.25 | 0.0060 | 0.0121 | 0.8772 | 1.1006 |
| 148 | Amino Acid | Phenylalanine and Tyrosine Metabolism | homovanillate (HVA) | 0.76 | 0.77 | 0.0062 | 0.0125 | 1.1547 | 0.8859 |

p ≤ 0.05, fold of change ≥ 1.00 (red)

p ≤ 0.05, fold of change < 1.00 (green)

| Pathway Sort Order | Super Pathway | Sub Pathway | Biochemical Name | Fold of Change, Paired t-Test: Post G-CSF / Pre G-CSF | | Paired t-Test: Post G-CSF / Pre G-CSF | | Mean Values | |
|---|---|---|---|---|---|---|---|---|---|
| | | | | Mean Pairs Ratio | Group Mean Ratio | *p*-value | *q*-value | Pre G-CSF | Post G-CSF |
| 1021 | Lipid | Long Chain Fatty Acid | margarate (17:0) | 1.55 | 1.50 | 0.0064 | 0.0127 | 1.0301 | 1.5499 |
| 1418 | Lipid | Phospholipid Metabolism | choline | 0.89 | 0.89 | 0.0065 | 0.0127 | 1.0740 | 0.9552 |
| 28 | Amino Acid | Alanine and Aspartate Metabolism | aspartate | 0.64 | 0.60 | 0.0068 | 0.0132 | 1.3009 | 0.7755 |
| 397 | Peptide | Gamma-glutamyl Amino Acid | gamma-glutamylglycine | 0.67 | 0.50 | 0.0068 | 0.0132 | 1.7954 | 0.9043 |
| 1139 | Lipid | Fatty Acid, Dicarboxylate | maleate | 0.77 | 0.73 | 0.0070 | 0.0134 | 1.3816 | 1.0110 |
| 1555 | Lipid | Lysolipid | 1-arachidonoyl-GPA (20:4) | 0.68 | 0.59 | 0.0070 | 0.0134 | 1.3556 | 0.7951 |
| 256 | Amino Acid | Leucine, Isoleucine and Valine Metabolism | alpha-hydroxyisovalerate | 1.21 | 1.27 | 0.0071 | 0.0134 | 1.0033 | 1.2732 |
| 2528 | Nucleotide | Pyrimidine Metabolism, Uracil containing | 5,6-dihydrouracil | 1.18 | 1.19 | 0.0071 | 0.0134 | 0.9634 | 1.1445 |
| 317 | Amino Acid | Methionine, Cysteine, SAM and Taurine Metabolism | taurine | 0.78 | 0.73 | 0.0077 | 0.0144 | 1.2349 | 0.9064 |
| 1643 | Lipid | Sphingolipid Metabolism | sphingosine 1-phosphate | 0.84 | 0.82 | 0.0078 | 0.0144 | 1.0986 | 0.9009 |
| 267 | Amino Acid | Leucine, Isoleucine and Valine Metabolism | allo-isoleucine | 1.31 | 1.25 | 0.0085 | 0.0155 | 0.9935 | 1.2390 |
| 2743 | Xenobiotics | Xanthine Metabolism | 3,7-dimethylurate | 0.73 | 0.63 | 0.0085 | 0.0155 | 1.7071 | 1.0823 |
| 1025 | Lipid | Long Chain Fatty Acid | oleate (18:1n9) | 1.70 | 1.61 | 0.0086 | 0.0156 | 1.0068 | 1.6250 |
| 1062 | Lipid | Polyunsaturated Fatty Acid (n3 and n6) | arachidonate (20:4n6) | 0.76 | 0.73 | 0.0093 | 0.0168 | 1.1828 | 0.8666 |
| 3160 | Xenobiotics | Drug | 4-acetaminophen sulfate | 1306.61 | 11.32 | 0.0095 | 0.0169 | 0.7167 | 8.1123 |
| 1394 | Lipid | Inositol Metabolism | scyllo-inositol | 0.79 | 0.71 | 0.0096 | 0.0169 | 1.5570 | 1.0997 |
| 25 | Amino Acid | Alanine and Aspartate Metabolism | alanine | 1.13 | 1.11 | 0.0097 | 0.0169 | 0.9654 | 1.0764 |
| 3156 | Xenobiotics | Drug | 2-hydroxyacetaminophen sulfate* | 787.95 | 13.37 | 0.0097 | 0.0169 | 4.4804 | 59.9252 |
| 3162 | Xenobiotics | Drug | 4-acetamidophenylglucuronide | 361.77 | 17.34 | 0.0098 | 0.0170 | 0.0708 | 1.2280 |
| 1059 | Lipid | Polyunsaturated Fatty Acid (n3 and n6) | linolenate [alpha or gamma; (18:3n3 or 6)] | 1.80 | 1.70 | 0.0101 | 0.0173 | 0.9509 | 1.6206 |
| 3161 | Xenobiotics | Drug | 4-acetamidophenol | 197.35 | 14.07 | 0.0102 | 0.0175 | 0.0965 | 1.3577 |
| 269 | Amino Acid | Leucine, Isoleucine and Valine Metabolism | 3-methyl-2-oxovalerate | 0.86 | 0.86 | 0.0105 | 0.0176 | 1.1373 | 0.9769 |
| 1180 | Lipid | Fatty Acid, Amide | oleamide | 0.69 | 0.37 | 0.0105 | 0.0176 | 2.6343 | 0.9708 |
| 2532 | Nucleotide | Pyrimidine Metabolism, Uracil containing | 2'-deoxyuridine | 1.54 | 1.44 | 0.0105 | 0.0176 | 0.8843 | 1.2728 |
| 1027 | Lipid | Long Chain Fatty Acid | cis-vaccenate (18:1n7) | 1.58 | 1.38 | 0.0110 | 0.0182 | 0.9850 | 1.3596 |
| 3547 | Xenobiotics | Chemical | 3-hydroxypyridine sulfate | 0.72 | 0.64 | 0.0110 | 0.0182 | 1.2264 | 0.7808 |
| 2717 | Xenobiotics | Benzoate Metabolism | 4-vinylphenol sulfate | 0.73 | 0.71 | 0.0116 | 0.0191 | 3.2346 | 2.3007 |
| 2492 | Nucleotide | Purine Metabolism, Guanine containing | N1-methylguanosine | 1.16 | 1.15 | 0.0122 | 0.0200 | 0.9447 | 1.0890 |
| 3163 | Xenobiotics | Drug | 2-methoxyacetaminophen glucuronide* | 50.09 | 20.62 | 0.0128 | 0.0208 | 0.0618 | 1.2742 |
| 400 | Peptide | Gamma-glutamyl Amino Acid | gamma-glutamylleucine | 0.86 | 0.83 | 0.0132 | 0.0213 | 1.1059 | 0.9147 |
| 74 | Amino Acid | Lysine Metabolism | N2-acetyllysine | 0.80 | 0.72 | 0.0133 | 0.0213 | 1.2041 | 0.8660 |
| 1762 | Lipid | Steroid | cortisone | 0.85 | 0.82 | 0.0138 | 0.0220 | 1.1471 | 0.9461 |
| 2519 | Nucleotide | Pyrimidine Metabolism, Uracil containing | uracil | 1.34 | 1.30 | 0.0139 | 0.0220 | 0.9029 | 1.1707 |
| 1012 | Lipid | Medium Chain Fatty Acid | laurate (12:0) | 1.49 | 1.39 | 0.0146 | 0.0228 | 0.9576 | 1.3346 |
| 3158 | Xenobiotics | Drug | 3-(cystein-S-yl)acetaminophen* | 25.58 | 9.80 | 0.0146 | 0.0228 | 0.0845 | 0.8285 |
| 407 | Peptide | Gamma-glutamyl Amino Acid | gamma-glutamylvaline | 0.88 | 0.86 | 0.0147 | 0.0228 | 1.0957 | 0.9398 |
| 238 | Amino Acid | Leucine, Isoleucine and Valine Metabolism | N-acetylleucine | 0.83 | 0.83 | 0.0153 | 0.0234 | 1.0607 | 0.8766 |
| 1654 | Lipid | Sphingolipid Metabolism | sphingomyelin (d18:1/14:0, d16:1/16:0)* | 0.87 | 0.88 | 0.0155 | 0.0234 | 1.0413 | 0.9144 |
| 3098 | Xenobiotics | Food Component/Plant | 4-vinylguaiacol sulfate | 0.81 | 0.57 | 0.0155 | 0.0234 | 1.4245 | 0.8159 |
| 79 | Amino Acid | Lysine Metabolism | 2-aminoadipate | 0.83 | 0.75 | 0.0157 | 0.0234 | 1.3280 | 0.9993 |
| 240 | Amino Acid | Leucine, Isoleucine and Valine Metabolism | 4-methyl-2-oxopentanoate | 0.86 | 0.86 | 0.0157 | 0.0234 | 1.1075 | 0.9495 |
| 569 | Peptide | Dipeptide | isoleucylglycine | 0.82 | 0.75 | 0.0157 | 0.0234 | 1.1828 | 0.8889 |
| 1006 | Lipid | Medium Chain Fatty Acid | heptanoate (7:0) | 0.86 | 0.85 | 0.0157 | 0.0234 | 1.1133 | 0.9497 |
| 2705 | Xenobiotics | Benzoate Metabolism | 3-methyl catechol sulfate (2) | 0.74 | 0.52 | 0.0158 | 0.0234 | 1.3245 | 0.6851 |
| 406 | Peptide | Gamma-glutamyl Amino Acid | gamma-glutamyltyrosine | 0.85 | 0.82 | 0.0166 | 0.0244 | 1.0817 | 0.8885 |
| 2607 | ofactors and Vitami | Ascorbate and Aldarate Metabolism | oxalate (ethanedioate) | 0.65 | 0.64 | 0.0166 | 0.0244 | 1.1510 | 0.7406 |
| 3430 | Xenobiotics | Chemical | sulfate* | 0.76 | 0.76 | 0.0168 | 0.0245 | 1.0917 | 0.8347 |
| 306 | Amino Acid | Methionine, Cysteine, SAM and Taurine Metabolism | 2-hydroxybutyrate (AHB) | 1.41 | 1.30 | 0.0170 | 0.0245 | 0.9659 | 1.2542 |
| 3159 | Xenobiotics | Drug | 3-(N-acetyl-L-cystein-S-yl) acetaminophen | 15.96 | 5.53 | 0.0170 | 0.0245 | 0.0965 | 0.5341 |
| 279 | Amino Acid | Leucine, Isoleucine and Valine Metabolism | N-acetylvaline | 1.09 | 1.08 | 0.0175 | 0.0248 | 0.9730 | 1.0539 |
| 1003 | Energy | Oxidative Phosphorylation | phosphate | 0.81 | 0.81 | 0.0175 | 0.0248 | 1.0886 | 0.8828 |
| 1266 | Lipid | Fatty Acid, Monohydroxy | 3-hydroxydecanoate | 1.37 | 1.25 | 0.0175 | 0.0248 | 1.0661 | 1.3361 |
| 188 | Amino Acid | Phenylalanine and Tyrosine Metabolism | N-formylphenylalanine | 0.78 | 0.61 | 0.0178 | 0.0251 | 1.2909 | 0.7914 |
| 106 | Amino Acid | Phenylalanine and Tyrosine Metabolism | phenylacetate | 0.78 | 0.71 | 0.0179 | 0.0251 | 1.3119 | 0.9367 |
| 15 | Amino Acid | Glycine, Serine and Threonine Metabolism | N-acetylthreonine | 1.26 | 1.23 | 0.0184 | 0.0256 | 0.9305 | 1.1405 |
| 1361 | Lipid | Eicosanoid | 5-HETE | 1.99 | 1.77 | 0.0187 | 0.0259 | 0.6777 | 1.1989 |
| 1018 | Lipid | Long Chain Fatty Acid | palmitate (16:0) | 1.24 | 1.24 | 0.0193 | 0.0267 | 1.0105 | 1.2489 |
| 906 | Carbohydrate | Fructose, Mannose and Galactose Metabolism | mannose | 1.40 | 1.23 | 0.0197 | 0.0270 | 0.9723 | 1.1979 |
| 1022 | Lipid | Long Chain Fatty Acid | 10-heptadecenoate (17:1n7) | 1.77 | 1.65 | 0.0198 | 0.0270 | 1.0032 | 1.6548 |
| 1023 | Lipid | Long Chain Fatty Acid | stearate (18:0) | 1.23 | 1.23 | 0.0202 | 0.0273 | 0.9912 | 1.2197 |
| 1784 | Lipid | Steroid | 4-androsten-3beta,17beta-diol disulfate (2) | 0.79 | 0.82 | 0.0203 | 0.0274 | 1.2465 | 1.0254 |
| 1664 | Lipid | Mevalonate Metabolism | 3-hydroxy-3-methylglutarate | 0.85 | 0.80 | 0.0205 | 0.0275 | 1.1788 | 0.9375 |
| 54 | Amino Acid | Histidine Metabolism | N-acetylhistidine | 0.78 | 0.81 | 0.0212 | 0.0283 | 1.0851 | 0.8799 |
| 1133 | Lipid | Fatty Acid, Dicarboxylate | 2-hydroxyglutarate | 1.71 | 1.61 | 0.0214 | 0.0284 | 0.9920 | 1.5942 |
| 2452 | Nucleotide | Purine Metabolism, Adenine containing | adenosine | 0.78 | 0.29 | 0.0215 | 0.0284 | 3.4216 | 0.9964 |
| 1834 | Lipid | Steroid | pregnanolone/allopregnanolone sulfate | 0.79 | 0.56 | 0.0216 | 0.0284 | 1.0092 | 0.5669 |
| 2448 | Nucleotide | Purine Metabolism, Adenine containing | adenosine 3',5'-cyclic monophosphate (cAMP) | 0.81 | 0.77 | 0.0217 | 0.0284 | 1.1050 | 0.8499 |
| 270 | Amino Acid | Leucine, Isoleucine and Valine Metabolism | 2-methylbutyrylcarnitine (C5) | 0.89 | 0.86 | 0.0219 | 0.0285 | 1.0657 | 0.9210 |
| 1057 | Lipid | Polyunsaturated Fatty Acid (n3 and n6) | linoleate (18:2n6) | 1.42 | 1.38 | 0.0222 | 0.0288 | 1.0030 | 1.3855 |
| 3034 | Xenobiotics | Food Component/Plant | S-allylcysteine | 0.73 | 0.31 | 0.0225 | 0.0289 | 1.3827 | 0.4245 |
| 180 | Amino Acid | Phenylalanine and Tyrosine Metabolism | 5-hydroxymethyl-2-furoic acid | 0.82 | 0.54 | 0.0227 | 0.0289 | 1.6842 | 0.9148 |
| 1519 | Lipid | Lysolipid | 1-linoleoyl-GPE (18:2)* | 0.84 | 0.76 | 0.0228 | 0.0289 | 1.1630 | 0.8872 |
| 2521 | Nucleotide | Pyrimidine Metabolism, Uracil containing | 5-methyluridine (ribothymidine) | 0.84 | 0.83 | 0.0228 | 0.0289 | 1.0719 | 0.8916 |
| 2704 | Xenobiotics | Benzoate Metabolism | 3-methyl catechol sulfate (1) | 0.77 | 0.53 | 0.0229 | 0.0289 | 1.4830 | 0.7879 |
| 1016 | Lipid | Long Chain Fatty Acid | myristoleate (14:1n5) | 1.80 | 1.64 | 0.0233 | 0.0293 | 1.3317 | 2.1785 |
| 1268 | Lipid | Fatty Acid, Monohydroxy | 3-hydroxylaurate | 1.57 | 1.36 | 0.0234 | 0.0293 | 0.9687 | 1.3179 |
| 1222 | Lipid | Fatty Acid Metabolism(Acyl Glycine) | hexanoylglycine | 1.38 | 1.45 | 0.0237 | 0.0294 | 0.9499 | 1.3790 |
| 1699 | Lipid | Sterol | campesterol | 0.87 | 0.81 | 0.0241 | 0.0298 | 1.1340 | 0.9240 |
| 284 | Amino Acid | Leucine, Isoleucine and Valine Metabolism | 3-hydroxyisobutyrate | 0.86 | 0.81 | 0.0243 | 0.0299 | 1.1686 | 0.9447 |
| 1280 | Lipid | Fatty Acid, Monohydroxy | 16-hydroxypalmitate | 1.44 | 1.39 | 0.0246 | 0.0300 | 0.9760 | 1.3539 |
| 1015 | Lipid | Long Chain Fatty Acid | myristate (14:0) | 1.64 | 1.51 | 0.0247 | 0.0300 | 1.0213 | 1.5461 |
| 1247 | Lipid | Fatty Acid Metabolism(Acyl Carnitine) | myristoleoylcarnitine* | 3.62 | 2.54 | 0.0247 | 0.0300 | 0.6640 | 1.6882 |
| 198 | Amino Acid | Tryptophan Metabolism | indoleacetate | 0.86 | 0.82 | 0.0250 | 0.0301 | 1.4667 | 1.1978 |
| 30 | Amino Acid | Alanine and Aspartate Metabolism | asparagine | 0.94 | 0.93 | 0.0258 | 0.0310 | 1.0885 | 1.0128 |
| 3070 | Xenobiotics | Food Component/Plant | 4-allylphenol sulfate | 0.80 | 0.38 | 0.0263 | 0.0315 | 2.5218 | 0.9684 |
| 982 | Energy | TCA Cycle | aconitate [cis or trans] | 1.29 | 1.22 | 0.0264 | 0.0315 | 0.9360 | 1.1391 |
| 1009 | Lipid | Medium Chain Fatty Acid | caprate (10:0) | 1.65 | 1.48 | 0.0274 | 0.0325 | 1.0080 | 1.4871 |
| 1052 | Lipid | Polyunsaturated Fatty Acid (n3 and n6) | docosapentaenoate (n3 DPA; 22:5n3) | 1.45 | 1.37 | 0.0277 | 0.0326 | 0.9218 | 1.2634 |
| 2747 | Xenobiotics | Xanthine Metabolism | 7-methylxanthine | 0.81 | 0.61 | 0.0290 | 0.0340 | 1.4468 | 0.8764 |
| 1391 | Lipid | Inositol Metabolism | myo-inositol | 0.87 | 0.88 | 0.0293 | 0.0342 | 1.0789 | 0.9455 |
| 179 | Amino Acid | Phenylalanine and Tyrosine Metabolism | thyroxine | 0.95 | 0.94 | 0.0296 | 0.0344 | 1.0667 | 1.0056 |
| 303 | Amino Acid | Methionine, Cysteine, SAM and Taurine Metabolism | alpha-ketobutyrate | 1.75 | 1.48 | 0.0300 | 0.0347 | 0.9656 | 1.4328 |
| 2749 | Xenobiotics | Xanthine Metabolism | 5-acetylamino-6-formylamino-3-methyluracil | 0.80 | 0.51 | 0.0304 | 0.0350 | 1.2637 | 0.6425 |
| 174 | Amino Acid | Phenylalanine and Tyrosine Metabolism | 3-phenylpropionate (hydrocinnamate) | 0.81 | 0.67 | 0.0326 | 0.0373 | 2.1967 | 1.4678 |
| 1540 | Lipid | Lysolipid | 1-linoleoyl-GPI (18:2)* | 0.82 | 0.78 | 0.0326 | 0.0373 | 1.1479 | 0.8917 |
| 751 | Peptide | Polypeptide | bradykinin, des-arg(9) | 0.84 | 0.68 | 0.0329 | 0.0374 | 1.5525 | 1.0516 |
| 399 | Peptide | Gamma-glutamyl Amino Acid | gamma-glutamylisoleucine* | 0.87 | 0.81 | 0.0342 | 0.0387 | 1.1889 | 0.9599 |
| 63 | Amino Acid | Histidine Metabolism | imidazole propionate | 0.87 | 0.90 | 0.0344 | 0.0388 | 1.4112 | 1.2745 |
| 1217 | Lipid | Fatty Acid Metabolism (also BCAA Metabolism) | propionylglycine | 0.78 | 0.77 | 0.0349 | 0.0391 | 1.0967 | 0.8400 |
| 2614 | ofactors and Vitami | Tocopherol Metabolism | gamma-tocopherol | 0.82 | 0.66 | 0.0359 | 0.0401 | 1.4611 | 0.9659 |
| 2710 | Xenobiotics | Benzoate Metabolism | methyl-4-hydroxybenzoate | 49.34 | 30.31 | 0.0363 | 0.0404 | 0.9339 | 28.3049 |
| 395 | Peptide | Gamma-glutamyl Amino Acid | gamma-glutamylglutamate | 0.80 | 0.77 | 0.0367 | 0.0406 | 1.2617 | 0.9684 |
| 1798 | Lipid | Steroid | 5alpha-androstan-3beta,17beta-diol disulfate | 0.86 | 0.87 | 0.0375 | 0.0413 | 1.4057 | 1.2287 |
| 1853 | Lipid | Secondary Bile Acid Metabolism | glycodeoxycholate | 1.79 | 1.44 | 0.0386 | 0.0423 | 0.9031 | 1.3019 |
| 39 | Amino Acid | Glutamate Metabolism | glutamine | 1.07 | 1.06 | 0.0387 | 0.0423 | 0.9626 | 1.0249 |
| 1064 | Lipid | Polyunsaturated Fatty Acid (n3 and n6) | docosapentaenoate (n6 DPA; 22:5n6) | 0.86 | 0.81 | 0.0397 | 0.0432 | 1.2608 | 1.0182 |
| 1826 | Lipid | Steroid | etiocholanolone glucuronide | 0.88 | 0.93 | 0.0401 | 0.0434 | 1.4135 | 1.3203 |
| 2994 | Xenobiotics | Food Component/Plant | N-(2-furoyl)glycine | 0.91 | 0.41 | 0.0411 | 0.0443 | 2.3108 | 0.9588 |
| 249 | Amino Acid | Leucine, Isoleucine and Valine Metabolism | beta-hydroxyisovaleroylcarnitine | 0.88 | 0.88 | 0.0444 | 0.0477 | 1.1032 | 0.9744 |
| 696 | Peptide | Dipeptide | threonylphenylalanine | 0.81 | 0.73 | 0.0449 | 0.0480 | 1.1720 | 0.8526 |
| 1041 | Lipid | Long Chain Fatty Acid | erucate (22:1n9) | 2.39 | 1.38 | 0.0463 | 0.0493 | 1.0137 | 1.4032 |
| 404 | Peptide | Gamma-glutamyl Amino Acid | gamma-glutamylthreonine* | 0.90 | 0.90 | 0.0482 | 0.0511 | 1.0908 | 0.9776 |

*[Biochemical Name]*  Indicates compounds that have not been officially confirmed based on a standard, but we are confident in its identity.*
