# Supplementary material for: Granulocyte colony-stimulating factor alters the systemic metabolomic profile in healthy donors
Source: Metabolomics. 2016 Nov 28;13(1):2. doi: 10.1007/s11306-016-1139-x (PMC5126202; doi:10.1007/s11306-016-1139-x)
Supplement: Supplementary file 2 — Supplementary material 2 (DOCX 19 kb) [file 11306_2016_1139_MOESM2_ESM.docx]

| **Supplementary Table 3.** Top-ranked compounds associated with different pathways | | | | |  |
| --- | --- | --- | --- | --- | --- |
| *Amino acid metabolism including*  *immune-regulatory compounds* | | | *Genetics and epigenetics* | |  |
|  | cysteine-s-sulfate | ↓ |  | inosine | ↓ |
|  | tryptophan | ↓ |  | phosphate | ↓ |
|  | isovalerate | ↓ |  | N-succinyladenosine | ↑ |
|  | histidine | ↓ |  | orotidine | ↑ |
|  | guanidinoacetate | ↓ |  | pseudouridine | ↑ |
|  | 5-hydroxyindolacetate | ↓ |  | |  |
|  | N-acetyltaurine | ↑ | *Vascular biology* | |  |
|  | N-acetylmethionine | ↑ |  | guanosine | ↓ |
|  | N-acetylserine | ↑ |  | 5-hydroxyindoleacetate | ↓ |
|  |  |  |  | O-sulfo-L-tyrosine | ↑ |
| *Compounds involved in inflammatory effects* | |  |  | allantoin | ↑ |
|  | choline phosphate | ↓ |  |  |  |
|  | guanosine | ↓ | *Energy/glycogen metabolism* | |  |
|  | dihydroorotate | ↓ |  | maltose | ↑ |
|  | ergothioneine | ↓ |  | maltotriose | ↑ |
|  | gulonic acid | ↑ |  |  |  |
